# Integrated bioinformatics analysis of the effects of chronic pain on patients with spinal cord injury

**DOI:** 10.3389/fncel.2025.1457740

**Published:** 2025-02-05

**Authors:** Jinlong Zhang, Longju Qi, Yuyu Sun, Shiyuan Chen, Jinyi Liu, Jiaxi Chen, Fangsu Yan, Wenqi Wang, Qinghua Wang, Liang Chen

**Affiliations:** ^1^Department of Orthopaedic Surgery, The First Affiliated Hospital of Soochow University, Suzhou, Jiangsu, China; ^2^Department of Spine Surgery, Nantong City No.1 People's Hospital and Second Affiliated Hospital of Nantong University, Nantong, Jiangsu, China; ^3^Affiliated Nantong Hospital 3 of Nantong University Department of Orthopedic and Nantong Third People's Hospital of Nantong University, Nantong, Jiangsu, China; ^4^School of Medicine, Nantong University, Nantong, Jiangsu, China; ^5^School of Medical Imaging, Nanjing Medical University, Nanjing, Jiangsu, China; ^6^State-Owned Assets Administration Office, Nantong University, Nantong, Jiangsu, China

**Keywords:** spinal cord injury, chronic pain, disease biomarker, hub genes, inflammatory cells, drug, gene oncology, bioinformatics

## Abstract

**Background:**

Spinal cord injury (SCI) poses a substantial challenge in contemporary medicine, significantly impacting patients and society. Emerging research highlights a strong association between SCI and chronic pain, yet the molecular mechanisms remain poorly understood. To address this, we conducted bioinformatics and systems biology analyses to identify molecular biomarkers and pathways that link SCI to chronic pain. This study aims to elucidate these mechanisms and identify potential therapeutic targets.

**Methods:**

Through analysis of the GSE151371 and GSE177034 databases, we identified differentially expressed genes (DEGs) linked to SCI and chronic pain. This analysis uncovered shared pathways, proteins, transcription factor networks, hub genes, and potential therapeutic drugs. Regression analysis on the hub genes facilitated the development of a prognostic risk model. Additionally, we conducted an in-depth examination of immune infiltration in SCI to elucidate its correlation with chronic pain.

**Results:**

Analyzing 101 DEGs associated with SCI and chronic pain, we constructed a protein interaction network and identified 15 hub genes. Using bioinformatics tools, we further identified 4 potential candidate genes. Gene Ontology (GO) and Kyoto Encyclopedia of Genes and Genomes (KEGG) pathway analyses revealed a strong correlation between SCI and chronic pain, particularly related to inflammation. Additionally, we examined the relationship between SCI and immune cell infiltration, discovering a significant link between SCI and T cell activation. This is notable as activated T cells can cause persistent inflammation and chronic pain. Lastly, we analyzed the hub genes to explore the transcription factor network, potential therapeutic drugs, and ceRNA networks.

**Conclusion:**

The analysis of 15 hub genes as significant biological markers for SCI and chronic pain has led to the identification of several potential drugs for treatment.

## Introduction

Spinal cord injury (SCI) is a severe systemic condition of the central nervous system (CNS), leading to significant motor, sensory, and autonomic impairments (Tansley et al., 2022; Wang et al., 2022). According to a statistical study, as of 2019, the leading causes of SCI were falls and road injuries (Quadri et al., 2018). The injury can be classified into two categories: primary and secondary (Yang et al., 2020). The former is a mechanical injury to the cord, while the latter is the consequence of cell and biological reactions to the primary injury. Secondary injury usually involves the immune system, nervous system, vascular system, and other systems, including hemorrhage, ischemia, oxidative stress, inflammatory reaction, neural cell death, demyelination, and scar formation (Hu et al., 2023). The primary injury mechanisms can be broadly classified as follows: (a) impact plus persistent compression; (b) impact alone; (c) distraction; and (d) aceration/transection (Sterner and Sterner, 2023). While the secondary injury can be divided into immediate, acute, intermediate, and chronic phases. The immediate phase of the injury commences immediately following the initial incident and persists for ~2 h. The acute phase is characterized by the immediate consequences of the injury, including traumatic axon rupture, rapid neural and glial cell death, and spinal shock. The intermediate phase, which occurs between 2 and 6 weeks after SCI, is characterized by the continued maturation of astroglia scarring, the formation of axonal regeneration sprouts, and the development of cysts and syrinxes. Subsequently, the chronic phase commences 6 months after the initial injury and persists indefinitely. Various treatment options, such as hydrogels, 3D printing, stem cells, and extracellular vesicle (EV) vessels, have been proposed for managing SCI. However, challenges remain in post-treatment due to reduced axonal growth, insufficient repair of endogenous cells, and the presence of inhibitory molecules at the injury site (Liu et al., 2021). Individuals with SCI often face secondary physical and psychological complications, including increased rates of depression, anxiety, and a diminished quality of life (Hearn and Cross, 2020).

Chronic pain is defined as pain persisting for 3 months or more, either continuously or intermittently (Treede et al., 2019). A study conducted by the US Centers for Disease Control and Prevention (CDC) indicates that the prevalence of chronic pain ranges from 11% to 40%, with an estimated point prevalence of 20.4%. Chronic pain is categorized into nociceptive, neuropathic, nociplastic, mixed, and cancer pain, all contributing to patient discomfort. The societal and individual impact of chronic pain is significant, with a considerable financial burden on society. Furthermore, patients with chronic pain are frequently associated with disease-specific alterations in the peripheral nervous system and CNS, along with a multitude of decrements in quality of life. It impairs an individual's capacity to work, gives rise to financial ramifications, affects biological processes in dynamic ways, including peripheral and central sensitization, the formation of new neural connections, and pathology-specific brain alterations (Cohen et al., 2021). Consequently, it is imperative to treat patients with chronic pain. Research underscores the importance of treating chronic pain, which is influenced by physical, psychological, and social factors, such as age, gender, ethnicity, lifestyle, behavior, and mental health (Mills et al., 2019). Treatments include non-pharmacological interventions like physical exercise, weight management, good sleep routines, and stress management, as well as pharmacological therapies such as muscle relaxants, non-steroidal anti-inflammatory drugs (NSAIDs), paracetamol, and opioids (Fitzcharles et al., 2021).

Chronic pain is prevalent following SCI and includes primary categories such as neuropathic, nociceptive, and other unspecified pains, as well as secondary categories like musculoskeletal, visceral, discogenic, myofascial, sacroiliac, and zygapophyseal (facet) joint pain (Hunt et al., 2021). It is estimated that between 60% and 80% of individuals with SCI report high rates of chronic pain, with one-third experiencing intense pain (Shoup et al., 2023). Numerous studies have established a causal relationship between SCI and chronic pain. For instance, Jonghoon et al. found that SCI may induce maladaptive changes in nociceptive synaptic circuits in the injured spinal cord, enhancing regional hyperexcitability in the nervous system, and leading to chronic pain (Kang et al., 2020). Additionally, Andrew et al. demonstrated that deleting miR-155 following SCI can improve mouse survival and reduce both spontaneous and evoked pain. Furthermore, single-cell RNA sequencing analysis of human spinal cord microglia identified ApoE as being associated with chronic pain (Gaudet et al., 2021). These findings indicate a strong association between SCI and chronic pain. However, most studies focus primarily on the cellular or protein level rather than the molecular level and involve numerous variables, leading to inconclusive results on how SCI affects chronic pain. Thus, it is essential to elucidate the molecular mechanisms by which SCI influences chronic pain and to identify potential markers that can reduce the likelihood of chronic pain in SCI patients.

Bioinformatics has evolved over an extended period to elucidate biological phenomena by applying information science and statistics methodologies. This approach has the potential to address the proposed research challenge (Uesaka et al., 2022). For example, the Gene Expression Omnibus (GEO) is a public functional genomics data repository that supports the submission of data in accordance with the Minimum Information. Gene Ontology (GO) knowledgebase offers a comprehensive and structured representation of gene function, accessible to computer-based analysis and applicable to genes from any cellular organism or virus, including molecular function (MF), cellular component (CC), and biological process (Aleksander et al., 2023). The KEGG database has been developed as a computer model of biological information systems, which are represented in terms of molecular interaction and reaction networks (Kanehisa et al., 2023). Protein-protein interactions (PPIs) constitute a pivotal element of the subcellular molecular networks that underpin cellular functionality (Tomkins and Manzoni, 2021), which facilitates the study of differential protein complex formation and signal flow through networks in response to changing internal and external conditions or stimuli (Jia and Wu, 2023).

Transcriptomics is the study of gene expression at the RNA level, providing a genome-wide view of the molecular mechanisms involved in specific biological processes (Dong and Chen, 2013). It is a widely used technique for elucidating biosynthetic pathways and molecular mechanisms, which support the validation of products and the entrance of molecules into clinical trials (Maldonado-Carmona et al., 2019). In order to gain insight into the potential relationship between genes and drugs, we consulted the Drug-Gene Interaction Database (DGIdb, www.dgidb.org), which provides information on drug-gene interactions, and druggable genes drawn from a range of sources, including publications, databases, and other web-based resources (Freshour et al., 2021). MicroRNAs (miRNAs) are RNAs ~22 nucleotides in length that originate from longer primary miRNA (pri-miRNA) transcripts, which contain one or more hairpins (Shang et al., 2023). Competing endogenous RNAs (ceRNAs) have been identified as a significant category of post-transcriptional regulators, which modulate gene expression through a microRNA-mediated mechanism (Wang et al., 2016). However, the ceRNA network based on SCI and spinal cord injury has yet to be reported.

In this study, we identified a genetic relationship between SCI and chronic pain, advancing our understanding of the connections between these conditions. We discovered several critical signaling pathways and gene networks linking SCI to chronic pain. Through protein-protein interaction network analysis, we identified 15 hub genes in SCI that can serve as biomarkers for exploring their roles in disease development and progression. Using the LASSO regression algorithm, we identified four potential candidate genes from these hub genes, which may significantly aid in diagnosing SCI-related chronic pain. Additionally, we screened several drugs that have regulatory relationships with these four candidate genes. These promising mechanisms, candidate genes, and drugs hold potential for improving treatment outcomes for patients with SCI and chronic pain.

## Methods

### Data acquisition and processing

We downloaded two datasets such as GSE151371 and GSE177034 from the GEO database. The GSE151371 dataset contained 38 patients suffering from a traumatic SCI and 10 healthy individuals without a history of CNS disease. The GSE177034 dataset contained 49 patients with chronic pain. The basic information about the patients is put in the Supplementary material.

### DEG analysis and intersection

Background correction, normalization, and gene symbol conversion were performed on the SCI dataset and chronic pain dataset (GSE151371 and GSE177034). Later, DEGs in the SCI and chronic pain datasets were identified using the limma package of R software. Therefore, DEGs in SCI dataset were screened according to the thresholds of *P*-value ≤ 0.05 and |fold change| ≥ 2, whereas DEGs in the chronic pain dataset were identified according to the thresholds of *P*-value ≤ 0.05 and |fold change| ≥ 1.2. Common DEGs between both datasets were obtained using Venn diagrams. Subsequently, the expression patterns of DEGs were visualized using volcano plots and heatmaps with the “ggplot2” package and “heatmap” package in R software, respectively.

### Gene set pathway enrichment analysis

Gene ontology (GO) and Kyoto Encyclopedia of Genes and Genomes (KEGG) methods are frequently used to assess the biological functions and the signaling mechanisms of polygenes. A *P-*value < 0.05 indicated that the difference was statistically significant.

### Identification of hub genes by protein-interaction-network analysis

The STRING database (https://string-db.org/) was used to perform protein-protein interaction (PPI) relationship. For the identification of hub genes, the PPI network was constructed using the proteins encoded by the shared DEGs between the SCI group and the chronic pain group. The genes showing the most significant correlations with others are referred to as hub ones. At first, Cytoscape was used to visualize the PPI network, and then cytoHubba was used to rank the genes and determine the prominent nodes in the PPI network for predicting hub genes. After the hub genes were screened out, GeneMANIA (https://genemania.org/) was used to analyze the correlation among these hub genes.

### GO/KEGG pathway enrichment analysis of hub genes

GO and KEGG pathway-enrichment analyses were performed to analyze the selected hub genes, of which, significant signal pathways and GO terms were identified, and the cutoff of significance level *P* value was set as < 0.025. The outcome was visualized by the circle plot of GO/KEGG in R software, indicating the enrichment degree of the pathway respectively.

### Variation analysis

To verify whether the hub genes were differentially expressed in each control group, variation analysis of each hub gene was performed in each control group to obtain a violin chart, which intuitively showed the difference in gene expression. The variation analysis was done to explore the correlation between SCI and chronic pain.

### Transcription factor enrichment analysis and regulatory network

TRRUST (https://www.grnpedia.org/trrust/) is a reliable and intuitive tool for human and mouse transcriptional regulatory networks (Han et al., 2018). Containing 8444 TF-target regulatory relationships of 800 human transcription factors (TFs), the TRRUST database can provide the key TFs for multiple genes and information on how these interactions are regulated. It was predicted that TFs could regulate hub genes based on Strusts. Finally, Cytoscape software (Shannon et al., 2003) was used to map the regulatory networks of TF–hub genes.

### Diagnostic predictive modeling and genetic screening by three well-established machine learning algorithms

Co-existing genes were further screened using three well-established machine learning algorithms: LASSO (Least Absolute Shrinkage and Selection Operator), SVM-RFE (Support Vector Machine-Recursive Feature Elimination), and RF (Random Forest). To ensure the reproducibility of these analyses, a fixed seed value of 123 was applied in both disease groups.

For initial biomarker screening, the LASSO algorithm, a logistic regression method that optimizes predictive performance by selecting key variables, was applied using the glmnet package. Genes identified by LASSO, along with hub genes from the common gene model, were considered potential candidates for the development of a diagnostic prediction model for chronic pain-associated spinal cord injury (SCI). The area under the receiver operating characteristic (ROC) curve was used to evaluate the diagnostic accuracy of four candidate biomarkers.

SVM-RFE was employed for recursive feature elimination, utilizing the “e1071” and “MSVM-RFE” packages. SVM-RFE sequentially eliminated less relevant features to identify the optimal hub gene. The results were visualized, and 10-fold cross-validation indicated that the red circle marked the maximum classification precision, with the corresponding gene sets showing the lowest cross-validation error and highest accuracy. Random Forest was used to classify significant genes using the “randomForest” package. This decision-tree-based algorithm ranked genes by importance, allowing the identification of key variables. A Random Forest model with 500 trees was constructed using the discovery cohort, and the optimal number of trees was determined based on cross-validation errors. Genes were ranked by importance, and the top 15 were plotted. A significant threshold of 0.5 was applied for each disease group to determine relevant genes.

The results from these three algorithms were then intersected, revealing four shared genes in the Venn diagram. After further refinement, a final intersection of these common genes yielded a single gene, identified as the potential diagnostic target for SCI.

### Immune infiltration analysis

CIBERSORT was used to assess the degree of immune cell infiltration according to the SCI gene expression profile. The abundance and proportion of immune infiltrating cells in each sample were presented as a violin plot using the ggplot2 package. The differences in the proportions of 22 types of immune infiltrating cells between SCI and control groups were compared using the Wilcoxon test, *P* < 0.05 was regarded to be of statistical significance, and a stacked histogram was created using ggplot2 package. Subsequently, the association among 22 types of immune infiltrating cells was visualized using a corrplot package. The ssGSEA algorithm was performed to estimate the relative composition and functions of different immune infiltrating cells based on mRNA expression data in the immune gene sets. The proportions of immune infiltrating cells were displayed by heatmaps. Finally, Spearman's rank correlation coefficient was adopted for the correlation analysis between the expression of diagnostic biomarkers and the proportion of infiltrated immune cells, and *P* < 0.05 indicated that the difference was statistically significant.

### Identification of potential drugs

The Drug-Gene Interaction Database (DGIdb, www.dgidb.org) is a web resource that provides information on drug-gene interactions and druggable genes from publications, databases, and other web-based sources and can be used to identify drugs that interact with these genes (Freshour et al., 2021). We used the DGIdb to predict the drugs and molecular compounds that can interact with four potential candidate genes. The drug–potential candidate genes interaction network was plotted using Cytoscape software (Shannon et al., 2003).

### Construction of competing endogenous RNA (ceRNA) network

Three databases such as Targetscan database, miRDB database, and miRanda database were used to predict the differentially expressed mRNA targets of differentially expressed miRNAs and perform the intersection between the predicted mRNA targets and common differentially expressed mRNAs, and the adjusted relationships between the miRNA and mRNA targets were obtained. The spongeScan database (https://spongescan.rc.ufl.edu) was used to obtain the relationships of lncRNA-miRNA and perform the intersection between the predicted lncRNA and common differentially expressed miRNA. Thus, the correlated lncRNA-miRNA pairs, and miRNA-mRNA pairs in the ceRNA network were displayed by cytoscape software.

### Single-cell dataset validates S100A8 gene

We selected the GSE189070 and GSE186421 datasets to validate the candidate target genes S100A8, S100A12, IL2RB, and NKG7 identified through a combined analysis of spinal cord injury and pain. The Seurat package (v4.4.0) in R (v4.2.1) was used to process these single-cell datasets. In GSE189070, we compared uninjured controls with samples collected 1-day post-injury, aligning with the GSE151371 dataset where blood samples were collected at a median of 23 h post-injury. The SCI 1D group in GSE189070 included 13,644 cells, and the uninjured group included 10,254 cells. In GSE186421, the pain group consisted of 3,647 cells, while the control group had 6,732 cells. Cells were filtered to retain those of interest. For GSE189070, the criteria were percent.mt < 10 and nCount_RNA < 25,000; for GSE186421, percent.mt < 15 and nCount_RNA < 100,000. Post-filtering, GSE189070 had 10,781 cells in the SCI 1D group and 7,677 cells in the uninjured group. GSE186421 had 2,783 cells in the pain group and 5,357 cells in the control group. Expression levels of the four candidate genes were visualized using ggplot2 (v3.5.1) through bubble and violin plots. Notably, S100A12 expression was absent in both datasets.

### Statistical analysis

Data in current study were listed as the mean ± standard deviation.

If the data followed a normal distribution, we employed a two-tailed unpaired Student's *t*-test to evaluate the disparities between the two groups. For populations that did not exhibit a normal distribution, a Mann-Whitney U unpaired test was utilized. We conducted correlation analysis using GraphPad Prism (version 8.0.1) utilizing the Pearson method. All statistical analyses were performed using R software (version 4.2.0) and GraphPad Prism. Statistical significance was determined at a *P*-value below 0.05 (^*^P < 0.05; ^**^P < 0.01; ^***^P < 0.001; ^****^P < 0.0001, n.s., not significant).

## Results

### Identification of the genetic relationship between SCI and chronic pain

**Figure 1:**
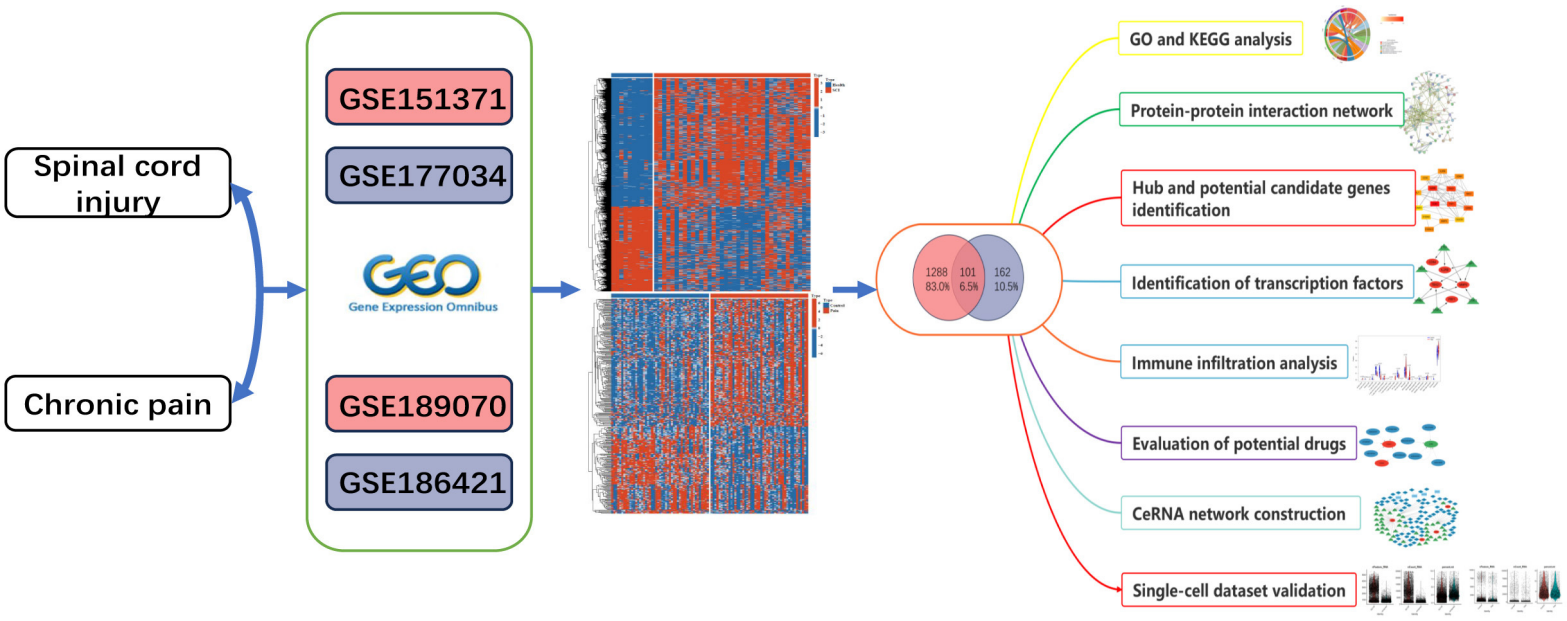
Schematic illustration of the overall flow chat of this study.

**Figure 2:**
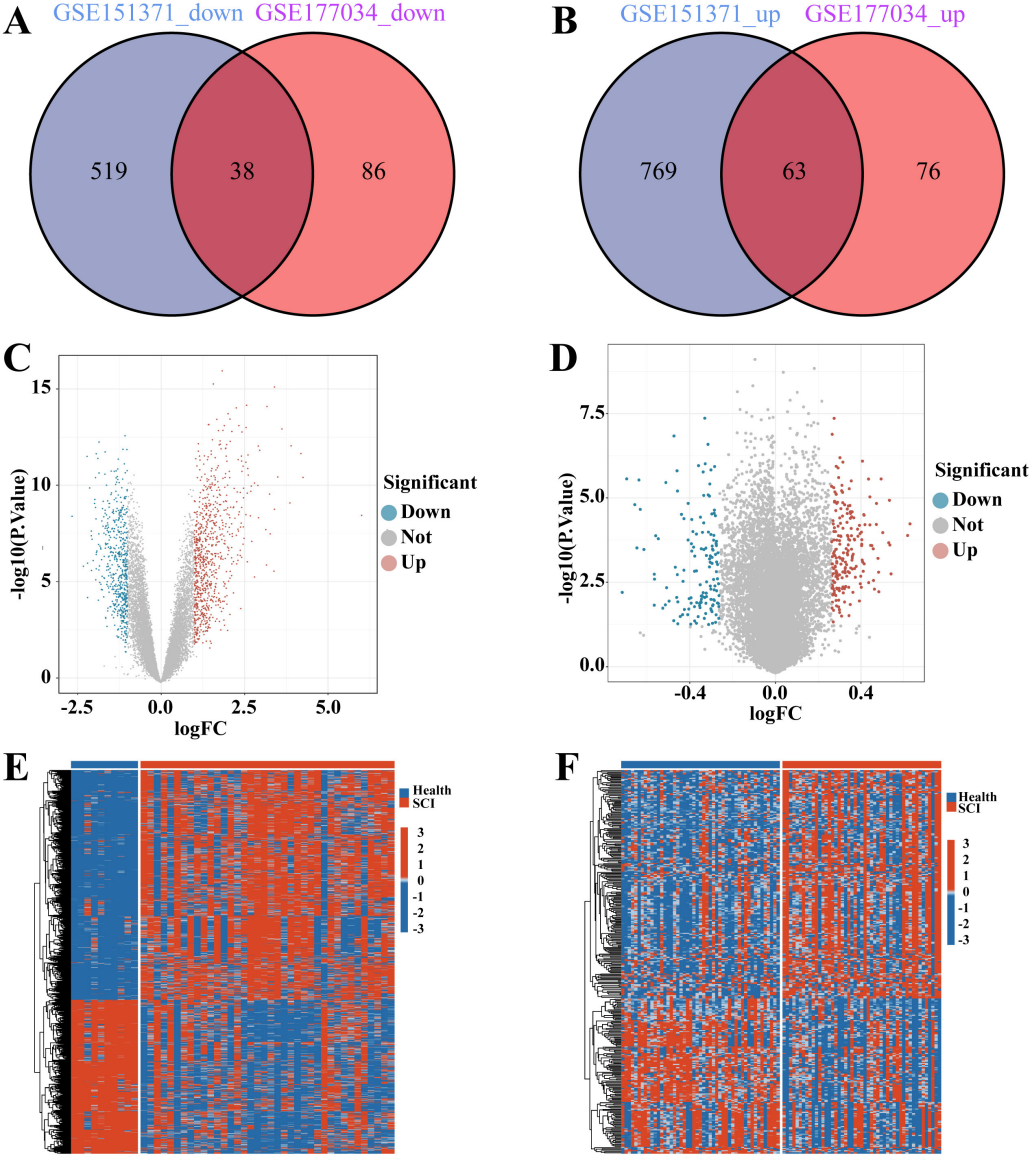
Identification of the differentially expressed genes (DEGs) between SCI and chronic pain. The Venn diagram of **(A)** down-regulated genes and **(B)** up-regulated genes in SCI and chronic pain datasets, depict the shared DEGs among two disorders. Volcano plots of **(C)** SCI and **(D)** chronic pain datasets. The DEGs expressed in **(E)** SCI/**(F)** chronic pain in normal controls and SCI/chronic pain patients were presented in the form of heatmap. The DEGs in SCI/chronic pain were the DEGs shown in **(C, D)**.

In Figure 1, we illustrate the comprehensive procedures employed in our current study. To investigate the interrelationship between SCI and chronic pain, we gathered and annotated a substantial amount of relevant data from the Gene Expression Omnibus (GEO) database to obtain both gene expression matrices and corresponding clinical information. From the SCI dataset, we identified a total of 1389 DEGs, comprising 832 up-regulated and 557 down-regulated genes (Figure 2A). Additionally, in the chronic pain group, we identified 263 DEGs through differential expression analysis, with 139 genes up-regulated and 124 genes down-regulated (Figure 2B). Using Jvenn, we identified 101 intersecting DEGs between the SCI and chronic pain groups, consisting of 63 up-regulated and 38 down-regulated genes (Figure 2). All 101 DEGs are detailed in Supplementary Table S1. The volcano plots in Figure 2 visually depict the overall transcriptional profiles of genes in the SCI and chronic pain groups, with red and blue dots indicating significantly up-regulated and down-regulated genes, respectively (Figures 2C, D). Furthermore, heatmap analyses were employed to illustrate cluster analysis and expression patterns of DEGs across different samples in the SCI and chronic pain groups, respectively (Figures 2E, F). The identification of these significant DEGs provides crucial insights for exploring disease relationships and identifying potential therapeutic targets.

### Identification of crucial signaling pathways and GO/KEGG terms by the GO enrichment analysis

**Figure 3:**
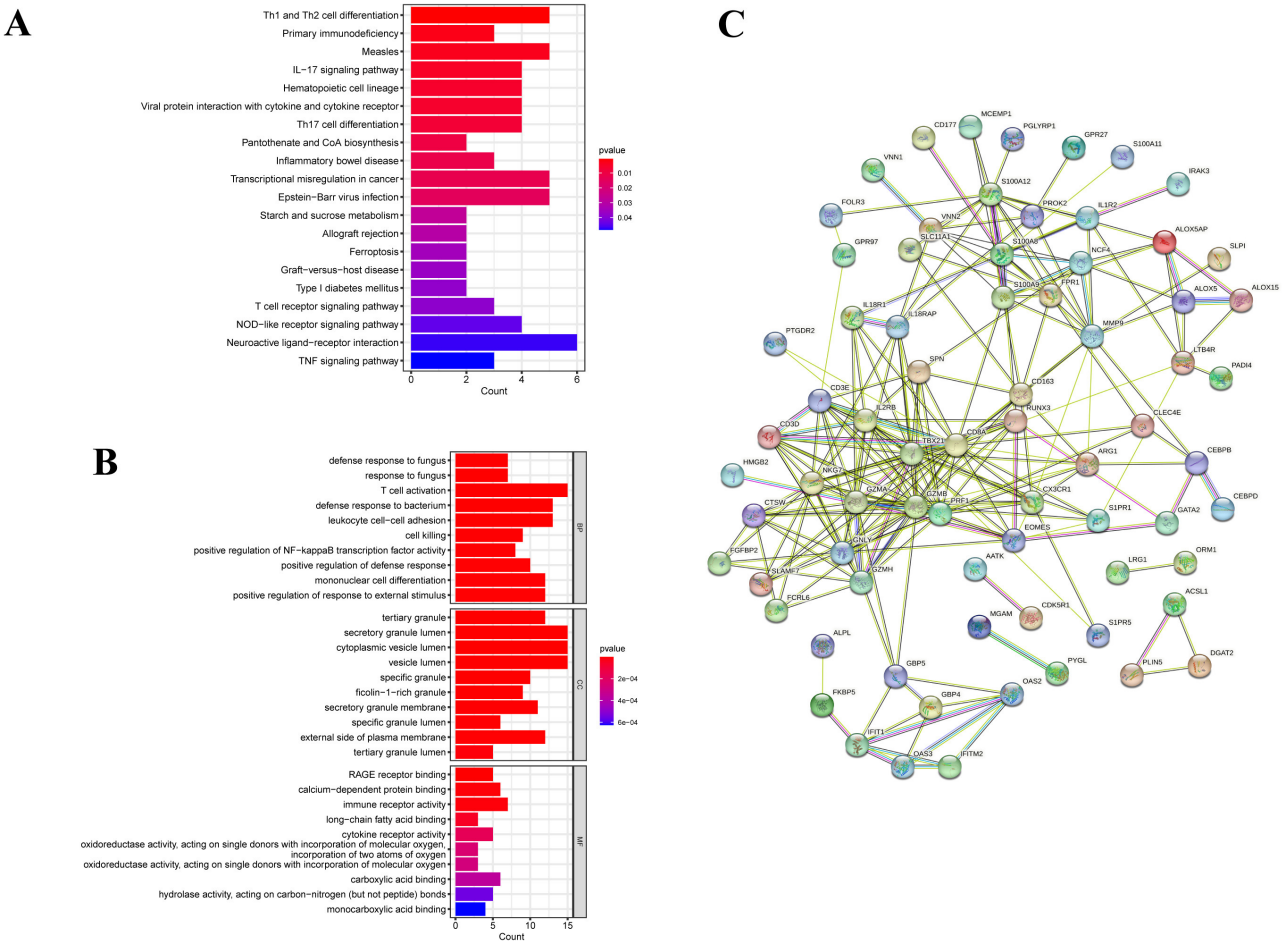
Functional pathway enrichment analysis of DEGs between patients with SCI and chronic pain. **(A)** KEGG enrichment analysis of 101 common DGEs. **(B)** GO enrichment analysis of DEGs includes biological processes (BPs), cellular components (CCs) and molecular functions (MFs). **(C)** A protein-protein interaction (PPI) network was constructed by STRING.

GO enrichment analysis characterizes the properties of genes or gene products by identifying correlations with GO terms, while KEGG analysis identifies associations between genes and signaling pathways. In Figure 3A, we conducted GO analysis and identified the top 10 enriched GO terms related to biological processes, molecular functions, and cellular components. The comprehensive results are detailed in Supplementary Table S2. Specifically, the DEGs were notably enriched in T cell activation within biological processes (BP), and in secretory granule lumen, cytoplasmic vesicle lumen, and vesicle lumen within cellular components (CC). Moreover, they showed enrichment in immune receptor activity within molecular functions (MF). These common DEGs likely play roles in immune-related functions and pathways, potentially influencing the development of chronic pain in SCI patients. KEGG pathway analysis serves as a modeling tool to illustrate how fundamental molecular or biological processes interact, revealing reciprocal influences among various diseases. In our study, the top 10 signaling pathways identified include neuroactive ligand-receptor interaction, Th1 and Th2 cell differentiation, measles, transcriptional misregulation in cancers, Epstein-Barr virus infection, IL-17 signaling pathway, hematopoietic cell lineage, viral protein interaction with cytokine and cytokine receptor, Th17 cell differentiation, and NOD-like receptor signaling pathway (Figure 3B). Our KEGG pathway analysis indicated that these 101 common DEGs are predominantly enriched in pathways related to infectious/inflammatory diseases and immune responses. This suggests a significant interconnection between SCI and chronic pain through pathways involving infection and inflammation. Additional pathway enrichment analyses are depicted in the bar diagram.

### Identification of hub genes by PPI analysis

**Figure 4:**
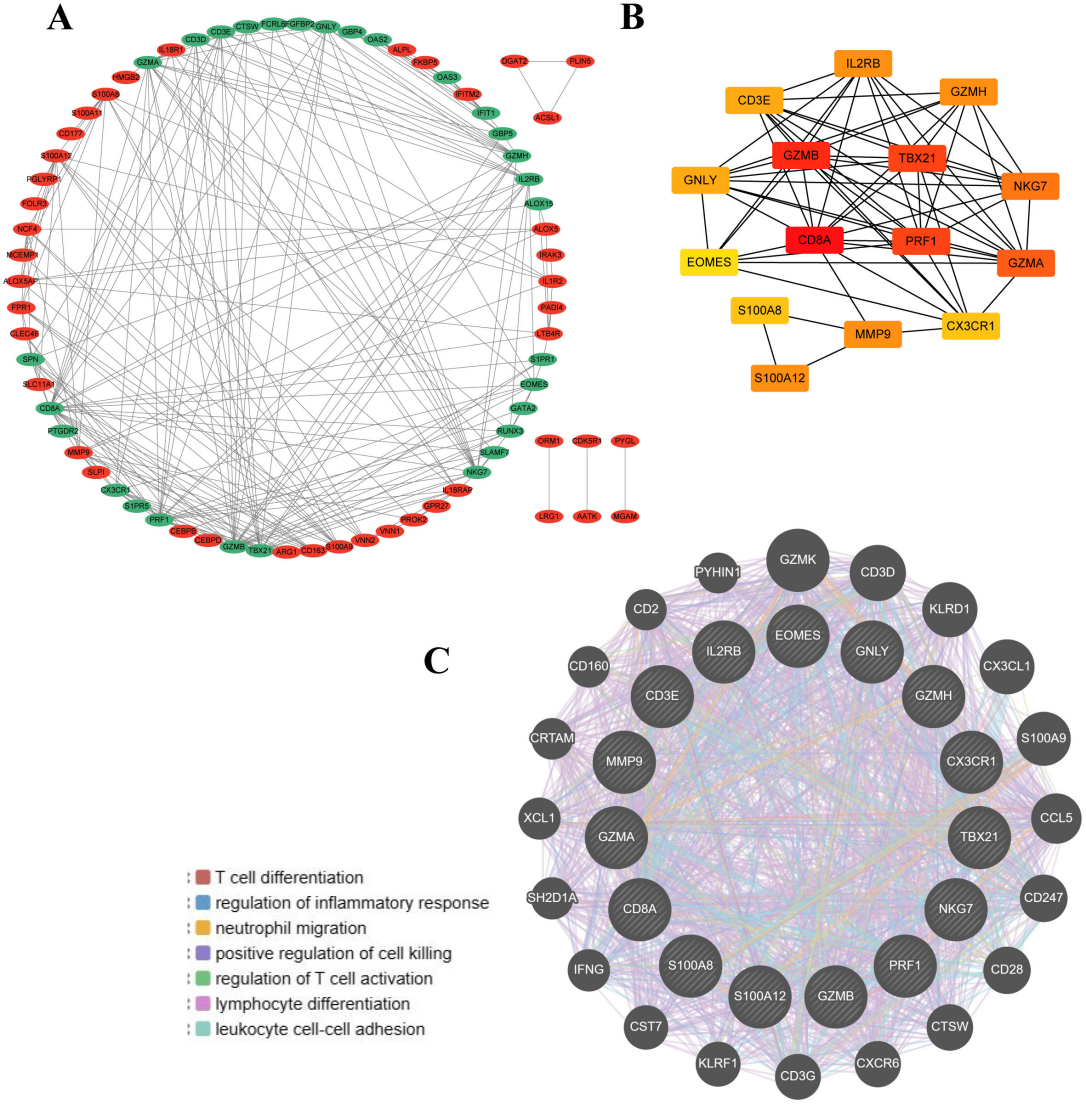
Protein–protein interaction (PPI) network and hub genes of common DEGs to SCI and chronic pain. **(A)** Cytoscape was used to visualize PPI network, containing 73 nodes and 458 edges. **(B)** 15 hub genes and their interactions with each other were shown in the network. **(C)** The gene–gene interaction network for DEGs was analyzed based on 15 hub genes by using the GeneMANIA database.

In our study, we utilized the STRING database to construct the PPI network of proteins derived from shared DEGs, highlighting functional and physical interactions between SCI and chronic pain (Figure 3C). The PPI network of common DEGs comprised 73 nodes and 458 edges, as depicted in Figure 4A. Using the cytoHubba package in Cytoscape, we identified the top 15 DEGs considered to be the most influential genes in our study. These hub genes include IL2RB, TBX21, MMP9, GZMH, GZMB, NKG7, EOMES, PRF1, GNLY, S100A12, GZMA, CX3CR1, CD8A, S100A8, and CD3E. Detailed information can be found in Supplementary Table S2. In subsequent studies, we focused on investigating the biological roles of these 15 hub genes in SCI to explore their potential mechanisms in the development and progression of the disease. These genes were selected based on their centrality in the PPI network, suggesting their significant involvement in the pathways and processes relevant to SCI and chronic pain. The identification of hub genes from common DEGs provides critical signatures for potential biomarkers in disease. Hub genes are significant for diagnosing and prognosing conditions, and using the Cytohubba plugin, we constructed a submodule network to explore gene connectivity and proximity (Figure 4B). The submodule analysis of hub genes revealed that potential candidates such as CD8A, GZMB, TBX21, and PRF1 exhibited greater connectivity within the network, confirming their strong associations with SCI. These findings highlight their potential roles as key biomarkers for understanding and managing SCI and its related conditions. As depicted in Figure 4C, gene network analysis conducted using GeneMania unveiled 15 hub genes along with associated genes, illustrating their collective network and specific functions. These functions include T cell differentiation, regulation of inflammatory response, neutrophil migration, positive regulation of cell killing, regulation of T cell activation, lymphocyte differentiation, and leukocyte cell-cell adhesion. This comprehensive analysis provides insights into the interconnected roles of these genes in immune response and cellular interactions, particularly relevant to conditions such as SCI and chronic pain.

### Identification of crucial signaling pathways and GO terms by hub gene enrichment analysis

**Figure 5:**
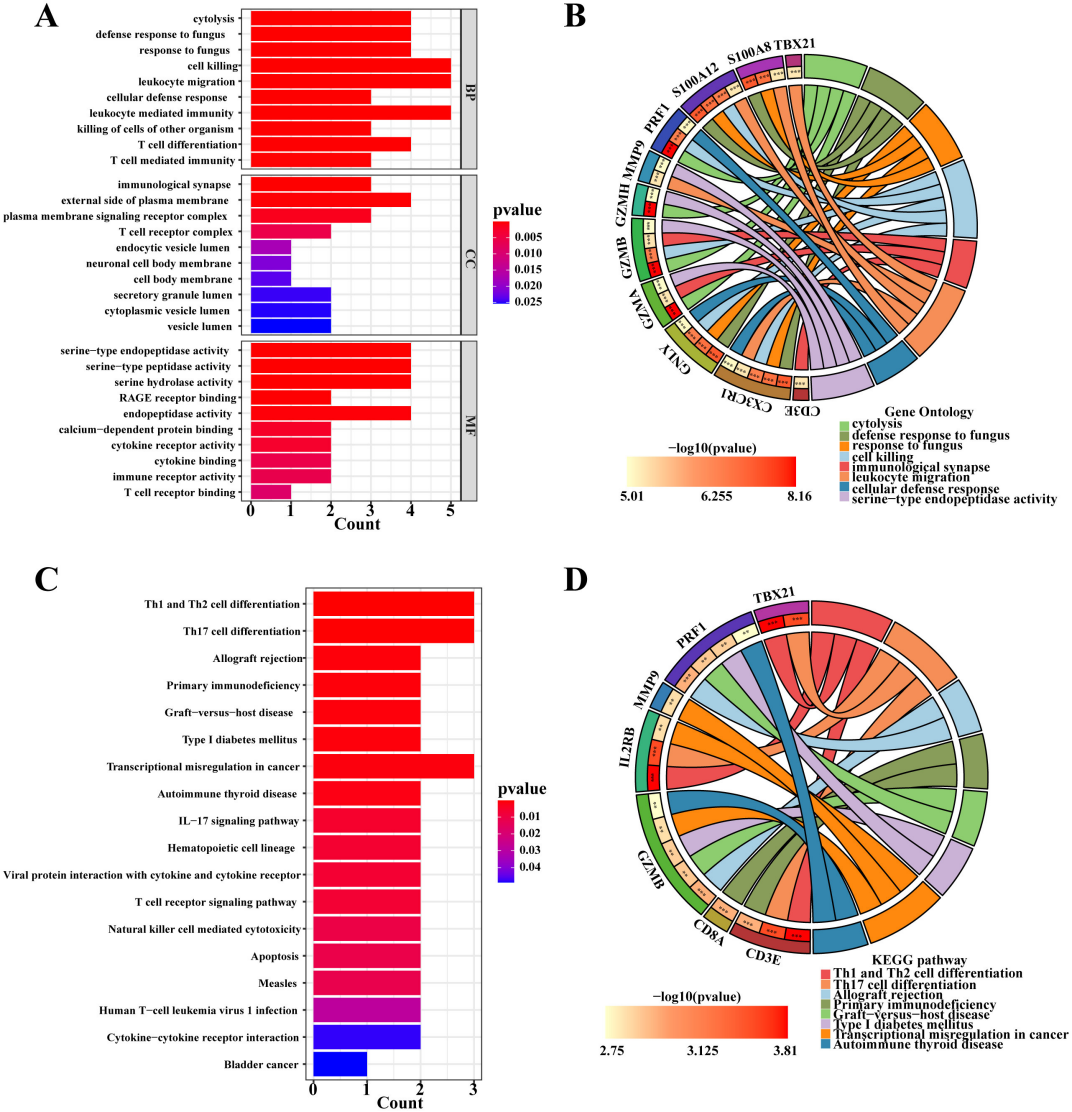
Enrichment analysis of hub genes. **(A)** GO enrichment analysis of hub genes. **(B)** The GOCircle shows the enrichment degree between the hub genes and significant GO terms. **(C)** KEGG enrichment analysis of hub genes. **(D)** The circle plot of KEGG shows the enrichment signaling pathway among hub genes.

Based on the analysis of selected hub genes, GO annotation was conducted to identify top biological processes, molecular functions, and cellular components. The results indicate significant enrichment of hub genes in cell killing, leukocyte migration, and leukocyte-mediated immunity for biological processes, external side of the plasma membrane for cellular components, and serine-type endopeptidase activity, serine-type peptidase activity, and endopeptidase activity for molecular functions (Figure 5A). The GOCircle (Figure 5B) further illustrates the strong enrichment of S100A8, S100A12, TBX21, MMP9, and CX3CR1 in processes related to leukocyte migration.

KEGG enrichment analysis identified 18 signaling pathways with a *P*-value of < 0.05 (Figure 5C). Notably, three pathways showed significant enrichment: Th1 and Th2 cell differentiation, Th17 cell differentiation, and transcriptional misregulation in cancers. The circle plot representation of KEGG results (Figure 5D) highlighted specific enrichments: CD3E and CD8A were significantly enriched in primary immunodeficiency, while GZMB and PRF1 were notably enriched in allograft rejection. Additionally, IL2RB, CD3E, and TBX21 showed significant enrichment in transcriptional misregulation in cancers and Th1 and Th2 cell differentiation pathways.

### Significantly differential expression of the hub genes between SCI and chronic pain groups

**Figure 6:**
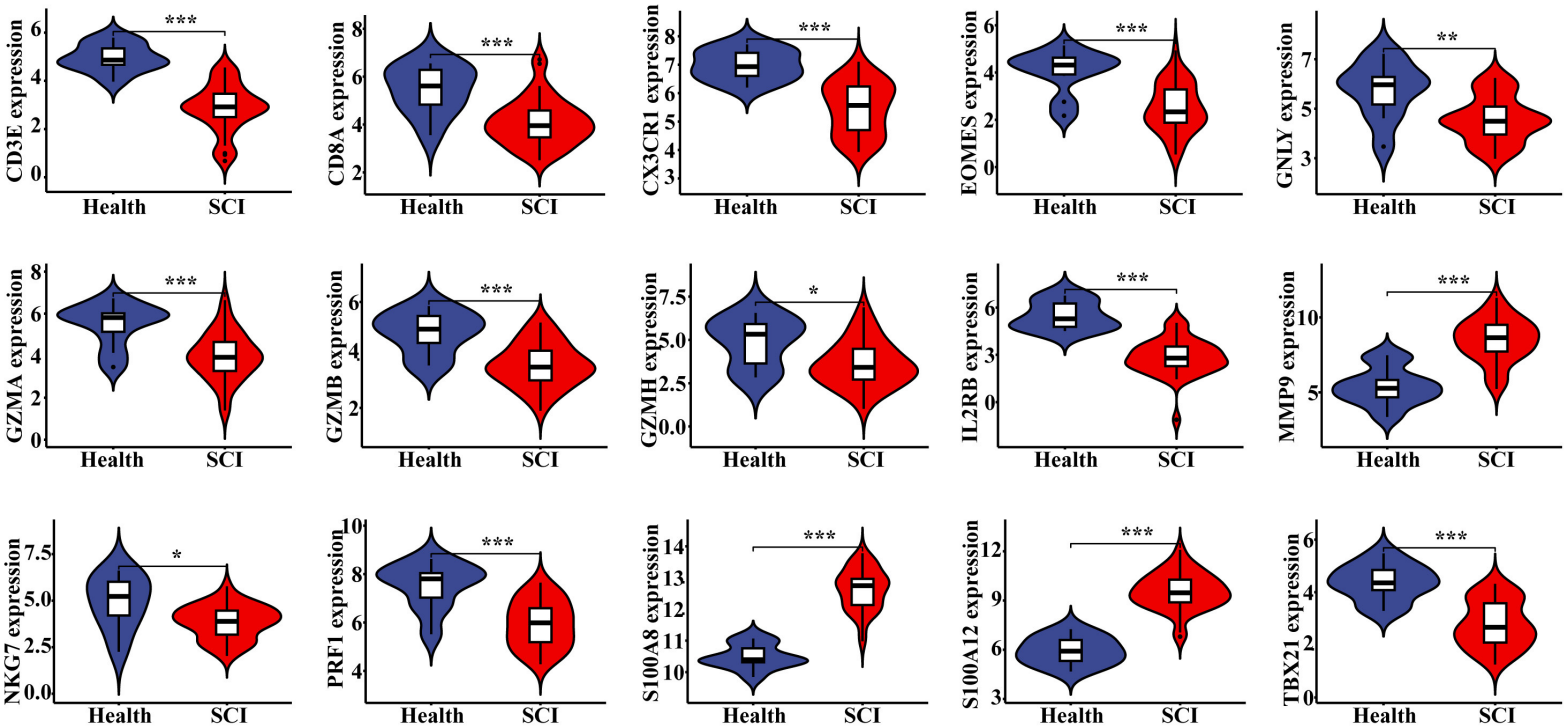
Verification of hub genes in SCI. The expression level of 15 hub genes in GSE151371, with a *P* value < 0.05, showing 3 up-regulated genes and 12 down-regulated genes. ^*^P < 0.05, ^**^P < 0.01, ^***^P < 0.001, ns, no significance.

Statistical analysis revealed significant differences in the expression levels of each hub gene between normal individuals and patients in both the SCI and chronic pain groups (Figure 6, Supplementary Figure S1). Specifically, the expressions of 3 genes were up-regulated while 12 genes were down-regulated, with a *P*-value of < 0.05 in each case. These findings underscore the differential expression patterns of hub genes associated with SCI and chronic pain, highlighting their potential roles as biomarkers or therapeutic targets.

### Identification of transcription factors correlated with hub genes

**Figure 7:**
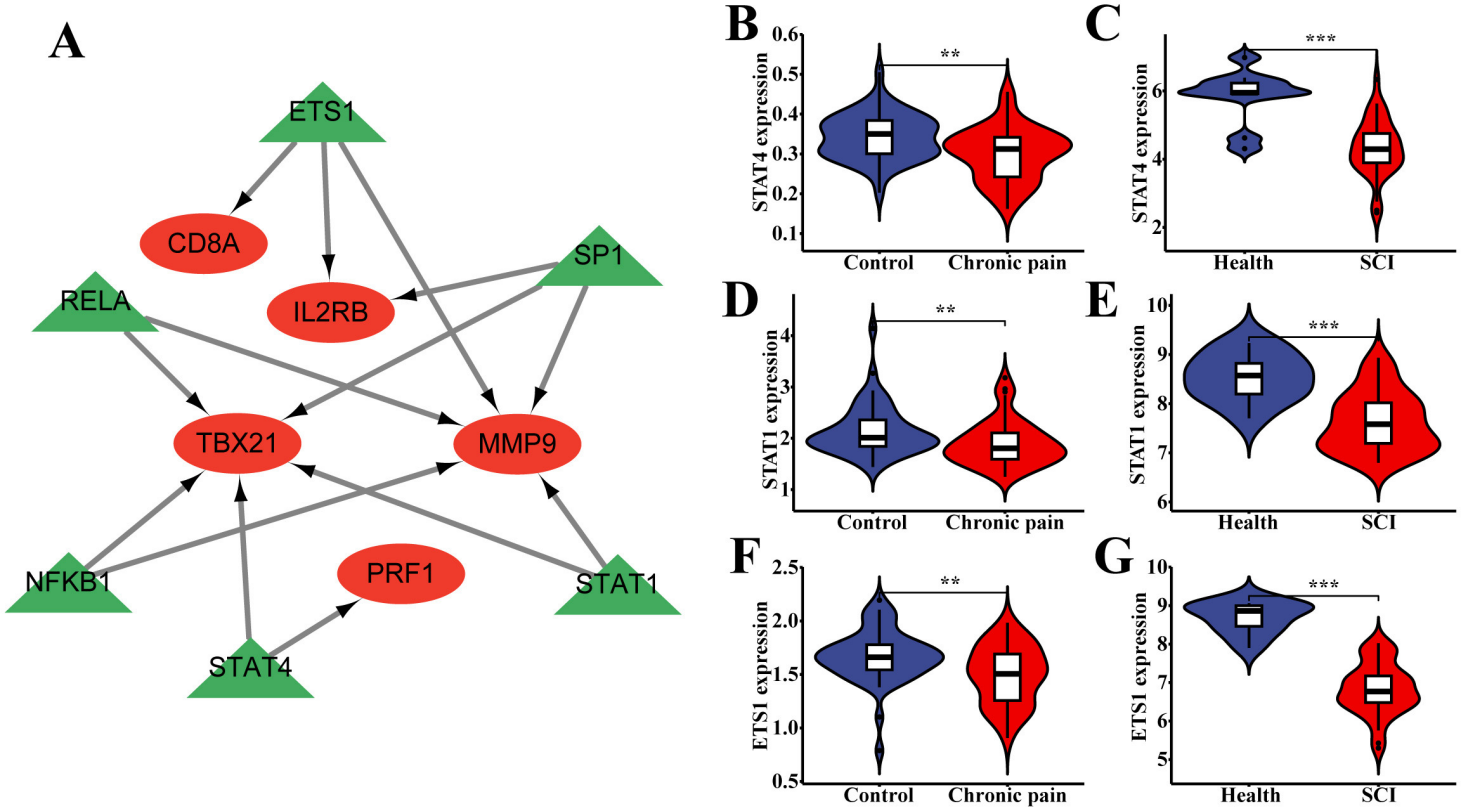
The association between transcriptions (TFs) and hub genes. **(A)** The TF regulatory network between hub genes and TFs. **(B–G)** The different expression of TFs between the normal people and patients in both SCI and chronic pain groups. ^*^P < 0.05, ^**^P < 0.01, ^***^P < 0.001, ns, no significance.

To determine which TFs among the hub genes were significantly enriched according to TRRUST, we utilized Cytoscape to visualize the TF regulatory network. The network revealed the following enrichments: CD8A with ETS1, IL2RB with ETS1 and SP1, MMP9 with ETS1, SP1, RELA, NFKB1, and STAT1, TBX21 with SP1, RELA, NFKB1, STAT4, and STAT1, and PRF1 with STAT4 (Figure 7A). These associations highlight potential regulatory relationships between the hub genes and specific TFs, providing insights into their transcriptional regulation mechanisms in the context of SCI and chronic pain.

Statistical analysis revealed significant differences in the expression of each transcription factor (TF) between normal individuals and patients in both the SCI and chronic pain groups (Figures 7B–G). Specifically, the expressions of certain TFs showed statistically significant differences with a *P*-value of < 0.05 in each case. These findings suggest differential regulation of TFs in the context of SCI and chronic pain, potentially influencing the expression profiles of hub genes and contributing to the pathophysiology of these conditions.

### Construction of disease diagnosis and risk model based on hub genes

**Figure 8:**
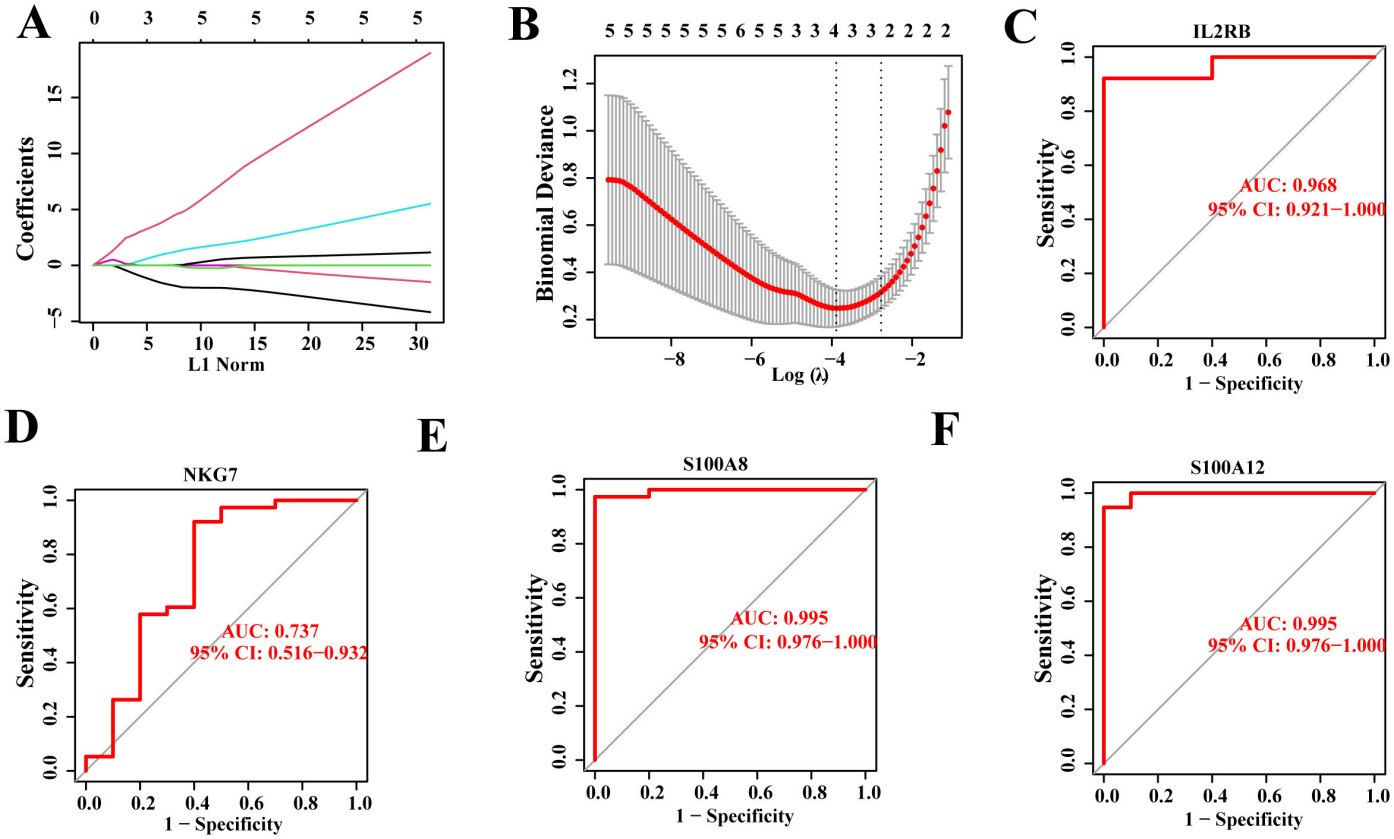
Construction of disease diagnosis and risk model. **(A, B)** Four potential candidate genes were identified out of 15 hub genes by applying LASSO regression algorithm. **(C–F)** ROC analysis of four potential candidate genes, with each >0.7.

The aforementioned analysis indicates that hub genes associated with SCI and chronic pain may influence disease progression through activation of various functions and pathways. Building upon these findings, a diagnostic model and prognostic risk model for SCI were developed based on the 15 hub genes using machine learning algorithms. Specifically, the LASSO regression algorithm identified four potential candidate genes from the 15 hub genes, demonstrating significant diagnostic potential for patients with SCI-related chronic pain (Figures 8A, B). To assess their diagnostic efficacy, receiver operating characteristic (ROC) curves were utilized to calculate the area under the curve (AUC) values for the four potential candidate genes. The ROC analysis revealed that all four potential candidate genes exhibited AUC values greater than 0.7 (Figures 8C–F), indicating their strong sensitivity and specificity in diagnosing patients with chronic pain-related to SCI. These results suggest that these genes hold promise as valuable diagnostic biomarkers for SCI-related chronic pain.

### Immune cell infiltration and correlation analysis of four potential candidate genes with invading immune cells in SCI

**Figure 9:**
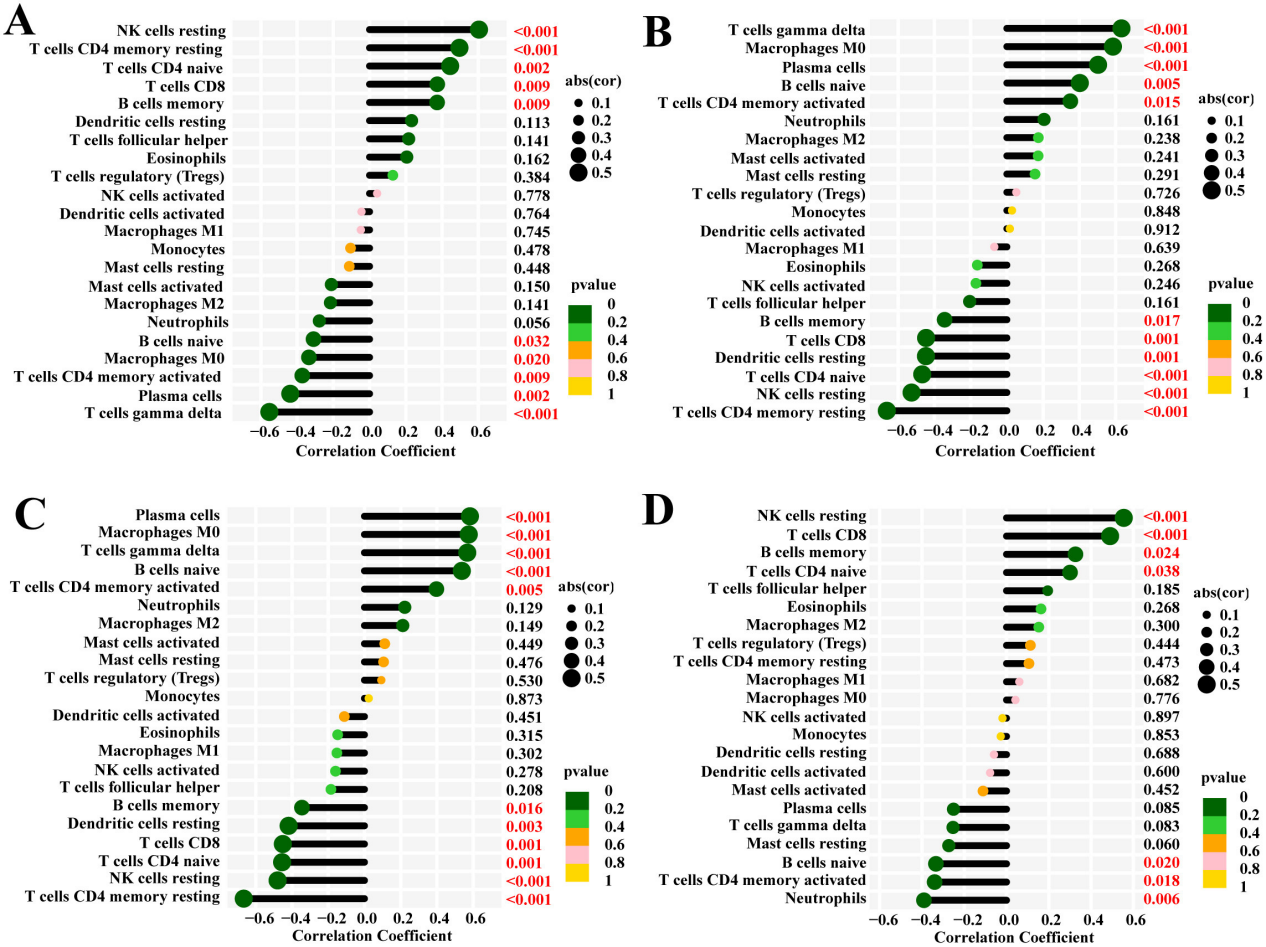
The CIBERSORT algorithm was used to examine four hub genes and their impact on chronic pain and spinal cord injury and to discover the regulation between the two conditions. **(A)** IL2RB, **(B)** S100A12, **(C)** S100A8, **(D)** NKG7.

**Figure 10:**
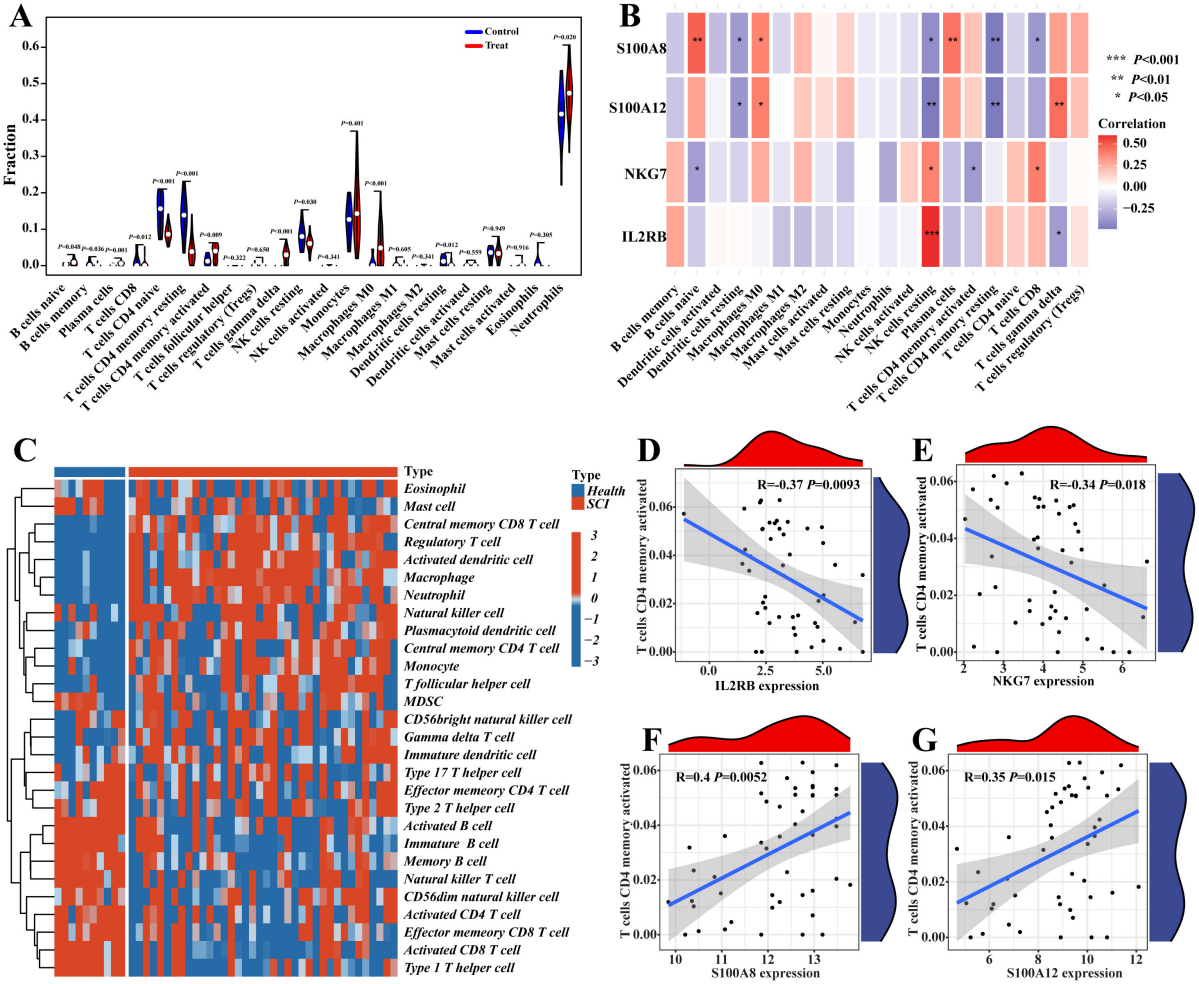
Immune cell infiltration analysis. **(A)** CIBERSORT was used to depict the differences of immune cell infiltration between SCI and control groups. **(B)** The correlations between four potential candidate genes and infiltrated immune cells. **(C)** The heatmap of immune cell infiltration landscapes of SCI was demonstrated by ssGSEA. **(D–G)** The ssGSEA was used to show the correlation between immune cells and four potential candidate genes, like T cell CD4 memory activated.

The function and pathway analysis of pathogenic genes associated with chronic pain highlighted the close association of SCI with inflammatory and immune processes. Using the CIBERSORT algorithm, we characterized immune cell profiles to explore immune regulation and the correlation between diagnostic biomarkers and immune cell infiltration in SCI (Figures 9A–D). Significant differences were observed in 10 immune cell subpopulations between the SCI and control groups among 22 types of immune cells (Figure 10A). Specifically, the SCI group exhibited higher proportions of naive B cells, activated/memory CD4^+^ T cells, gamma delta T cells, M0 macrophages, and neutrophils, while showing lower proportions of memory B cells, CD8^+^ T cells, naive CD4^+^ T cells, resting memory CD4^+^ T cells, resting NK cells, and resting dendritic cells compared to the control group (Figure 10A). Furthermore, we explored the association between the expression of four potential candidate genes (IL2RB, NKG7, S100A8, and S100A12) and the proportions of differentially infiltrated immune cells. As shown in Figure 10B, all four potential candidate genes demonstrated significant correlations with immune cell accumulation in SCI and chronic pain conditions. The landscapes of immune cell infiltration in SCI and chronic pain were further estimated using the ssGSEA algorithm (Figure 10C). Correlation analysis revealed that among the 22 types of immune cells, activated memory CD4^+^ T cells exhibited significant positive correlations with IL2RB (*r* = 0.37, *P* < 0.05) and NKG7 (*r* = 0.34, *P* < 0.05), while showing negative correlations with S100A8 (*r* = −0.40, *P* < 0.05) and S100A12 (*r* = −0.35, *P* < 0.05) (Figures 10D–G). These findings underscore the intricate immune responses in SCI and chronic pain, highlighting potential biomarkers and their interactions with immune cell dynamics.

### Identification of potential drugs that interact with potential candidate genes

**Figure 11:**
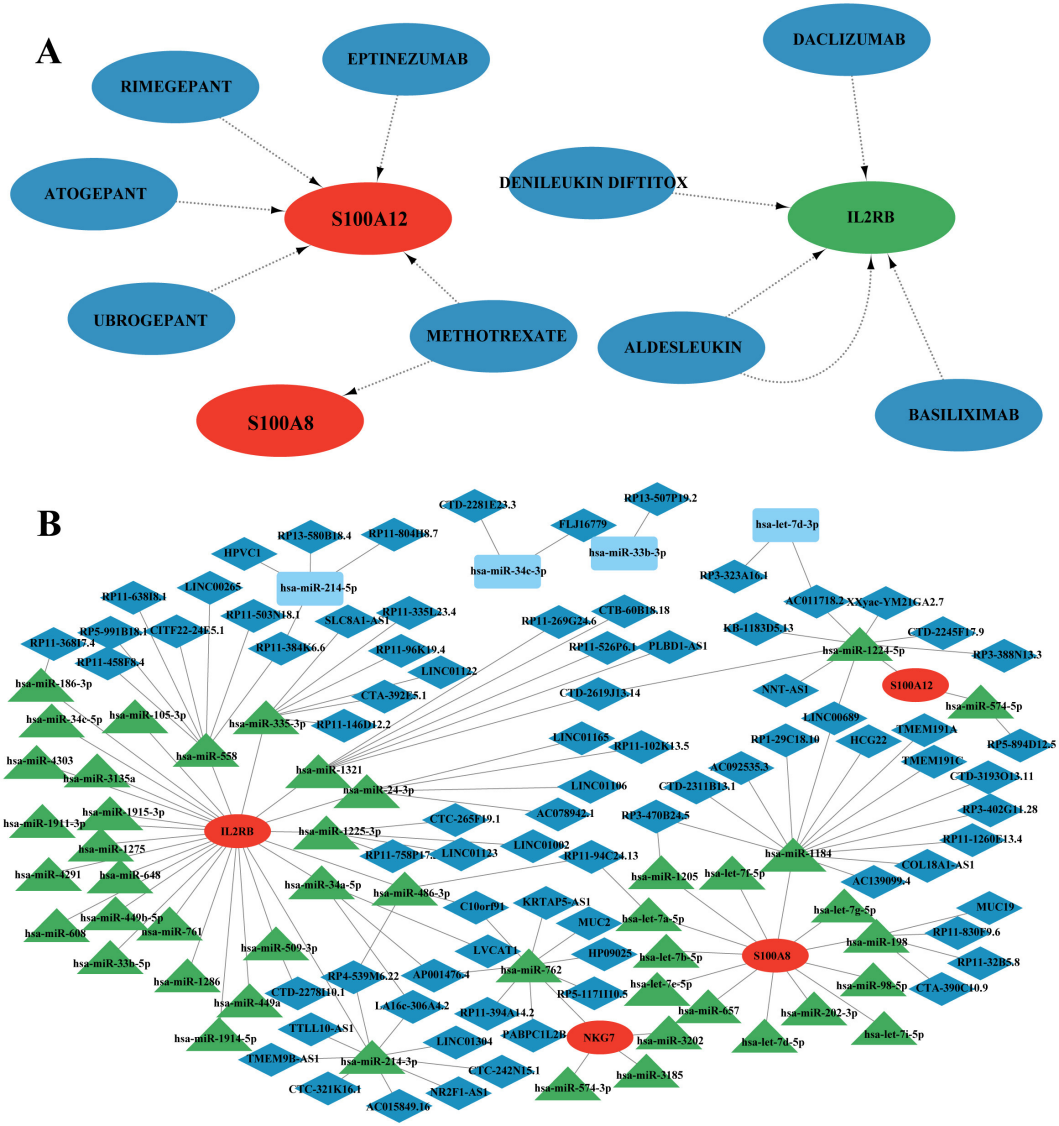
Drug interaction with potential candidate genes and ceRNAs network analysis. **(A)** Potential drug network with candidate genes. Red nodes represent up-regulation, green nodes represent down-regulation and blue nodes represent drug. **(B)** Competing endogenous RNAs (ceRNAs) network analysis of four hub genes.

The DGIdb database was employed to predict drugs or molecular compounds potentially interacting with the four identified candidate genes. A total of 9 drugs or molecular compounds were identified to have regulatory relationships with these genes, with S100A12 showing the highest number of interacting drugs among them (Figure 11A).

### Construction of ceRNA network

In this study, we investigated competing endogenous RNA (ceRNA) networks, which regulate RNA transcripts by competitively binding shared miRNAs. These networks are crucial in cancer initiation and progression, often exhibiting cancer-specific characteristics, making them potential diagnostic biomarkers or therapeutic targets. Our ceRNA network analysis revealed 4 core mRNA nodes, 78 lncRNA nodes, 45 miRNA nodes, and a total of 133 edges (Figure 11B, Supplementary Figure S2). Among the mRNAs, IL2RB exhibited the highest connectivity, forming co-expression relationships with both lncRNAs and miRNAs within the ceRNA network. Specifically, IL2RB was connected to 25 miRNAs, with 10 miRNAs involved in constructing ceRNA networks. Notably, hsa-miR-762 and hsa-miR-214-3p were individually linked with 8 lncRNAs, suggesting potential biological functions within the networks. NKG7 formed a ceRNA network exclusively with hsa-miR-762, which interacted with 7 lncRNAs. S100A8 established a ceRNA network with hsa-miR-1205, hsa-miR-1184, and hsa-miR-198, connected to 1, 13, and 4 lncRNAs, respectively. Similarly, S100A12 formed ceRNA networks with hsa-miR-1224-5p and hsa-miR-574-5p. Furthermore, we observed overlapping lncRNAs across different ceRNA networks. For instance, CTD-2619J13.14 interacted with hsa-miR-1224-5p/S100A12, hsa-miR-1321/IL2RB; LINC00689 connected with hsa-miR-1224-5p/S100A12, hsa-miR-1184/S100A8; and RP11-94C24.13 was linked to hsa-let-7a-5p/S100A8, hsa-miR-486-3p/IL2RB, among others. These findings provide insights into the intricate molecular interactions involved in SCI and chronic pain, offering potential avenues for further research into therapeutic strategies targeting ceRNA networks.

### Single-cell dataset validates S100A8 gene

**Figure 12:**
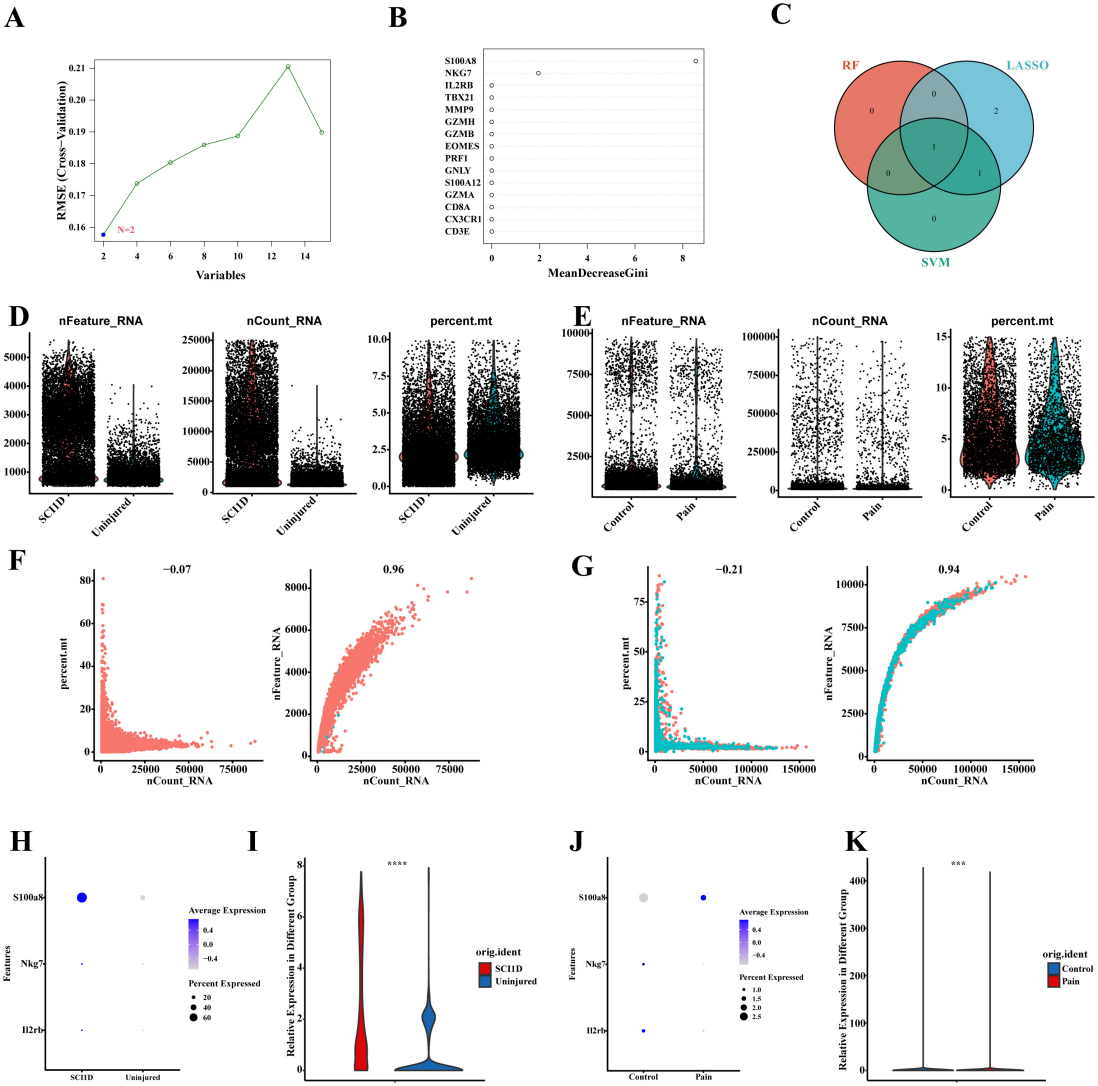
Validation of the expression of S100A8 single-cell datasets. **(A–C)** The SVM and RF machine learning methods combined with LASSO were used to select candidate genes. **(D, E)** Visualization of filtered nFeature_RNA, nCount_RNA and percent_mt results for both datasets using violin plots. **(F, G)** The first graph shows the number of RNAs expressed in the sample and the ratio of mitochondrial genes, and the second graph shows the correlation between the number of genes and the number of RNAs. **(H–K)** Visualization and differential analysis of candidate gene expression using bubble and violin plots.

Finally, we employed machine learning algorithms, including Support Vector Machine (SVM) and Random Forest (RF), to further refine the candidate genes identified through LASSO analysis. The results from the machine learning models are shown in Figures 12A, B. In the SVM analysis, we identified S100A8 and S100A12 as key genes, while in the RF analysis, genes with importance scores greater than 2 were selected. To integrate the findings, we used a Venn diagram to identify the overlap between the LASSO, SVM, and RF results. The only gene found in all three methods was S100A8 (Figure 12C).

The status of the single-cell datasets before and after applying the filtering criteria (Figures 12D, E, Supplementary Figures S3A, B) was evaluated. In both datasets, there was no significant relationship between RNA expression levels and the proportion of mitochondrial genes, which is indicative of normal sample quality. Conversely, a decrease in RNA levels along with an increase in mitochondrial gene proportion would suggest potential cell necrosis, as RNA quantity is positively correlated with gene count. These visualization assessments confirmed that the cells in the dataset are normal and viable (Figures 12F, G). The bubble and violin plots for S100A8 revealed that it was significantly upregulated in both the spinal cord injury (SCI) and pain groups, with notable differences observed in both datasets (*P* < 0.001). This trend is consistent with our previous dual-disease analysis (Figure 12H–K, Supplementary Figure S3C, D).

## Discussion

Spinal cord injury (SCI) is strongly associated with chronic pain, causing substantial physical and psychological challenges for affected individuals. Given the increasing prevalence of chronic pain following SCI, there is an urgent need to investigate its underlying mechanisms. This study employed high-throughput sequencing datasets to identify co-expressed biomarkers, aiming to elucidate the intricate connections between SCI and chronic pain and explore potential therapeutic strategies. Network analyses were conducted to pinpoint hub genes, identify potential candidate genes, and highlight regulatory TFs. Subsequent analyses evaluated potential drugs, facilitating the construction of disease models and exploring immune infiltration patterns to inform treatment strategies. Additionally, a ceRNA network was constructed to uncover regulatory pathways implicated in SCI-related chronic pain.

Transcriptomic analysis of SCI and chronic pain revealed 101 common DEGs, comprising 63 up-regulated and 38 down-regulated genes (Li J. Z., et al., 2023; He et al., 2023). However, the relationship between SCI and chronic pain, a common and significant complication, has yet to be thoroughly explored. Therefore, this study represents an initial investigation into this field, aiming to uncover the genetic correlations between these two disorders and identify potential therapeutic strategies.

To delve into the pathophysiology of these conditions, we conducted GO pathway analyses focusing on biological processes, cellular components, and molecular functions. The analysis highlighted T cell activation (15 genes), defense response to bacterium (11 genes), and leukocyte cell-cell adhesion (11 genes) as significantly enriched biological processes. T cell activation involves the recognition of exogenous peptides presented by major histocompatibility complex (MHC) molecules on antigen-presenting cells (APCs) via the T-cell receptor (TCR), triggering a cascade of signaling events essential for their physiological functions (Nel, 2002). In the context of SCI, inflammation ensues as a direct consequence, leading to tissue damage and adverse outcomes (Freyermuth-Trujillo et al., 2022). Disruption of the blood-spinal cord barrier (BSCB) facilitates T cell infiltration at the injury site, where they become activated and release cytokines like perforin, exacerbating the inflammatory response (Liu et al., 2019). Studies comparing SCI outcomes in rats and athymic nude (AN) mice (T-cell deficient) have shown superior functional recovery in the latter, underscoring the role of T cells in exacerbating post-injury inflammation (Satzer et al., 2015).

Based on GO analysis, the most significant molecular functions identified were immune receptor activity (7 genes) and calcium-dependent protein binding (6 genes). These findings are particularly relevant to the immune response triggered by inflammation following spinal cord injury (SCI), where the release of damage-associated molecular patterns (DAMPs) activates immune receptors. This activation initiates a cascade of events that recruit and activate migrating immune cells (Shen et al., 2022). In particular, immune cell receptor activation, including microglia and macrophages, has been implicated in the pathogenesis of chronic pain (Malcangio, 2019). Additionally, calcium-dependent proteins play a crucial role, potentially contributing to axonal degeneration post-SCI. The influx of calcium ions leads to calcium overload, disrupting mitochondrial function and ultimately causing neuronal apoptosis, a key contributor to chronic pain development. Further KEGG pathway analysis of the 101 common DEGs revealed significant similarities in pathway involvement between SCI and chronic pain, predominantly linking to immune-related pathways.

Following SCI, there is a notable influx of T cells at the injury site, driven by activation of the T cell receptor (TCR) and subsequent signaling pathways (Sterner and Sterner, 2023; Xu et al., 2021). Among these T cells, CD4^+^ T cells can differentiate into various subtypes, including Th1, Th2, and Th17, under the influence of inflammatory mediators. Th1 cells are recognized for producing interferon-gamma (IFN-γ) and interleukin-2 (IL-2), whereas Th2 cells typically induce interleukin-4 (IL-4), interleukin-5 (IL-5) and interleukin-13 (IL-13) production through IL-4 signaling (Luckheeram et al., 2012; Künzli and Masopust, 2023). Th17 cells, identified by their secretion of interleukin-17 (IL-17) and expression of CC chemokine receptor 6 (CCR6), are recruited to the SCI site where they become activated (Hu et al., 2016). IL-17 appears to play a regulatory role in post-SCI recovery, promoting the release of interleukin-1 (IL-1), IL-6, tumor necrosis factor-alpha (TNF-α), and other cytokines (Luckheeram et al., 2012). These cytokines, particularly IL-1, IL-6, and TNF-α, are pivotal in the secondary inflammatory response following SCI. They are released by various glial and immune cells, activating additional inflammatory cells and amplifying the overall inflammatory cascade, which can worsen SCI outcomes (Wu et al., 2021). The nucleotide-binding oligomerization domain (NOD)-like receptor (NLR) family, a subset of pattern recognition receptors (PRRs), plays a crucial role in the initial innate immune response post-injury (Platnich and Muruve, 2019). NLRP activation can lead to caspase-1-mediated pyroptosis and cleavage of IL-1β and IL-18 into their mature forms (Lin and Mei, 2020). Multiple NLRPs are involved in regulating the NF-κB signaling pathway within glial cells, further promoting the production of TNF-α, IL-6, and IL-1 (Li S., et al., 2022). The sustained presence of TNF-α, IL-1, and IL-6 is associated with neuronal hyperexcitability, contributing to chronic pain development post-SCI (Sofroniew, 2018). These cytokines sustain inflammation and exacerbate neuronal sensitization, underscoring their role in chronic pain pathophysiology following SCI.

A protein-protein interaction (PPI) network analysis was systematically conducted using 101 common DEGs to identify hub genes relevant to both SCI and chronic pain. This initiative aimed to gain insights into potential treatment strategies and biomarker discovery. Utilizing the cytoHubba package, we identified the top 15 hub genes associated with SCI and chronic pain, including IL2RB, TBX21, MMP9, GZMH, GZMB, NKG7, EOMES, PRF1, GNLY, S100A12, GZMA, CX3CR1, CD8A, S100A8, and CD3E. These genes were selected based on their significant roles in the pathogenesis of both disorders. Further analysis using the Cytohubba plugin identified IL2RB, S100A8, S100A12, and NKG7 as the top four candidate genes with pronounced relevance. These genes hold promise as therapeutic targets and valuable biomarkers for diagnostic and prognostic applications in SCI and chronic pain contexts. Their identification underscores their potential to advance understanding and treatment outcomes for these debilitating conditions.

Interleukin-2 receptor beta (IL2RB) is integral to the IL-2 receptor complex, predominantly expressed in T cells and natural killer (NK) cells. Its main role involves binding IL-2 to initiate T cell-mediated immune responses (Li et al., 2022). Upon activation, CD4^+^ T cells secrete IL-2, which promotes the differentiation of regulatory T cells (Treg cells) while inhibiting the differentiation of pro-inflammatory Th17 cells (Yuan et al., 2022). Treg cells are pivotal in promoting spinal cord repair following SCI through various mechanisms (Chen et al., 2023). In studies involving female mice lacking Treg cells, heightened activation of microglial cells was observed, triggered by colony-stimulating factor 1 (CSF1), which contributed to hyperalgesic responses or increased pain sensitivity (Olson et al., 2023). Therefore, IL2RB-mediated differentiation of Treg cells may mitigate chronic pain by modulating this inflammatory response.

S100A8 and S100A12 belong to the S100 calcium-binding cytoplasmic protein family, primarily secreted by immune cells such as monocytes, neutrophils, and dendritic cells. In the context of SCI, they function as endogenous damage-associated molecular patterns (DAMPs), initiating and amplifying the inflammatory response (Xia et al., 2018). Under hypoxic conditions, S100A8 can induce neuronal apoptosis (Ha et al., 2021). Interestingly, research suggests that prolonged use of non-steroidal anti-inflammatory drugs (NSAIDs) may prevent the transition from acute low back pain to chronic pain, potentially mediated by S100A8 released from neutrophils (Parisien et al., 2022). Moreover, S100A8 and S100A12 are recognized as core genes that could serve as therapeutic targets for various chronic pain conditions (Heida et al., 2018).

During inflammatory responses, NKG7 is secreted by both CD4^+^ and CD8^+^ T cells, where it plays a role in regulating the exocytosis of cytotoxic granules. This process enhances the synaptic efficiency of CD8^+^ T cells, facilitating the rapid elimination of target cells and thereby limiting the progression of damaging inflammation (Lelliott et al., 2022). All four potential candidate genes—IL2RB, S100A8, S100A12, and NKG7—exhibit high expression in T cells and are closely associated with T-cell activation and immune responses, indicative of inflammation. These findings are consistent with the results from GO and KEGG enrichment analyses conducted in this study.

Transitioning to the realm of TFs, which are critical proteins involved in regulating gene expression, this study explores their interconnected roles with common DEGs and miRNAs in the context of SCI and chronic pain (Matsuyama and Suzuki, 2019). Several TFs, including ETS1, SP1, RELA, NFKB1, STAT4, and STAT1, emerge as pivotal players in this complex landscape. For example, STAT1, activated by TNF-α and IFN-γ, initiates a cascade leading to nitric oxide production and PANoptosis (Karki et al., 2021). Its involvement with the promoter region of the P2Y14 receptor suggests a role in diabetic neuropathic pain (Wu et al., 2022). NF-κB transcription factors, particularly the NFKB1-RELA dimer, play a significant role in orchestrating inflammation post-SCI, particularly in microglial cells (Moynagh, 2005; Ding and Chen, 2023). Notably, the STING/TBK1/NF-κB signaling pathway in microglia has recently been implicated as a potential trigger for pain sensations in damaged nerves (Chen et al., 2018; Sun et al., 2021). Additionally, upregulation of ETS1, induced by TFs, promotes the expression of histone deacetylase 1 (HDAC1), shedding light on its role in inducing neuropathic pain following nerve injury (Zheng et al., 2023).

In this study, several miRNAs have been implicated in modulating SCI and chronic pain. For instance, down-regulated miRNA-34c-5p has been shown to mitigate the inflammatory response and alleviate hyperalgesia after nerve injury through the SIRT1/STAT3 signaling pathway (Mo et al., 2020). Additionally, reduced expression of miRNA-214-3p at the injury site post-SCI increases the release of early growth response 1 (EGR1) and colony-stimulating factor 1 (CSF1), thereby contributing to chronic pain development in affected nerves (Jiang et al., 2021). Moreover, miRNAs such as miRNA-762, miRNA-558, and miRNA-34a-5p have been associated with secondary injury or functional recovery following SCI (Deng et al., 2021). These findings underscore the intricate regulatory roles of miRNAs in modulating inflammatory responses and pain pathways in SCI and chronic pain contexts.

After analyzing four potential candidate genes, we evaluated nine substances and drugs as potential therapeutic agents for SCI. Methotrexate (MTX), known for its anti-inflammatory and immunosuppressive properties, can mitigate cell apoptosis post-SCI by regulating the endoplasmic reticulum stress (ERS) response. MTX, often administered alongside methylprednisolone (MP), helps reduce side effects and supports functional recovery of the injured spinal cord (Rong et al., 2018). As a folic acid antagonist, methotrexate (MTX) alleviates inflammation by inhibiting dihydrofolate reductase (DHFR) and thymidylate synthase (TYMS), thereby blocking the synthesis of DNA, RNA, and proteins in inflammatory cells (Zhao et al., 2022). Previously, MTX was widely used in the treatment of rheumatoid arthritis due to its potent anti-inflammatory and analgesic effects (Fautrel et al., 2022). In animal models of nerve injury, MTX has demonstrated efficacy in alleviating chronic pain (Luptovčiak et al., 2017). These findings collectively confirm the significant potential of MTX in treating chronic pain in patients with SCI. Another potential therapeutic avenue involves targeting the IL-2 receptor (IL-2R). Daclizumab, a humanized monoclonal antibody, selectively inhibits activated T cells by blocking IL-2 binding to CD25 on IL-2R. This mechanism suppresses the proliferation of activated T cells and NK cells, commonly used in multiple sclerosis treatment and as an immunosuppressant for managing post-organ transplant rejection reactions (Bielekova, 2012). Since CD25 is highly expressed on regulatory T cells (Tregs), blocking CD25 can specifically inhibit Tregs, thereby reducing the excessive immune response triggered by post-SCI inflammation (Peng et al., 2024). A recent study demonstrated that elevated levels of circulating soluble CD25 (sCD25) indicate substantial T-cell activation and are associated with an imbalance in excitatory-inhibitory regulation in the prefrontal cortex, contributing to the persistence and amplification of chronic pain (Ma et al., 2024).

Currently, calcitonin gene-related peptide (CGRP) receptor antagonists such as atogepant, ubrogepant, and rimegepant are commonly used in combination with eptinezumab, a humanized anti-CGRP monoclonal antibody, for the treatment of migraines. These treatments function by blocking CGRP-mediated inflammatory responses, vasodilation, and pain signal transmission. As a result, atogepant and eptinezumab are showing promising potential for the preventive treatment of chronic migraine (Morgan and Joyner, 2021). CGRP is released by neurons in response to pain signals, enhancing glutamate excitability in postsynaptic neurons, which contributes to central sensitization and persistent pain after injury (Löken et al., 2021). Studies have demonstrated that several CGRP receptor antagonists effectively alleviate chronic pain following SCI by inhibiting CGRP (Janzadeh et al., 2020).

This study employed bioinformatics analysis to explore the association between SCI and chronic pain. However, there are several limitations that need to be acknowledged. The findings, including core genes, transcription factor networks, and potential drug candidates, are based on data obtained from the GEO database, which has limitations in terms of dataset size and comprehensiveness for the SCI group. Therefore, it is essential to validate these findings through studies using larger and more comprehensive datasets. Furthermore, additional clinical evidence would greatly enhance the credibility and applicability of the findings. Incorporating clinical data could provide valuable insights for future research directions and potential clinical applications. Addressing these limitations will strengthen the conclusions drawn from this study and facilitate more robust investigations into the relationship between SCI and chronic pain.

In summary, this study extensively investigates the underlying correlation between SCI and chronic pain, aiming to uncover relevant pathways and molecular biomarkers for potential therapeutic targeting. Through analysis of 101 common DEGs between SCI and chronic pain, their functional roles were characterized using GO and KEGG enrichment analyses. Building upon this foundation, a PPI network was constructed to identify 15 core genes and explore their transcription factor (TF) enrichment. Subsequently, LASSO analysis pinpointed 4 potential candidate genes. Further investigation into these genes revealed 11 potential therapeutic drugs, highlighting new avenues for research and development in SCI and chronic pain treatment. We anticipate that these findings will provide valuable insights into both conditions, paving the way for innovative treatments and offering hope to patients suffering from SCI and chronic pain.

## Data Availability

The original contributions presented in the study are included in the article/Supplementary material, further inquiries can be directed to the corresponding authors.

## Supplementary material

Supplementary Figure S1 Verification of hub genes in chronic pain. The expression level of 15 hub genes in GSE177034, with a *P* value < 0.05, showing 3 up-regulated genes and 12 down-regulated genes.

Supplementary Figure S2 An intersection analysis was used to identify miRNAs from three databases, while lncRNAs were discovered through spongeScan.

Supplementary Figure S3 Validation the expression of Il2rb, Nkg7, S100a8. **(A,B)** Visualization of nFeature_RNA, nCount_RNA and percent_mt data before filtering for both datasets using violin plots. **(C,D)** Visualization of candidate gene expression using violin maps.

Supplementary Table S1-1 DEGs in GSE151371 dataset.

Supplementary Table S1-2 DEGs in GSE177034 dataset.

Supplementary Table S2-1 Candidate items of GO analysis in GSE151371.

Supplementary Table S2-2 Candidate items of GO analysis in GSE177034.

Supplementary Table S3 Clinical information of patients enlisted in current study.
