# Supplementary figures and images for: Integrated bioinformatics analysis of the effects of chronic pain on patients with spinal cord injury
Source: Front Cell Neurosci. 2025 Feb 5;19:1457740. doi: 10.3389/fncel.2025.1457740 (PMC11835904; doi:10.3389/fncel.2025.1457740)

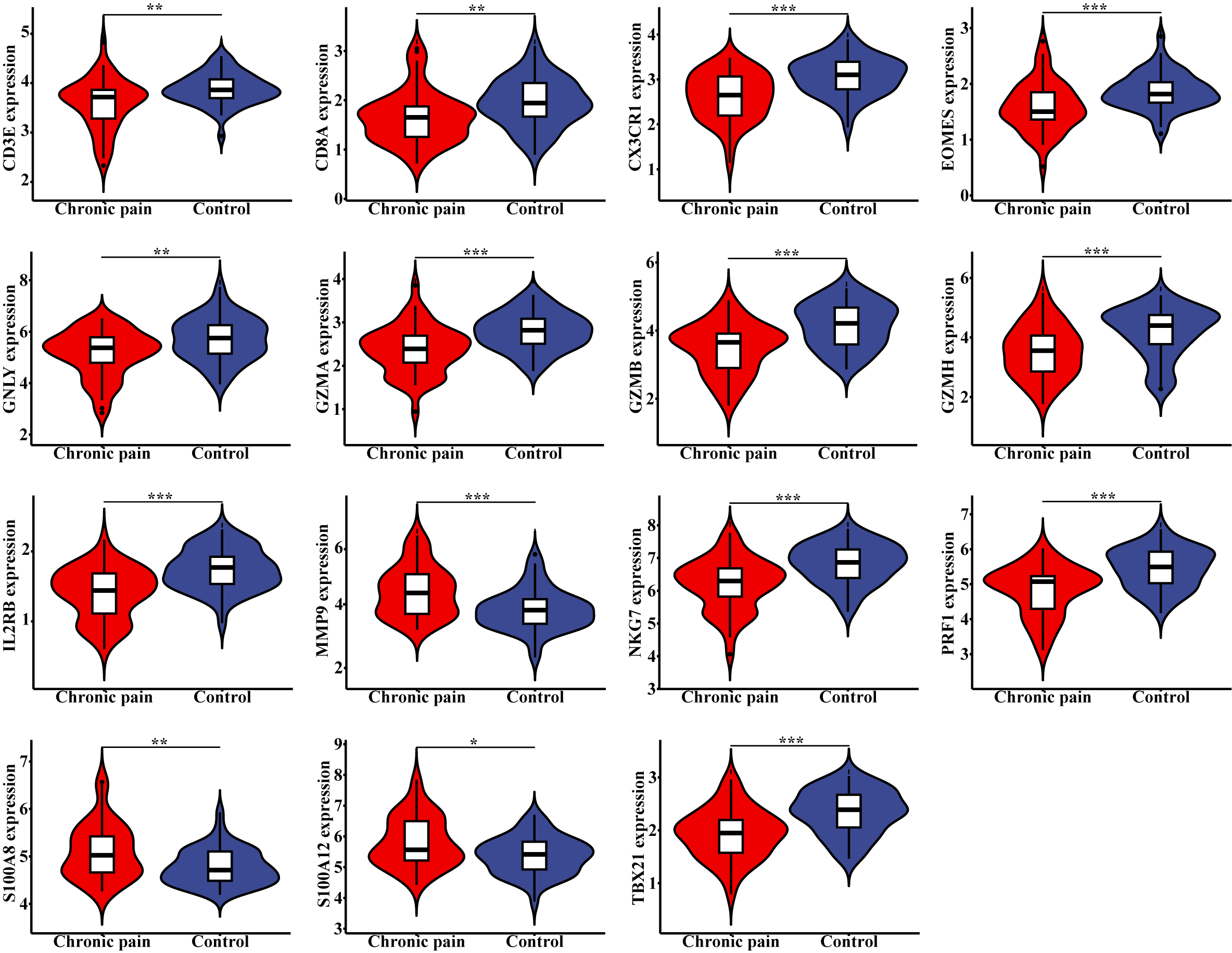

Supplement: Supplementary Figure S1 — Verification of hub genes in chronic pain. The expression level of 15 hub genes in GSE177034, with a P value < 0.05, showing 3 up-regulated genes and 12 down-regulated genes. [file Image_1.tif]

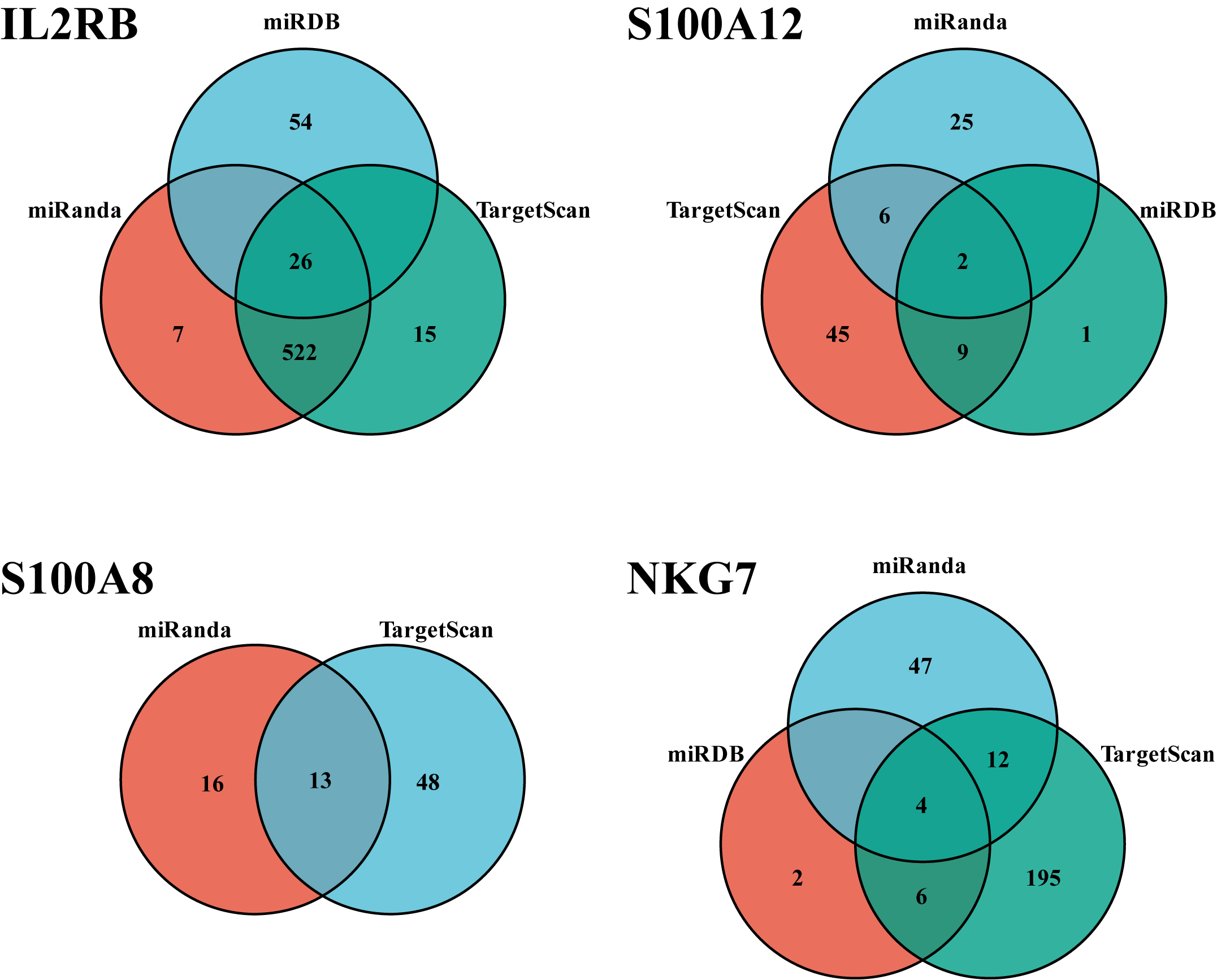

Supplement: Supplementary Figure S2 — An intersection analysis was used to identify miRNAs from three databases, while lncRNAs were discovered through spongeScan. [file Image_2.tif]

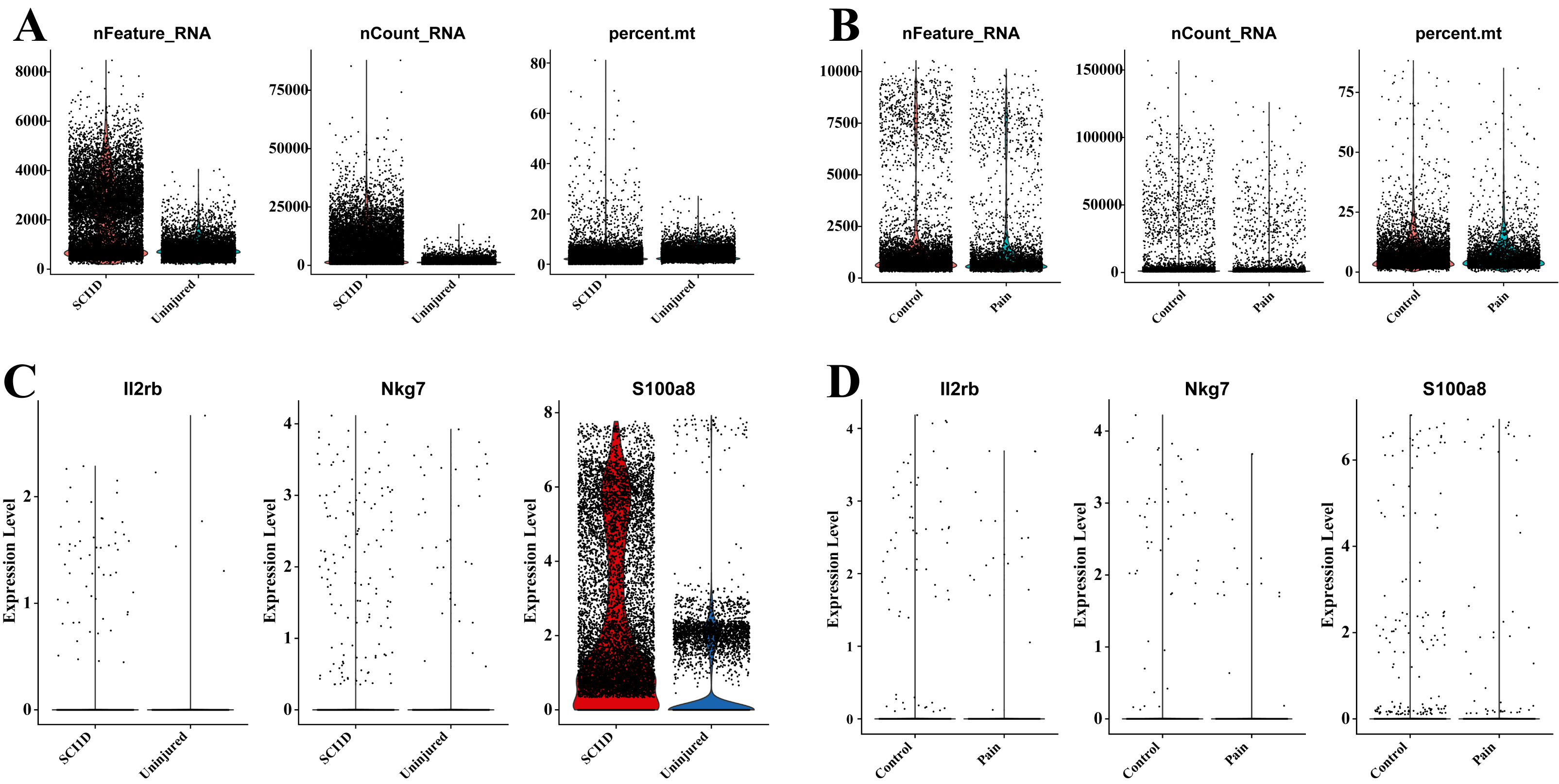

Supplement: Supplementary Figure S3 — Validation the expression of Il2rb, Nkg7, S100a8. (A,B) Visualization of nFeature_RNA, nCount_RNA and percent_mt data before filtering for both datasets using violin plots. (C,D) Visualization of candidate gene expression using violin maps. [file Image_3.tif]
