# Supplementary material for: Integrated bioinformatics analysis of the effects of chronic pain on patients with spinal cord injury
Source: Front Cell Neurosci. 2025 Feb 5;19:1457740. doi: 10.3389/fncel.2025.1457740 (PMC11835904; doi:10.3389/fncel.2025.1457740)
Supplement: Supplementary Table S1-1 — DEGs in GSE151371 dataset. [file Data_Sheet_1.pdf]

| ID | logFC | AveExpr | t | P.Value | adj.P.Val | B |
|---|---|---|---|---|---|---|
| AATK | 1.195399048 | 5.032291584 | 3.868505431 | 0.000311582 | 0.001428596 | -0.15109456 |
| ACSL1 | 1.70347575 | 11.03868059 | 7.240586112 | 2.25E-09 | 1.18E-07 | 11.2863598 |
| ADGRG3 | 1.594346327 | 7.364655821 | 5.533055358 | 1.09E-06 | 1.35E-05 | 5.276509948 |
| ALOX15 | -2.196855025 | 1.294641153 | -4.362284016 | 6.26E-05 | 0.000378734 | 1.375408906 |
| ALOX5 | 1.061371807 | 8.638700085 | 5.959000564 | 2.37E-07 | 4.06E-06 | 6.757569229 |
| ALOX5AP | 1.157313751 | 7.520712662 | 5.798403668 | 4.22E-07 | 6.32E-06 | 6.196656614 |
| ALPL | 2.182901338 | 8.17150398 | 7.392260838 | 1.30E-09 | 8.02E-08 | 11.82337303 |
| APOL6 | -1.060861236 | 7.116359429 | -5.867130232 | 3.30E-07 | 5.27E-06 | 6.436367686 |
| ARG1 | 3.903227639 | 5.781271624 | 9.440216292 | 8.74E-13 | 4.13E-10 | 18.93329083 |
| ARHGEF40 | 1.060345322 | 6.834228865 | 4.718014474 | 1.89E-05 | 0.000139509 | 2.525664025 |
| ARL4C | -1.771248923 | 5.476910121 | -6.929828392 | 6.98E-09 | 2.69E-07 | 10.18493443 |
| BASP1 | 1.315243479 | 7.997693446 | 7.422602729 | 1.16E-09 | 7.32E-08 | 11.93072436 |
| BCL9L | -1.834404306 | 2.976366768 | -4.889345666 | 1.05E-05 | 8.61E-05 | 3.092102571 |
| CA4 | 2.709240635 | 4.384949393 | 8.086329057 | 1.06E-10 | 1.19E-08 | 14.26878286 |
| CD163 | 1.740584031 | 6.378168648 | 5.373033468 | 1.93E-06 | 2.15E-05 | 4.726419994 |
| CD177 | 6.011926215 | 6.09524682 | 7.112678651 | 3.59E-09 | 1.65E-07 | 10.83312806 |
| CD3D | -1.155140119 | 4.321067635 | -3.834162681 | 0.000347292 | 0.001565256 | -0.253743935 |
| CD3E | -2.086979499 | 3.30063286 | -6.598175634 | 2.33E-08 | 6.73E-07 | 9.009816626 |
| CD8A | -1.375603814 | 4.368951604 | -4.126550857 | 0.000136058 | 0.000716173 | 0.635395814 |
| CDK5R1 | 1.165936504 | 4.00302402 | 4.630997162 | 2.54E-05 | 0.000177486 | 2.240905611 |
| CEBPB | 1.389753798 | 6.722675293 | 7.711257701 | 4.08E-10 | 3.32E-08 | 12.95028348 |
| CEBPD | 1.567834223 | 6.583253507 | 7.31469965 | 1.72E-09 | 9.66E-08 | 11.54883535 |
| CLC | -1.608978099 | 4.940018611 | -3.286789106 | 0.001837005 | 0.006301763 | -1.816957512 |
| CLEC4E | 1.315002985 | 6.671208528 | 5.673614421 | 6.60E-07 | 9.01E-06 | 5.76279605 |
| CTSW | -1.058267641 | 3.566094959 | -2.715996456 | 0.008994985 | 0.023752895 | -3.27611414 |
| CX3CR1 | -1.525859987 | 5.786178845 | -5.226748446 | 3.23E-06 | 3.25E-05 | 4.227284331 |
| DAAM2 | 1.795357737 | 2.809235986 | 3.146873465 | 0.002753091 | 0.008837453 | -2.192199897 |
| DGAT2 | 1.223095018 | 7.419959806 | 6.754523232 | 1.32E-08 | 4.36E-07 | 9.563566551 |
| DOK3 | 1.376664932 | 6.640270642 | 5.159263021 | 4.09E-06 | 3.92E-05 | 3.998344799 |
| EOMES | -1.550063246 | 2.826615717 | -4.548416397 | 3.36E-05 | 0.000225144 | 1.972621196 |
| ESYT1 | -1.260653196 | 6.277687509 | -6.048506789 | 1.71E-07 | 3.12E-06 | 7.071272538 |
| FCRL6 | -1.710660781 | 2.630373286 | -4.38744247 | 5.76E-05 | 0.000353888 | 1.455496244 |
| FGFBP2 | -1.937824727 | 3.32460349 | -5.349626795 | 2.09E-06 | 2.29E-05 | 4.646303078 |
| FKBP5 | 1.453768412 | 7.659337975 | 5.076101085 | 5.47E-06 | 5.00E-05 | 3.717463824 |
| FLOT2 | 1.435946926 | 8.161662933 | 4.538865609 | 3.47E-05 | 0.000231422 | 1.941720324 |
| FOLR3 | 3.14562386 | 4.989360904 | 6.643921302 | 1.98E-08 | 5.88E-07 | 9.171776439 |
| FPR1 | 1.184515319 | 9.89588777 | 7.198105783 | 2.63E-09 | 1.32E-07 | 11.13586281 |
| GATA2 | -1.006535381 | 1.685419815 | -3.375421451 | 0.001414977 | 0.005022731 | -1.573811112 |
| GBP4 | -1.629158265 | 4.89974471 | -7.12864759 | 3.39E-09 | 1.57E-07 | 10.88972592 |
| GBP5 | -1.27551489 | 6.925829411 | -4.563813961 | 3.19E-05 | 0.000215118 | 2.022495169 |
| GCA | 1.348608112 | 9.481594528 | 8.062640496 | 1.15E-10 | 1.27E-08 | 14.18576711 |
| GNLY | -1.19233455 | 4.815129129 | -3.62744562 | 0.000661023 | 0.00264971 | -0.860773414 |
| GPR27 | 2.432268344 | 1.748926503 | 9.923465021 | 1.65E-13 | 1.20E-10 | 20.55135919 |
| GZMA | -1.524079177 | 4.28753719 | -4.033185512 | 0.000184105 | 0.00091866 | 0.347829248 |
| GZMB | -1.230332525 | 3.879103034 | -4.445146811 | 4.75E-05 | 0.000300674 | 1.63995086 |
| GZMH | -1.286781202 | 3.878470042 | -2.834922894 | 0.006551599 | 0.018174424 | -2.988332788 |
| HAUS4 | 1.502088434 | 6.148130603 | 8.061407073 | 1.15E-10 | 1.27E-08 | 14.18144358 |
| HMGB2 | 1.440014021 | 7.032195344 | 7.509051234 | 8.50E-10 | 5.71E-08 | 12.23641636 |
| HRH2 | 1.253413625 | 6.460673635 | 5.678725361 | 6.48E-07 | 8.90E-06 | 5.780527938 |

| | | | | | | |
|---|---|---|---|---|---|---|
| IFIT1 | -1.070366982 | 4.505149941 | -2.427766099 | 0.01875553 | 0.043686856 | -3.934642578 |
| IFITM2 | 1.007677651 | 8.342837667 | 4.781353225 | 1.52E-05 | 0.000116817 | 2.73420472 |
| IL18R1 | 2.121127872 | 6.029287972 | 5.722025404 | 5.55E-07 | 7.84E-06 | 5.930884995 |
| IL18RAP | 2.220001575 | 7.750704694 | 7.027324048 | 4.90E-09 | 2.03E-07 | 10.53056962 |
| IL1R2 | 2.141281034 | 7.47051575 | 6.426893981 | 4.35E-08 | 1.08E-06 | 8.404036608 |
| IL2RB | -2.656104712 | 3.400901106 | -7.08666864 | 3.94E-09 | 1.77E-07 | 10.74093625 |
| IRAK3 | 1.902730962 | 8.622958177 | 10.14520099 | 7.75E-14 | 8.34E-11 | 21.28428536 |
| KLRC4 | -1.187341543 | 0.247063931 | -3.203865591 | 0.002337423 | 0.007704052 | -2.040649054 |
| LILRA5 | 2.413100193 | 8.118658898 | 9.08769085 | 2.99E-12 | 8.76E-10 | 17.73603079 |
| LRG1 | 1.630800353 | 6.511880752 | 7.380443974 | 1.36E-09 | 8.20E-08 | 11.78155669 |
| LSMEM1 | 1.247647864 | 3.332179073 | 5.581598405 | 9.17E-07 | 1.17E-05 | 5.444144394 |
| LTB4R | 1.429162068 | 6.168330851 | 7.276414748 | 1.98E-09 | 1.07E-07 | 11.41326358 |
| MANSC1 | 1.36831122 | 5.061865069 | 7.132256664 | 3.34E-09 | 1.57E-07 | 10.90251695 |
| MCEMP1 | 4.196619467 | 5.474772057 | 9.176875278 | 2.19E-12 | 7.50E-10 | 18.04020122 |
| MGAM | 1.622962637 | 10.22104743 | 6.852387432 | 9.25E-09 | 3.34E-07 | 9.910410198 |
| MKNK1 | 1.383129337 | 6.200134413 | 5.955171983 | 2.40E-07 | 4.10E-06 | 6.744167037 |
| MMP9 | 3.27828148 | 7.713050555 | 6.504894335 | 3.28E-08 | 8.61E-07 | 8.679765096 |
| NCF4 | 1.164613086 | 7.588203358 | 5.983045035 | 2.17E-07 | 3.79E-06 | 6.841769819 |
| NKG7 | -1.076251651 | 4.08045804 | -3.09284662 | 0.003210481 | 0.010048331 | -2.334178897 |
| NQO2 | 1.364154465 | 5.869960711 | 6.327388292 | 6.24E-08 | 1.40E-06 | 8.052699764 |
| OAS2 | -1.055012239 | 5.137984896 | -5.507229152 | 1.20E-06 | 1.45E-05 | 5.187463089 |
| OAS3 | -1.144016581 | 4.44653112 | -3.518157927 | 0.000922556 | 0.003490393 | -1.173787545 |
| ORM1 | 2.421477387 | 3.241290209 | 5.737129877 | 5.26E-07 | 7.51E-06 | 5.983388848 |
| PADI4 | 1.731783623 | 6.917650117 | 6.252326038 | 8.20E-08 | 1.73E-06 | 7.788027464 |
| PFKFB2 | 2.586349779 | 6.101120972 | 5.584374866 | 9.08E-07 | 1.16E-05 | 5.453742477 |
| PGLYRP1 | 2.379590311 | 3.584933063 | 5.799848193 | 4.20E-07 | 6.32E-06 | 6.201689609 |
| PLD4 | -1.146503981 | 1.0863629 | -3.488454743 | 0.001009171 | 0.003756329 | -1.257868001 |
| PLIN5 | 1.192046843 | 3.790940617 | 4.275451527 | 8.35E-05 | 0.000477957 | 1.100594708 |
| PRF1 | -1.609660577 | 6.261838041 | -4.91899434 | 9.45E-06 | 7.90E-05 | 3.190854892 |
| PROK2 | 1.861411399 | 7.61731502 | 8.339334309 | 4.26E-11 | 6.05E-09 | 15.15291869 |
| PTGDR2 | -1.872890071 | 1.512339498 | -4.267711905 | 8.57E-05 | 0.000487368 | 1.076222873 |
| PYGL | 1.439706811 | 9.076973013 | 8.065229583 | 1.14E-10 | 1.27E-08 | 14.19484232 |
| RUNX3 | -1.651096467 | 3.85539623 | -6.484720624 | 3.53E-08 | 9.10E-07 | 8.608427405 |
| S100A11 | 1.049993509 | 6.546633975 | 5.233031295 | 3.16E-06 | 3.19E-05 | 4.248642576 |
| S100A12 | 3.631033879 | 8.803187864 | 10.03129285 | 1.14E-13 | 1.05E-10 | 20.90854178 |
| S100A8 | 2.125167315 | 12.17971846 | 9.938657991 | 1.57E-13 | 1.20E-10 | 20.6017736 |
| S100A9 | 1.626259659 | 13.05524715 | 8.295768453 | 4.98E-11 | 6.79E-09 | 15.00101942 |
| S100P | 2.275530468 | 6.525554579 | 5.518543531 | 1.15E-06 | 1.41E-05 | 5.226462115 |
| S1PR1 | -1.054624394 | 5.551682388 | -5.181256541 | 3.79E-06 | 3.69E-05 | 4.072859771 |
| S1PR5 | -1.364873806 | 2.723509294 | -4.512820025 | 3.78E-05 | 0.00024904 | 1.857588942 |
| SCARNA20 | 1.285667049 | 0.705898184 | 5.657936435 | 6.98E-07 | 9.41E-06 | 5.708423817 |
| SH2D2A | -1.360076657 | 1.307286183 | -4.761152262 | 1.63E-05 | 0.000123205 | 2.66758071 |
| SLAMF7 | -1.036025082 | 3.962568987 | -4.772869834 | 1.56E-05 | 0.000119402 | 2.706213233 |
| SLC11A1 | 1.367557327 | 8.701410994 | 7.181110828 | 2.80E-09 | 1.38E-07 | 11.07564505 |
| SLC2A3 | 1.594261842 | 9.467069526 | 8.423831738 | 3.15E-11 | 5.10E-09 | 15.44709966 |
| SLFN5 | -1.583216428 | 5.498736231 | -7.011602414 | 5.18E-09 | 2.13E-07 | 10.47483558 |
| SLPI | 2.547619587 | 4.071505039 | 8.884911148 | 6.12E-12 | 1.43E-09 | 17.04135823 |
| SPN | -1.082495842 | 5.878176333 | -5.829591144 | 3.77E-07 | 5.86E-06 | 6.30537144 |
| TAS2R40 | 2.468217043 | 2.644123491 | 8.74878674 | 9.90E-12 | 2.13E-09 | 16.57273017 |
| TBX21 | -1.593797835 | 3.115407546 | -5.519624621 | 1.14E-06 | 1.41E-05 | 5.23018947 |

| | | | | | | |
|---|---|---|---|---|---|---|
| VNN1 | 2.723216929 | 6.366670027 | 6.262237073 | 7.91E-08 | 1.69E-06 | 7.822954694 |
| VNN2 | 1.170833202 | 10.17047505 | 7.131491012 | 3.35E-09 | 1.57E-07 | 10.89980339 |
