# Supplementary material for: Integrated bioinformatics analysis of the effects of chronic pain on patients with spinal cord injury
Source: Front Cell Neurosci. 2025 Feb 5;19:1457740. doi: 10.3389/fncel.2025.1457740 (PMC11835904; doi:10.3389/fncel.2025.1457740)
Supplement: Supplementary Table S1-2 — DEGs in GSE177034 dataset. [file Data_Sheet_2.pdf]

ID	logFC	AveExpr	t	P.Value	adj.P.Val	B
AATK	0.26952895	1.449849751	4.260719371	4.74E-05	0.002042755	-0.46347575
ACSL1	0.410738614	3.010382763	3.280983163	0.001440287	0.015877582	-3.710329226
ADGRG3	0.494730695	3.222767088	4.982965158	2.75E-06	0.000501605	2.295066163
ALOX15	-0.301693051	0.908498544	-2.429907051	0.016951111	0.081956345	-5.970459424
ALOX5	0.313850368	2.597110176	4.344721819	3.45E-05	0.001680013	-0.157139978
ALOX5AP	0.28269971	1.697198964	3.724234756	0.000329745	0.006346738	-2.31944557
ALPL	0.397067561	2.335269854	3.01495994	0.003284093	0.02694644	-4.477379554
APOL6	-0.384988268	2.209252214	-4.062283401	9.90E-05	0.003026634	-1.170681531
ARG1	0.482476536	1.012267636	3.262680508	0.001526606	0.016483588	-3.764780513
ARHGEF40	0.300034155	2.386221741	3.20525119	0.001829898	0.018384285	-3.934046118
ARL4C	-0.459086379	4.472826252	-5.117086853	1.58E-06	0.00036756	2.836226204
BASP1	0.336033956	2.786008194	3.651819305	0.000423036	0.007342131	-2.555898684
BCL9L	-0.275063697	2.999065748	-3.797136546	0.000255788	0.005461516	-2.077902137
CA4	0.284658408	0.959597915	4.723312752	7.87E-06	0.000781087	1.271980299
CD163	0.270252517	1.355742053	3.077805692	0.002714545	0.023797543	-4.300950711
CD177	0.540602136	0.740615859	3.226736033	0.001710387	0.017614371	-3.871005503
CD3D	-0.3456734	3.329349163	-3.735746635	0.000316848	0.006184622	-2.281535756
CD3E	-0.29566686	3.731669144	-3.445943055	0.000844108	0.011371645	-3.208693285
CD8A	-0.303790152	1.80620844	-2.964702068	0.003817057	0.029783018	-4.616299624
CDK5R1	0.290859691	1.782403593	3.121365112	0.002375127	0.021849025	-4.176912265
CEBPB	0.300237198	6.831641272	3.89289076	0.000182371	0.004351083	-1.755400168
CEBPD	0.395920236	7.173484023	4.023640366	0.000113933	0.003258191	-1.305640626
CLC	-0.50826088	2.67660985	-2.927023827	0.004267782	0.032243826	-4.719171046
CLEC4E	0.415214203	3.34992765	3.436648968	0.000870306	0.011564004	-3.237470419
CTSW	-0.558767352	4.192995068	-3.984422774	0.000131332	0.003535738	-1.441665099
CX3CR1	-0.457060791	2.846693807	-4.780474079	6.26E-06	0.000718879	1.494332454
DAAM2	0.337862008	0.246765572	3.616341418	0.000477404	0.007913816	-2.670459531
DGAT2	0.278505003	1.986450072	3.601158216	0.000502639	0.008182894	-2.719227186
DOK3	0.350765111	4.839952883	4.304804375	4.01E-05	0.001834362	-0.303214403
EOMES	-0.274082113	1.731368366	-3.472880589	0.00077231	0.010760775	-3.124944702
ESYT1	-0.284243357	2.90524577	-4.451185126	2.29E-05	0.001330658	0.236856551
FCRL6	-0.294763381	1.351002872	-3.787562902	0.00026451	0.00554144	-2.109820613
FGFBP2	-0.656502635	3.734140219	-4.546171871	1.59E-05	0.0010939	0.593672877
FKBP5	0.263159728	0.848389478	2.983329545	0.00361084	0.028764898	-4.565036832
FLOT2	0.28143406	4.171177588	4.06149598	9.92E-05	0.003027154	-1.173440695
FOLR3	0.34359562	1.95420764	2.254124595	0.026449737	0.111154885	-6.363934246
FPR1	0.279903253	6.521185239	3.372288047	0.001073848	0.013117322	-3.43507575
GATA2	-0.303462538	1.306612884	-3.594798082	0.00051358	0.008284306	-2.739609081
GBP4	-0.391892755	1.75692498	-3.832854885	0.000225603	0.005001311	-1.958292394
GBP5	-0.458176553	3.228473777	-3.210713984	0.001798793	0.01821096	-3.918049449
GCA	0.267861808	2.358376607	2.971570831	0.003739783	0.029401274	-4.597427894
GNLY	-0.565880765	5.43360921	-3.219802558	0.001748134	0.017856232	-3.891386742
GPR27	0.315167625	2.934512233	3.266990612	0.001505854	0.016307224	-3.751979695
GZMA	-0.426904921	2.584963178	-4.298090506	4.12E-05	0.001858081	-0.327693573
GZMB	-0.694264893	3.814946378	-4.984307037	2.73E-06	0.000501605	2.300438735
GZMH	-0.646560872	3.860774028	-3.751632068	0.000299838	0.006017821	-2.229079903
HAUS4	0.274408521	2.159998866	3.618399024	0.000474078	0.007895767	-2.663838584
HMGB2	0.315988135	4.791305617	3.538567116	0.00062062	0.009442901	-2.918606972
HRH2	0.263105659	1.428987275	3.433583203	0.000879115	0.011632456	-3.246949569

IFIT1	-0.475400466	1.625552775	-2.118958267	0.036661034	0.139960021	-6.648379503
IFITM2	0.275812562	9.958736752	3.680842992	0.00038298	0.006934785	-2.461549648
IL18R1	0.282933691	0.590776274	3.169767981	0.002044476	0.019755001	-4.037414767
IL18RAP	0.362491893	1.594988476	2.821668278	0.005800775	0.039636236	-5.000945261
IL1R2	0.629335381	1.548555622	4.204601465	5.85E-05	0.00228501	-0.665844967
IL2RB	-0.337020444	1.572884642	-5.098391545	1.71E-06	0.00037708	2.760294401
IRAK3	0.327633849	1.234002128	3.472045066	0.000774448	0.010784844	-3.127549968
KLRC4	-0.265471775	1.479562415	-3.01544993	0.003279254	0.026926339	-4.476015618
LILRA5	0.28748811	3.601472972	3.212133577	0.001790791	0.018171932	-3.913888867
LRG1	0.37055163	4.685314435	3.339090851	0.001195561	0.014014131	-3.535847746
LSMEM1	0.310411569	0.914127659	4.784957299	6.15E-06	0.000712504	1.511841746
LTB4R	0.296287459	3.439388362	5.006349792	2.50E-06	0.00047572	2.388814813
MANSC1	0.305538158	1.254099139	3.980820906	0.000133051	0.003567473	-1.454110165
MCEMP1	0.458653838	2.774151029	3.087138745	0.00263824	0.023355086	-4.274494454
MGAM	0.378116274	1.913971573	3.384086498	0.001033468	0.012835113	-3.399071306
MKNK1	0.274167009	1.409028112	3.905093029	0.000174609	0.004262602	-1.713881021
MMP9	0.618390196	4.271388021	3.989883359	0.000128765	0.003504651	-1.422782455
NCF4	0.286911462	3.878608944	4.568353056	1.45E-05	0.001039103	0.677700538
NKG7	-0.630839795	6.516766489	-4.463556653	2.19E-05	0.001294252	0.28305036
NQO2	0.301466366	1.829160684	3.82416425	0.000232618	0.00510255	-1.987470677
OAS2	-0.340311219	1.47348603	-2.72497431	0.007635751	0.048037327	-5.251831967
OAS3	-0.42536748	1.314013959	-2.412221852	0.017745403	0.084449092	-6.011234775
ORM1	0.463336336	1.725155266	2.893550267	0.004708806	0.034389983	-4.809638155
PADI4	0.355510831	1.753434081	4.421644576	2.57E-05	0.001432643	0.126898231
PFKFB2	0.272828563	0.586468578	3.337198382	0.001202876	0.014068509	-3.541568656
PGLYRP1	0.340095254	2.718012683	3.274135564	0.001472033	0.016105987	-3.730729702
PLD4	-0.296563825	1.011845142	-4.382783068	2.98E-05	0.001534395	-0.0170145
PLIN5	0.366669156	1.435904612	4.313566572	3.88E-05	0.001822496	-0.271227969
PRF1	-0.637092708	5.137282042	-4.965685282	2.95E-06	0.00051811	2.225958025
PROK2	0.34307606	2.817716509	2.935082786	0.004167444	0.03170441	-4.697260405
PTGDR2	-0.368943246	1.497144115	-3.290642752	0.001396594	0.015565597	-3.681493327
PYGL	0.339517328	2.749836187	3.973372364	0.000136676	0.003617964	-1.479820495
RUNX3	-0.283565454	1.66549065	-5.186829748	1.18E-06	0.000319578	3.120886852
S100A11	0.295302204	6.368151309	3.986068703	0.000130553	0.003527749	-1.435975447
S100A12	0.436158284	5.603418212	3.039148876	0.003052916	0.025710205	-4.409828603
S100A8	0.326205534	4.937382822	3.336777705	0.001204507	0.014075069	-3.542840008
S100A9	0.287125741	9.681387078	3.454187123	0.000821493	0.011185239	-3.183116528
S100P	0.401650727	3.983432168	2.935611065	0.004160943	0.031673284	-4.695822365
S1PR1	-0.322013152	3.65420141	-3.603572901	0.000498543	0.008126275	-2.711481806
S1PR5	-0.5112918	2.496533984	-4.923616991	3.51E-06	0.000549439	2.058309538
SCARNA20	0.275366376	1.411687142	2.856803556	0.005240931	0.037071444	-4.907943486
SH2D2A	-0.274901547	1.619595385	-3.880589658	0.000190528	0.004473615	-1.797159743
SLAMF7	-0.358693847	1.591090967	-5.201695857	1.11E-06	0.000317265	3.181845731
SLC11A1	0.533532819	4.676706918	4.622088336	1.18E-05	0.000945991	0.882351071
SLC2A3	0.265652795	3.60499521	3.033757972	0.003103092	0.026014642	-4.424922069
SLFN5	-0.31451395	1.942831787	-5.541538871	2.60E-07	0.000155238	4.601191083
SLPI	0.349991905	2.334834972	2.534804652	0.012858703	0.06783327	-5.723214541
SPN	-0.473245515	4.378996326	-5.672756318	1.47E-07	0.000116629	5.161760314
TAS2R40	0.286806705	0.766704794	3.482932536	0.000747025	0.010540919	-3.093563283
TBX21	-0.407093162	2.139645689	-4.56831646	1.45E-05	0.001039103	0.677561685

VNN1	0.263287987	0.641360098	3.184838467	0.001950624	0.019186607	-3.99362568
VNN2	0.333692561	4.332382872	3.178438015	0.001989981	0.019392359	-4.012243519
