# Supplementary material for: Integrated bioinformatics analysis of the effects of chronic pain on patients with spinal cord injury
Source: Front Cell Neurosci. 2025 Feb 5;19:1457740. doi: 10.3389/fncel.2025.1457740 (PMC11835904; doi:10.3389/fncel.2025.1457740)
Supplement: Supplementary Table S2-1 — Candidate items of GO analysis in GSE151371. [file Data_Sheet_3.pdf]

ONTOLOGY	ID	Description	GeneRatio	BgRatio	pvalue	p.adjust	qvalue	geneID	Count
BP	GO:0050832	defense response to fungus	7/96	49/18723	5.38E-09	1.01E-05	8.26E-06	CLEC4E/S100A9/ARG1/S100A8/S100A12/GNLY/CX3CR1	7
BP	GO:0009620	response to fungus	7/96	60/18723	2.31E-08	1.28E-05	1.05E-05	CLEC4E/S100A9/ARG1/S100A8/S100A12/GNLY/CX3CR1	7
BP	GO:0042110	T cell activation	15/96	487/18723	2.58E-08	1.28E-05	1.05E-05	FLOT2/IL18R1/VNN1/SLC11A1/CEBPB/ARG1/CLC/CD3D/CD8A/EOMES/SLAMF7/TBX21/SPN/RUNX3/CD3E	15
BP	GO:0042742	defense response to bacterium	13/96	350/18723	2.72E-08	1.28E-05	1.05E-05	PGLYRP1/SLC11A1/HMGB2/CEBPB/S100A9/SLPI/S100A8/S100A12/OAS3/GNLY/OAS2/SPN/GBP4	13
BP	GO:0007159	leukocyte cell-cell adhesion	13/96	371/18723	5.39E-08	2.03E-05	1.66E-05	FLOT2/ALOX5/VNN1/CD177/CEBPB/S100A9/ARG1/S100A8/CX3CR1/TBX21/SPN/RUNX3/CD3E	13
BP	GO:0001906	cell killing	9/96	188/18723	5.24E-07	0.000164208	0.000134164	PGLYRP1/IL18RAP/ARG1/S100A12/GNLY/GZMB/SLAMF7/PRF1/CX3CR1	9
BP	GO:0051092	positive regulation of NF-kappaB transcription factor activity	8/96	152/18723	1.14E-06	0.00030554	0.000249637	FLOT2/IL18R1/IL18RAP/S100A9/S100A8/S100A12/IRAK3/CX3CR1	8
BP	GO:0031349	positive regulation of defense response	10/96	278/18723	1.63E-06	0.000353604	0.000288907	ALOX5AP/IL18RAP/HMGB2/CEBPB/S100A9/LILRA5/ARG1/S100A8/S100A12/GBP5	10
BP	GO:1903131	mononuclear cell differentiation	12/96	426/18723	1.83E-06	0.000353604	0.000288907	ADGRG3/IL18R1/PGLYRP1/VNN1/CEBPB/CD3D/CD8A/EOMES/TBX21/SPN/RUNX3/CD3E	12
BP	GO:0032103	positive regulation of response to external stimulus	12/96	427/18723	1.88E-06	0.000353604	0.000288907	ALOX5AP/IL18RAP/HMGB2/CEBPB/S100A9/LILRA5/ARG1/S100A8/S100A12/GBP5/S1PR1/CX3CR1	12
BP	GO:0002443	leukocyte mediated immunity	12/96	440/18723	2.56E-06	0.000438315	0.000358118	IL18R1/IL18RAP/CD177/ARG1/CLC/GATA2/CD8A/GZMB/SLAMF7/PRF1/CX3CR1/TBX21	12
BP	GO:0030098	lymphocyte differentiation	11/96	374/18723	3.37E-06	0.000528405	0.000431725	ADGRG3/IL18R1/PGLYRP1/VNN1/CD3D/CD8A/EOMES/TBX21/SPN/RUNX3/CD3E	11
BP	GO:0032069	regulation of nuclease activity	4/96	22/18723	4.42E-06	0.000634024	0.00051802	HMGB2/OAS3/GZMA/OAS2	4
BP	GO:0001819	positive regulation of cytokine production	12/96	467/18723	4.72E-06	0.000634024	0.00051802	IL18R1/ORM1/SLC11A1/HMGB2/CEBPB/LILRA5/IRAK3/OAS3/GBP5/OAS2/SPN/CD3E	12
BP	GO:0019221	cytokine-mediated signaling pathway	12/96	472/18723	5.26E-06	0.000659479	0.000538817	IFITM2/IL18R1/IL1R2/IL18RAP/ACSL1/LILRA5/ARG1/IRAK3/OAS3/CX3CR1/OAS2/IL2RB	12
BP	GO:0030217	T cell differentiation	9/96	257/18723	6.89E-06	0.000810263	0.000662013	IL18R1/VNN1/CD3D/CD8A/EOMES/TBX21/SPN/RUNX3/CD3E	9
BP	GO:0002697	regulation of immune effector process	10/96	339/18723	9.42E-06	0.001032938	0.000843946	IL18R1/PGLYRP1/IL18RAP/CD177/ARG1/IRAK3/CLC/GATA2/CX3CR1/TBX21	10
BP	GO:0045071	negative regulation of viral genome replication	5/96	56/18723	9.91E-06	0.001032938	0.000843946	IFITM2/SLPI/IFIT1/OAS3/OAS2	5
BP	GO:0032496	response to lipopolysaccharide	10/96	343/18723	1.04E-05	0.001032938	0.000843946	SLC11A1/ALPL/HMGB2/CEBPB/S100A9/SLPI/ARG1/S100A8/IRAK3/CX3CR1	10
BP	GO:0002366	leukocyte activation involved in immune response	9/96	275/18723	1.19E-05	0.001115814	0.000911659	IL18R1/PGLYRP1/CD177/SLC11A1/GATA2/EOMES/CX3CR1/TBX21/SPN	9
BP	GO:0002263	cell activation involved in immune response	9/96	279/18723	1.33E-05	0.001175185	0.000960167	IL18R1/PGLYRP1/CD177/SLC11A1/GATA2/EOMES/CX3CR1/TBX21/SPN	9
BP	GO:0002683	negative regulation of immune system process	11/96	434/18723	1.37E-05	0.001175185	0.000960167	PGLYRP1/CEBPB/ARG1/IRAK3/GATA2/OAS3/ALOX15/CX3CR1/TBX21/SPN/RUNX3	11
BP	GO:0001818	negative regulation of cytokine production	10/96	357/18723	1.48E-05	0.001206649	0.000985874	ORM1/PGLYRP1/IL1R2/SLC11A1/LILRA5/ARG1/IRAK3/OAS3/CX3CR1/TBX21	10
BP	GO:0002237	response to molecule of bacterial origin	10/96	363/18723	1.70E-05	0.00133493	0.001090684	SLC11A1/ALPL/HMGB2/CEBPB/S100A9/SLPI/ARG1/S100A8/IRAK3/CX3CR1	10
BP	GO:0050900	leukocyte migration	10/96	369/18723	1.96E-05	0.001375407	0.001123755	ALOX5/MMP9/CD177/S100A9/S100A8/S100A12/S1PR1/CX3CR1/TBX21/SPN	10
BP	GO:0002456	T cell mediated immunity	6/96	109/18723	2.05E-05	0.001375407	0.001123755	IL18R1/ARG1/CLC/CD8A/PRF1/TBX21	6
BP	GO:0019835	cytolysis	4/96	32/18723	2.09E-05	0.001375407	0.001123755	GZMH/GZMA/GZMB/PRF1	4
BP	GO:0019372	lipoxygenase pathway	3/96	11/18723	2.09E-05	0.001375407	0.001123755	ALOX5AP/ALOX5/ALOX15	3
BP	GO:0002703	regulation of leukocyte mediated immunity	8/96	226/18723	2.12E-05	0.001375407	0.001123755	IL18R1/IL18RAP/CD177/ARG1/CLC/GATA2/CX3CR1/TBX21	8
BP	GO:0002526	acute inflammatory response	6/96	112/18723	2.40E-05	0.001502898	0.00122792	CD163/ORM1/ALOX5AP/VNN1/CEBPB/S100A8	6
BP	GO:0031640	killing of cells of other organism	5/96	68/18723	2.58E-05	0.001563126	0.001277128	PGLYRP1/ARG1/S100A12/GNLY/PRF1	5
BP	GO:0050727	regulation of inflammatory response	10/96	386/18723	2.88E-05	0.001692915	0.00138317	ALOX5AP/PGLYRP1/ALOX5/MMP9/CEBPB/S100A9/LILRA5/S100A8/S100A12/ALOX15	10
BP	GO:0002369	T cell cytokine production	4/96	37/18723	3.76E-05	0.002009048	0.001641461	IL18R1/ARG1/CLC/TBX21	4
BP	GO:0002724	regulation of T cell cytokine production	4/96	37/18723	3.76E-05	0.002009048	0.001641461	IL18R1/ARG1/CLC/TBX21	4
BP	GO:0043368	positive T cell selection	4/96	37/18723	3.76E-05	0.002009048	0.001641461	CD3D/TBX21/SPN/CD3E	4
BP	GO:0019730	antimicrobial humoral response	6/96	122/18723	3.89E-05	0.002009048	0.001641461	PGLYRP1/SLC11A1/S100A9/SLPI/S100A12/GNLY	6
BP	GO:0032640	tumor necrosis factor production	7/96	181/18723	4.06E-05	0.002009048	0.001641461	ORM1/LILRA5/IRAK3/OAS3/CX3CR1/OAS2/SPN	7
BP	GO:0032680	regulation of tumor necrosis factor production	7/96	181/18723	4.06E-05	0.002009048	0.001641461	ORM1/LILRA5/IRAK3/OAS3/CX3CR1/OAS2/SPN	7
BP	GO:0001909	leukocyte mediated cytotoxicity	6/96	124/18723	4.26E-05	0.002054004	0.001678192	IL18RAP/ARG1/GZMB/SLAMF7/PRF1/CX3CR1	6
BP	GO:0071706	tumor necrosis factor superfamily cytokine production	7/96	186/18723	4.83E-05	0.002174212	0.001776406	ORM1/LILRA5/IRAK3/OAS3/CX3CR1/OAS2/SPN	7
BP	GO:1903555	regulation of tumor necrosis factor superfamily cytokine production	7/96	186/18723	4.83E-05	0.002174212	0.001776406	ORM1/LILRA5/IRAK3/OAS3/CX3CR1/OAS2/SPN	7
BP	GO:0050863	regulation of T cell activation	9/96	329/18723	4.85E-05	0.002174212	0.001776406	FLOT2/VNN1/CEBPB/ARG1/CLC/TBX21/SPN/RUNX3/CD3E	9
BP	GO:1903037	regulation of leukocyte cell-cell adhesion	9/96	336/18723	5.71E-05	0.002453467	0.002004567	FLOT2/ALOX5/VNN1/CEBPB/ARG1/TBX21/SPN/RUNX3/CD3E	9
BP	GO:0051091	positive regulation of DNA-binding transcription factor activity	8/96	260/18723	5.74E-05	0.002453467	0.002004567	FLOT2/IL18R1/IL18RAP/S100A9/S100A8/S100A12/IRAK3/CX3CR1	8
BP	GO:0045069	regulation of viral genome replication	5/96	85/18723	7.57E-05	0.003163623	0.00258479	IFITM2/SLPI/IFIT1/OAS3/OAS2	5
BP	GO:0002449	lymphocyte mediated immunity	9/96	350/18723	7.81E-05	0.003193766	0.002609417	IL18R1/IL18RAP/ARG1/CLC/CD8A/GZMB/SLAMF7/PRF1/TBX21	9
BP	GO:0051090	regulation of DNA-binding transcription factor activity	10/96	440/18723	8.63E-05	0.003452561	0.002820862	FLOT2/ADGRG3/IL18R1/IL18RAP/S100A9/S100A8/S100A12/IRAK3/EOMES/CX3CR1	10
BP	GO:0050729	positive regulation of inflammatory response	6/96	142/18723	9.06E-05	0.003551178	0.002901435	ALOX5AP/CEBPB/S100A9/LILRA5/S100A8/S100A12	6
BP	GO:0022407	regulation of cell-cell adhesion	10/96	448/18723	0.000100072	0.003646357	0.0029792	FLOT2/ALOX5/VNN1/CEBPB/ARG1/ALOX15/TBX21/SPN/RUNX3/CD3E	10
BP	GO:0002544	chronic inflammatory response	3/96	18/18723	0.000100803	0.003646357	0.0029792	VNN1/S100A9/S100A8	3
BP	GO:0002643	regulation of tolerance induction	3/96	18/18723	0.000100803	0.003646357	0.0029792	IRAK3/CLC/CD3E	3
BP	GO:0035743	CD4-positive, alpha-beta T cell cytokine production	3/96	18/18723	0.000100803	0.003646357	0.0029792	IL18R1/ARG1/TBX21	3
BP	GO:0048525	negative regulation of viral process	5/96	92/18723	0.00011031	0.003914954	0.003198653	IFITM2/SLPI/IFIT1/OAS3/OAS2	5
BP	GO:0002523	leukocyte migration involved in inflammatory response	3/96	19/18723	0.000119259	0.00415419	0.003394117	ALOX5/S100A9/S100A8	3
BP	GO:0045058	T cell selection	4/96	50/18723	0.000124753	0.004266555	0.003485923	CD3D/TBX21/SPN/CD3E	4
BP	GO:0002532	production of molecular mediator involved in inflammatory response	5/96	95/18723	0.000128404	0.004313005	0.003523874	ALOX5AP/ALOX5/IL1R2/PLD4/GBP5	5
BP	GO:0002718	regulation of cytokine production involved in immune response	5/96	96/18723	0.000134914	0.004452172	0.003637579	IL18R1/ARG1/IRAK3/CLC/TBX21	5
BP	GO:0071357	cellular response to type I interferon	4/96	52/18723	0.000145507	0.004718939	0.003855536	IFITM2/IFIT1/OAS3/OAS2	4
BP	GO:0002367	cytokine production involved in immune response	5/96	98/18723	0.000148691	0.004740458	0.003873118	IL18R1/ARG1/IRAK3/CLC/TBX21	5
BP	GO:0046631	alpha-beta T cell activation	6/96	156/18723	0.000151886	0.004761612	0.003890402	IL18R1/EOMES/TBX21/SPN/RUNX3/CD3E	6
BP	GO:0051250	negative regulation of lymphocyte activation	6/96	157/18723	0.000157274	0.004849722	0.003962391	PGLYRP1/CEBPB/ARG1/TBX21/SPN/RUNX3	6
BP	GO:0006968	cellular defense response	4/96	54/18723	0.00016865	0.005116618	0.004180454	GNLY/PRF1/CX3CR1/SPN	4
BP	GO:0032760	positive regulation of tumor necrosis factor production	5/96	103/18723	0.000187848	0.005608592	0.004582414	ORM1/LILRA5/OAS3/OAS2/SPN	5
BP	GO:0002886	regulation of myeloid leukocyte mediated immunity	4/96	56/18723	0.00019434	0.005687604	0.004646969	CD177/ARG1/GATA2/CX3CR1	4
BP	GO:0007200	phospholipase C-activating G protein-coupled receptor signaling pathway	5/96	104/18723	0.000196541	0.005687604	0.004646969	FPR1/LTB4R/GPR27/S1PR1/CX3CR1	5
BP	GO:0035821	modulation of process of other organism	5/96	106/18723	0.000214847	0.006123128	0.005002807	S100A9/SLPI/ARG1/PRF1/CX3CR1	5
BP	GO:0034340	response to type I interferon	4/96	58/18723	0.000222739	0.006209299	0.005073212	IFITM2/IFIT1/OAS3/OAS2	4
BP	GO:1903557	positive regulation of tumor necrosis factor superfamily cytokine production	5/96	107/18723	0.000224472	0.006209299	0.005073212	ORM1/LILRA5/OAS3/OAS2/SPN	5
BP	GO:0002698	negative regulation of immune effector process	5/96	110/18723	0.000255328	0.006960463	0.005686939	PGLYRP1/ARG1/IRAK3/CX3CR1/TBX21	5
BP	GO:0042832	defense response to protozoan	3/96	25/18723	0.000276825	0.007355002	0.006009291	SLC11A1/ARG1/GBP4	3
BP	GO:0046632	alpha-beta T cell differentiation	5/96	112/18723	0.000277621	0.007355002	0.006009291	IL18R1/EOMES/TBX21/SPN/RUNX3	5
BP	GO:0002286	T cell activation involved in immune response	5/96	114/18723	0.000301362	0.007873082	0.006432581	IL18R1/SLC11A1/EOMES/TBX21/SPN	5
BP	GO:0001562	response to protozoan	3/96	26/18723	0.000311772	0.008033478	0.00656363	SLC11A1/ARG1/GBP4	3
BP	GO:0002719	negative regulation of cytokine production involved in immune response	3/96	27/18723	0.000349443	0.008882473	0.007257288	ARG1/IRAK3/TBX21	3
BP	GO:0002819	regulation of adaptive immune response	6/96	183/18723	0.000359513	0.009016574	0.007366853	IL18R1/SLC11A1/ARG1/CLC/ALOX15/TBX21	6
BP	GO:0002507	tolerance induction	3/96	28/18723	0.000389926	0.009650661	0.007884924	IRAK3/CLC/CD3E	3
BP	GO:0002695	negative regulation of leukocyte activation	6/96	187/18723	0.000403385	0.009854128	0.008051163	PGLYRP1/CEBPB/ARG1/TBX21/SPN/RUNX3	6
BP	GO:0050868	negative regulation of T cell activation	5/96	122/18723	0.000412046	0.00993664	0.008118579	CEBPB/ARG1/TBX21/SPN/RUNX3	5
BP	GO:0002287	alpha-beta T cell activation involved in immune response	4/96	69/18723	0.000434749	0.010222033	0.008351754	IL18R1/EOMES/TBX21/SPN	4
BP	GO:0002293	alpha-beta T cell differentiation involved in immune response	4/96	69/18723	0.000434749	0.010222033	0.008351754	IL18R1/EOMES/TBX21/SPN	4
BP	GO:0001935	endothelial cell proliferation	6/96	193/18723	0.000476926	0.010935439	0.008934632	ALOX5/LRG1/HMGB2/PROK2/ARG1/GATA2	6
BP	GO:0002460	adaptive immune response based on somatic recombination of immune receptors built from immunoglobulin superfamily domains	8/96	356/18723	0.000489189	0.010935439	0.008934632	IL18R1/SLC11A1/ARG1/CLC/CD8A/PRF1/TBX21/SPN	8
BP	GO:0002285	lymphocyte activation involved in immune response	6/96	194/18723	0.000490137	0.010935439	0.008934632	IL18R1/PGLYRP1/SLC11A1/EOMES/TBX21/SPN	6
BP	GO:0050777	negative regulation of immune response	6/96	194/18723	0.000490137	0.010935439	0.008934632	PGLYRP1/ARG1/IRAK3/OAS3/ALOX15/TBX21	6
BP	GO:0002534	cytokine production involved in inflammatory response	4/96	72/18723	0.00051134	0.010935439	0.008934632	ALOX5/IL1R2/PLD4/GBP5	4
BP	GO:1900015	regulation of cytokine production involved in inflammatory response	4/96	72/18723	0.00051134	0.010935439	0.008934632	ALOX5/IL1R2/PLD4/GBP5	4
BP	GO:0032612	interleukin-1 production	5/96	128/18723	0.000513365	0.010935439	0.008934632	ORM1/IL1R2/LILRA5/GBP5/CX3CR1	5
BP	GO:0032652	regulation of interleukin-1 production	5/96	128/18723	0.000513365	0.010935439	0.008934632	ORM1/IL1R2/LILRA5/GBP5/CX3CR1	5
BP	GO:0071674	mononuclear cell migration	6/96	196/18723	0.000517413	0.010935439	0.008934632	ALOX5/S100A12/S1PR1/CX3CR1/TBX21/SPN	6
BP	GO:0042098	T cell proliferation	6/96	199/18723	0.000560529	0.011665172	0.00953085	SLC11A1/CEBPB/ARG1/CLC/SPN/CD3E	6
BP	GO:1901568	fatty acid derivative metabolic process	4/96	74/18723	0.000567446	0.011665172	0.00953085	ALOX5/DGAT2/ACSL1/ALOX15	4
BP	GO:0019079	viral genome replication	5/96	131/18723	0.000570545	0.011665172	0.00953085	IFITM2/SLPI/IFIT1/OAS3/OAS2	5
BP	GO:0002292	T cell differentiation involved in immune response	4/96	75/18723	0.000597079	0.012076404	0.00986684	IL18R1/EOMES/TBX21/SPN	4
BP	GO:0002705	positive regulation of leukocyte mediated immunity	5/96	134/18723	0.000632384	0.012654401	0.010339083	IL18R1/IL18RAP/CD177/ARG1/TBX21	5
BP	GO:0061844	antimicrobial humoral immune response mediated by antimicrobial peptide	4/96	79/18723	0.000726655	0.014387776	0.011755311	PGLYRP1/S100A9/S100A12/GNLY	4
BP	GO:0050866	negative regulation of cell activation	6/96	210/18723	0.00074302	0.014558546	0.011894836	PGLYRP1/CEBPB/ARG1/TBX21/SPN/RUNX3	6
BP	GO:0034341	response to interferon-gamma	5/96	141/18723	0.00079613	0.015169234	0.012393789	IFITM2/SLC11A1/ARG1/GBP5/GBP4	5
BP	GO:1903038	negative regulation of leukocyte cell-cell adhesion	5/96	141/18723	0.00079613	0.015169234	0.012393789	CEBPB/ARG1/TBX21/SPN/RUNX3	5
BP	GO:0001960	negative regulation of cytokine-mediated signaling pathway	4/96	81/18723	0.000798381	0.015169234	0.012393789	IL1R2/ARG1/IRAK3/OAS3	4
BP	GO:0043367	CD4-positive, alpha-beta T cell differentiation	4/96	83/18723	0.000874968	0.016458145	0.013446874	IL18R1/TBX21/SPN/RUNX3	4
BP	GO:0045088	regulation of innate immune response	6/96	218/18723	0.000902356	0.016805263	0.013730482	IL18RAP/HMGB2/ARG1/IRAK3/OAS3/GBP5	6
BP	GO:0002709	regulation of T cell mediated immunity	4/96	85/18723	0.000956595	0.017640733	0.014413089	IL18R1/ARG1/CLC/TBX21	4
BP	GO:1903900	regulation of viral life cycle	5/96	148/18723	0.000989644	0.01807302	0.014766283	IFITM2/SLPI/IFIT1/OAS3/OAS2	5
BP	GO:0060761	negative regulation of response to cytokine stimulus	4/96	86/18723	0.000999354	0.018074852	0.01476778	IL1R2/ARG1/IRAK3/OAS3	4
BP	GO:0002274	myeloid leukocyte activation	6/96	223/18723	0.001014555	0.018175021	0.014849621	IL18RAP/CD177/SLC11A1/S100A12/GATA2/CX3CR1	6
BP	GO:0002701	negative regulation of production of molecular mediator of immune response	3/96	39/18723	0.001044279	0.018357831	0.014998984	ARG1/IRAK3/TBX21	3
BP	GO:0032728	positive regulation of interferon-beta production	3/96	39/18723	0.001044279	0.018357831	0.014998984	HMGB2/OAS3/OAS2	3
BP	GO:0060326	cell chemotaxis	7/96	310/18723	0.001080035	0.018810612	0.015368921	ALOX5/HMGB2/S100A9/S100A8/S100A12/S1PR1/CX3CR1	7
BP	GO:1902023	L-arginine transport	2/96	10/18723	0.001139845	0.019670163	0.016071204	SLC11A1/ARG1	2
BP	GO:0030595	leukocyte chemotaxis	6/96	230/18723	0.001189317	0.020337329	0.016616302	ALOX5/S100A9/S100A8/S100A12/S1PR1/CX3CR1	6
BP	GO:0006959	humoral immune response	7/96	317/18723	0.001228617	0.020820085	0.01701073	PGLYRP1/ALOX5/SLC11A1/S100A9/SLPI/S100A12/GNLY	7
BP	GO:0019432	triglyceride biosynthetic process	3/96	42/18723	0.001297281	0.021787373	0.017801038	PLIN5/DGAT2/ACSL1	3
BP	GO:0002699	positive regulation of immune effector process	6/96	235/18723	0.001327647	0.022100037	0.018056495	IL18R1/IL18RAP/CD177/ARG1/GATA2/TBX21	6
BP	GO:0002645	positive regulation of tolerance induction	2/96	11/18723	0.001388494	0.022337662	0.018250643	IRAK3/CD3E	2
BP	GO:0002725	negative regulation of T cell cytokine production	2/96	11/18723	0.001388494	0.022337662	0.018250643	ARG1/TBX21	2
BP	GO:0045654	positive regulation of megakaryocyte differentiation	2/96	11/18723	0.001388494	0.022337662	0.018250643	HMGB2/GATA2	2
BP	GO:0042088	T-helper 1 type immune response	3/96	43/18723	0.001389424	0.022337662	0.018250643	IL18R1/SLC11A1/SPN	3
BP	GO:0050867	positive regulation of cell activation	8/96	420/18723	0.001423465	0.022690991	0.018539325	FLOT2/VNN1/CD177/LILRA5/GATA2/TBX21/RUNX3/CD3E	8
BP	GO:0050764	regulation of phagocytosis	4/96	95/18723	0.001446563	0.022865428	0.018681846	SLC11A1/GATA2/ALOX15/IL2RB	4
BP	GO:0002831	regulation of response to biotic stimulus	7/96	327/18723	0.001468207	0.023014137	0.018803347	IL18RAP/HMGB2/ARG1/IRAK3/IFIT1/OAS3/GBP5	7

BP	GO:0002700	regulation of production of molecular mediator of immune response	5/96	164/18723	0.001561131	0.024069567	0.019665669	IL18R1/ARG1/IRAK3/CLC/TBX21	5
BP	GO:0050792	regulation of viral process	5/96	164/18723	0.001561131	0.024069567	0.019665669	IFITM2/SLPI/IFIT1/OAS3/OAS2	5
BP	GO:0002664	regulation of T cell tolerance induction	2/96	12/18723	0.001660635	0.025140576	0.020540721	CLC/CD3E	2
BP	GO:0046322	negative regulation of fatty acid oxidation	2/96	12/18723	0.001660635	0.025140576	0.020540721	PLIN5/DGAT2	2
BP	GO:0002444	myeloid leukocyte mediated immunity	4/96	99/18723	0.001684058	0.025140576	0.020540721	CD177/ARG1/GATA2/CX3CR1	4
BP	GO:0031341	regulation of cell killing	4/96	99/18723	0.001684058	0.025140576	0.020540721	IL18RAP/ARG1/PRF1/CX3CR1	4
BP	GO:0002706	regulation of lymphocyte mediated immunity	5/96	168/18723	0.001735592	0.02550506	0.020838517	IL18R1/IL18RAP/ARG1/CLC/TBX21	5
BP	GO:0002822	regulation of adaptive immune response based on somatic recombination of immune receptors built from immunoglobulin superfamily domains	5/96	168/18723	0.001735592	0.02550506	0.020838517	IL18R1/SLC11A1/ARG1/CLC/TBX21	5
BP	GO:0031214	biomineral tissue development	5/96	169/18723	0.001781355	0.025974644	0.021222183	ALOX5/ALPL/CEBPB/ALOX15/S1PR1	5
BP	GO:0050673	epithelial cell proliferation	8/96	437/18723	0.001826489	0.026376918	0.021550855	ALOX5/LRG1/HMGB2/CEBPB/PROK2/ARG1/GATA2/RUNX3	8
BP	GO:0042129	regulation of T cell proliferation	5/96	171/18723	0.001875531	0.026376918	0.021550855	CEBPB/ARG1/CLC/SPN/CD3E	5
BP	GO:0110148	biomineralization	5/96	171/18723	0.001875531	0.026376918	0.021550855	ALOX5/ALPL/CEBPB/ALOX15/S1PR1	5
BP	GO:0035710	CD4-positive, alpha-beta T cell activation	4/96	102/18723	0.001879057	0.026376918	0.021550855	IL18R1/TBX21/SPN/RUNX3	4
BP	GO:1902106	negative regulation of leukocyte differentiation	4/96	102/18723	0.001879057	0.026376918	0.021550855	PGLYRP1/GATA2/TBX21/RUNX3	4
BP	GO:0015809	arginine transport	2/96	13/18723	0.001956024	0.026469648	0.021626618	SLC11A1/ARG1	2
BP	GO:0035745	T-helper 2 cell cytokine production	2/96	13/18723	0.001956024	0.026469648	0.021626618	ARG1/TBX21	2
BP	GO:0051709	regulation of killing of cells of other organism	2/96	13/18723	0.001956024	0.026469648	0.021626618	ARG1/PRF1	2
BP	GO:0070486	leukocyte aggregation	2/96	13/18723	0.001956024	0.026469648	0.021626618	S100A9/S100A8	2
BP	GO:2000551	regulation of T-helper 2 cell cytokine production	2/96	13/18723	0.001956024	0.026469648	0.021626618	ARG1/TBX21	2
BP	GO:0006953	acute-phase response	3/96	49/18723	0.002028752	0.027257737	0.022270514	CD163/ORM1/CEBPB	3
BP	GO:0046460	neutral lipid biosynthetic process	3/96	50/18723	0.002150284	0.028055233	0.022922097	PLIN5/DGAT2/ACSL1	3
BP	GO:0046463	acylglycerol biosynthetic process	3/96	50/18723	0.002150284	0.028055233	0.022922097	PLIN5/DGAT2/ACSL1	3
BP	GO:0060337	type I interferon signaling pathway	3/96	50/18723	0.002150284	0.028055233	0.022922097	IFITM2/OAS3/OAS2	3
BP	GO:1901570	fatty acid derivative biosynthetic process	3/96	50/18723	0.002150284	0.028055233	0.022922097	ALOX5/ACSL1/ALOX15	3
BP	GO:1903707	negative regulation of hemopoiesis	4/96	106/18723	0.002162684	0.028055233	0.022922097	PGLYRP1/GATA2/TBX21/RUNX3	4
BP	GO:0002517	T cell tolerance induction	2/96	14/18723	0.002274421	0.028326861	0.023144026	CLC/CD3E	2
BP	GO:0002829	negative regulation of type 2 immune response	2/96	14/18723	0.002274421	0.028326861	0.023144026	ARG1/TBX21	2
BP	GO:0003376	sphingosine-1-phosphate receptor signaling pathway	2/96	14/18723	0.002274421	0.028326861	0.023144026	S1PR5/S1PR1	2
BP	GO:0010867	positive regulation of triglyceride biosynthetic process	2/96	14/18723	0.002274421	0.028326861	0.023144026	PLIN5/DGAT2	2
BP	GO:0043374	CD8-positive, alpha-beta T cell differentiation	2/96	14/18723	0.002274421	0.028326861	0.023144026	EOMES/RUNX3	2
BP	GO:0045059	positive thymic T cell selection	2/96	14/18723	0.002274421	0.028326861	0.023144026	CD3D/CD3E	2
BP	GO:0001936	regulation of endothelial cell proliferation	5/96	179/18723	0.002289039	0.028326861	0.023144026	ALOX5/LRG1/HMGB2/ARG1/GATA2	5
BP	GO:0034440	lipid oxidation	4/96	108/18723	0.002315013	0.028461047	0.02325366	PLIN5/ALOX5/DGAT2/ALOX15	4
BP	GO:0032611	interleukin-1 beta production	4/96	110/18723	0.002474582	0.030030255	0.024535758	ORM1/LILRA5/GBP5/CX3CR1	4
BP	GO:0032651	regulation of interleukin-1 beta production	4/96	110/18723	0.002474582	0.030030255	0.024535758	ORM1/LILRA5/GBP5/CX3CR1	4
BP	GO:0001938	positive regulation of endothelial cell proliferation	4/96	111/18723	0.002557135	0.030833149	0.02519175	LRG1/HMGB2/ARG1/GATA2	4
BP	GO:0035358	regulation of peroxisome proliferator activated receptor signaling pathway	2/96	15/18723	0.002615588	0.031054858	0.025372894	PLIN5/ALOX15	2
BP	GO:0060099	regulation of phagocytosis, engulfment	2/96	15/18723	0.002615588	0.031054858	0.025372894	GATA2/ALOX15	2
BP	GO:0032609	interferon-gamma production	4/96	112/18723	0.002641562	0.031054858	0.025372894	IL18R1/PGLYRP1/SLC11A1/CD3E	4
BP	GO:0032649	regulation of interferon-gamma production	4/96	112/18723	0.002641562	0.031054858	0.025372894	IL18R1/PGLYRP1/SLC11A1/CD3E	4
BP	GO:0009615	response to virus	7/96	367/18723	0.002811947	0.032606247	0.026640432	IFITM2/IRAK3/IFIT1/OAS3/PRF1/OAS2/TBX21	7
BP	GO:1903706	regulation of hemopoiesis	7/96	367/18723	0.002811947	0.032606247	0.026640432	PGLYRP1/VNN1/HMGB2/CEBPB/GATA2/TBX21/RUNX3	7
BP	GO:0045620	negative regulation of lymphocyte differentiation	3/96	55/18723	0.002825528	0.032606247	0.026640432	PGLYRP1/TBX21/RUNX3	3
BP	GO:0032608	interferon-beta production	3/96	56/18723	0.002974463	0.032772152	0.026775982	HMGB2/OAS3/OAS2	3
BP	GO:0032648	regulation of interferon-beta production	3/96	56/18723	0.002974463	0.032772152	0.026775982	HMGB2/OAS3/OAS2	3
BP	GO:0002827	positive regulation of T-helper 1 type immune response	2/96	16/18723	0.002979287	0.032772152	0.026775982	IL18R1/SLC11A1	2
BP	GO:0017014	protein nitrosylation	2/96	16/18723	0.002979287	0.032772152	0.026775982	S100A9/S100A8	2
BP	GO:0018119	peptidyl-cysteine S-nitrosylation	2/96	16/18723	0.002979287	0.032772152	0.026775982	S100A9/S100A8	2
BP	GO:0051238	sequestering of metal ion	2/96	16/18723	0.002979287	0.032772152	0.026775982	S100A9/S100A8	2
BP	GO:0090520	sphingolipid mediated signaling pathway	2/96	16/18723	0.002979287	0.032772152	0.026775982	S1PR5/S1PR1	2
BP	GO:1905153	regulation of membrane invagination	2/96	16/18723	0.002979287	0.032772152	0.026775982	GATA2/ALOX15	2
BP	GO:0061756	leukocyte adhesion to vascular endothelial cell	3/96	57/18723	0.003128145	0.0341322	0.027887189	ALOX5/CX3CR1/SPN	3
BP	GO:1902105	regulation of leukocyte differentiation	6/96	279/18723	0.003139219	0.0341322	0.027887189	PGLYRP1/VNN1/CEBPB/GATA2/TBX21/RUNX3	6
BP	GO:0032481	positive regulation of type I interferon production	3/96	58/18723	0.003286622	0.035529513	0.029028842	HMGB2/OAS3/OAS2	3
BP	GO:0009251	glucan catabolic process	2/96	17/18723	0.003365283	0.036154164	0.029539203	MGAM/PYGL	2
BP	GO:0022408	negative regulation of cell-cell adhesion	5/96	196/18723	0.003382846	0.036154164	0.029539203	CEBPB/ARG1/TBX21/SPN/RUNX3	5
BP	GO:0022409	positive regulation of cell-cell adhesion	6/96	284/18723	0.003425177	0.036253284	0.029620188	FLOT2/ALOX5/VNN1/ALOX15/RUNX3/CD3E	6
BP	GO:0002820	negative regulation of adaptive immune response	3/96	59/18723	0.00344994	0.036253284	0.029620188	ARG1/ALOX15/TBX21	3
BP	GO:0006767	water-soluble vitamin metabolic process	3/96	59/18723	0.00344994	0.036253284	0.029620188	VNN1/VNN2/SLC2A3	3
BP	GO:0072503	cellular divalent inorganic cation homeostasis	8/96	486/18723	0.003509238	0.036671537	0.029961915	SLC11A1/FPR1/S100A9/PROK2/S100A8/GATA2/S1PR1/CX3CR1	8
BP	GO:1990266	neutrophil migration	4/96	122/18723	0.003593479	0.037344384	0.030511654	CD177/S100A9/S100A8/S100A12	4
BP	GO:0046651	lymphocyte proliferation	6/96	288/18723	0.003667591	0.037905158	0.030969826	SLC11A1/CEBPB/ARG1/CLC/SPN/CD3E	6
BP	GO:0002295	T-helper cell lineage commitment	2/96	18/18723	0.003773343	0.038574225	0.031516477	TBX21/SPN	2
BP	GO:0044247	cellular polysaccharide catabolic process	2/96	18/18723	0.003773343	0.038574225	0.031516477	MGAM/PYGL	2
BP	GO:0032943	mononuclear cell proliferation	6/96	291/18723	0.003857616	0.039222574	0.032046201	SLC11A1/CEBPB/ARG1/CLC/SPN/CD3E	6
BP	GO:0032731	positive regulation of interleukin-1 beta production	3/96	62/18723	0.003969404	0.040142202	0.032797569	ORM1/LILRA5/GBP5	3
BP	GO:0002704	negative regulation of leukocyte mediated immunity	3/96	63/18723	0.004152541	0.041178565	0.033644313	ARG1/CX3CR1/TBX21	3
BP	GO:0031343	positive regulation of cell killing	3/96	63/18723	0.004152541	0.041178565	0.033644313	IL18RAP/ARG1/PRF1	3
BP	GO:0002888	positive regulation of myeloid leukocyte mediated immunity	2/96	19/18723	0.004203235	0.041178565	0.033644313	CD177/ARG1	2
BP	GO:0015802	basic amino acid transport	2/96	19/18723	0.004203235	0.041178565	0.033644313	SLC11A1/ARG1	2
BP	GO:0032695	negative regulation of interleukin-12 production	2/96	19/18723	0.004203235	0.041178565	0.033644313	LILRA5/IRAK3	2
BP	GO:0061760	antifungal innate immune response	2/96	19/18723	0.004203235	0.041178565	0.033644313	CLEC4E/CX3CR1	2
BP	GO:0000272	polysaccharide catabolic process	2/96	20/18723	0.004654729	0.044900235	0.036685046	MGAM/PYGL	2
BP	GO:0043651	linoleic acid metabolic process	2/96	20/18723	0.004654729	0.044900235	0.036685046	ALOX5/ALOX15	2
BP	GO:0045063	T-helper 1 cell differentiation	2/96	20/18723	0.004654729	0.044900235	0.036685046	IL18R1/SPN	2
BP	GO:0042093	T-helper cell differentiation	3/96	66/18723	0.004732486	0.045186832	0.036919206	IL18R1/TBX21/SPN	3
BP	GO:0050766	positive regulation of phagocytosis	3/96	66/18723	0.004732486	0.045186832	0.036919206	SLC11A1/GATA2/IL2RB	3
BP	GO:0042130	negative regulation of T cell proliferation	3/96	67/18723	0.004936111	0.046893056	0.03831325	CEBPB/ARG1/SPN	3
BP	GO:0001503	ossification	7/96	408/18723	0.005013574	0.046978939	0.038383419	ALOX5/MMP9/ALPL/CEBPB/ALOX15/S1PR1/RUNX3	7
BP	GO:0006909	phagocytosis	6/96	308/18723	0.005075718	0.046978939	0.038383419	NCF4/SLC11A1/GATA2/PLD4/ALOX15/IL2RB	6
BP	GO:0002696	positive regulation of leukocyte activation	7/96	409/18723	0.005079928	0.046978939	0.038383419	FLOT2/VNN1/CD177/GATA2/TBX21/RUNX3/CD3E	7
BP	GO:0019370	leukotriene biosynthetic process	2/96	21/18723	0.005127598	0.046978939	0.038383419	ALOX5AP/ALOX5	2
BP	GO:0043373	CD4-positive, alpha-beta T cell lineage commitment	2/96	21/18723	0.005127598	0.046978939	0.038383419	TBX21/SPN	2
BP	GO:0070269	pyroptosis	2/96	21/18723	0.005127598	0.046978939	0.038383419	GZMA/GZMB	2
BP	GO:0002294	CD4-positive, alpha-beta T cell differentiation involved in immune response	3/96	68/18723	0.005144956	0.046978939	0.038383419	IL18R1/TBX21/SPN	3
BP	GO:0042267	natural killer cell mediated cytotoxicity	3/96	68/18723	0.005144956	0.046978939	0.038383419	IL18RAP/GZMB/SLAMF7	3
BP	GO:0097529	myeloid leukocyte migration	5/96	220/18723	0.005507677	0.049644401	0.040561194	CD177/S100A9/S100A8/S100A12/CX3CR1	5
BP	GO:0045123	cellular extravasation	3/96	70/18723	0.005578448	0.049644401	0.040561194	CD177/CX3CR1/SPN	3
BP	GO:0010866	regulation of triglyceride biosynthetic process	2/96	22/18723	0.005621615	0.049644401	0.040561194	PLIN5/DGAT2	2
BP	GO:0035357	peroxisome proliferator activated receptor signaling pathway	2/96	22/18723	0.005621615	0.049644401	0.040561194	PLIN5/ALOX15	2
BP	GO:0043371	negative regulation of CD4-positive, alpha-beta T cell differentiation	2/96	22/18723	0.005621615	0.049644401	0.040561194	TBX21/RUNX3	2
BP	GO:0045061	thymic T cell selection	2/96	22/18723	0.005621615	0.049644401	0.040561194	CD3D/CD3E	2
BP	GO:0061042	vascular wound healing	2/96	22/18723	0.005621615	0.049644401	0.040561194	ALOX5/GATA2	2
BP	GO:0002228	natural killer cell mediated immunity	3/96	71/18723	0.005803167	0.050770961	0.041481633	IL18RAP/GZMB/SLAMF7	3
BP	GO:0045824	negative regulation of innate immune response	3/96	71/18723	0.005803167	0.050770961	0.041481633	ARG1/IRAK3/OAS3	3
BP	GO:0070661	leukocyte proliferation	6/96	318/18723	0.00591272	0.051489933	0.042069057	SLC11A1/CEBPB/ARG1/CLC/SPN/CD3E	6
BP	GO:0032729	positive regulation of interferon-gamma production	3/96	72/18723	0.006033246	0.052178374	0.042631538	IL18R1/SLC11A1/CD3E	3
BP	GO:0050670	regulation of lymphocyte proliferation	5/96	225/18723	0.006047254	0.052178374	0.042631538	CEBPB/ARG1/CLC/SPN/CD3E	5
BP	GO:0002363	alpha-beta T cell lineage commitment	2/96	23/18723	0.006136555	0.052230133	0.042673827	TBX21/SPN	2
BP	GO:0043369	CD4-positive or CD8-positive, alpha-beta T cell lineage commitment	2/96	23/18723	0.006136555	0.052230133	0.042673827	TBX21/SPN	2
BP	GO:0090208	positive regulation of triglyceride metabolic process	2/96	23/18723	0.006136555	0.052230133	0.042673827	PLIN5/DGAT2	2
BP	GO:0032732	positive regulation of interleukin-1 production	3/96	73/18723	0.006268719	0.052914026	0.043232591	ORM1/LILRA5/GBP5	3
BP	GO:0032944	regulation of mononuclear cell proliferation	5/96	227/18723	0.006273167	0.052914026	0.043232591	CEBPB/ARG1/CLC/SPN/CD3E	5
BP	GO:0043302	positive regulation of leukocyte degranulation	2/96	24/18723	0.006672195	0.056028565	0.045777277	CD177/GATA2	2
BP	GO:0033077	T cell differentiation in thymus	3/96	75/18723	0.006755974	0.056479942	0.046146068	VNN1/CD3D/CD3E	3
BP	GO:0007188	adenylate cyclase-modulating G protein-coupled receptor signaling pathway	5/96	233/18723	0.006986495	0.058148659	0.047509467	ADGRG3/FPR1/PTGDR2/S1PR5/S1PR1	5
BP	GO:0097530	granulocyte migration	4/96	148/18723	0.007099413	0.058828175	0.048064655	CD177/S100A9/S100A8/S100A12	4
BP	GO:0032753	positive regulation of interleukin-4 production	2/96	25/18723	0.007228314	0.059373181	0.048509944	CEBPB/CD3E	2
BP	GO:0035336	long-chain fatty-acyl-CoA metabolic process	2/96	25/18723	0.007228314	0.059373181	0.048509944	DGAT2/ACSL1	2
BP	GO:0001959	regulation of cytokine-mediated signaling pathway	4/96	150/18723	0.007437432	0.060825262	0.049696345	IL1R2/ARG1/IRAK3/OAS3	4
BP	GO:0072593	reactive oxygen species metabolic process	5/96	239/18723	0.007754835	0.06247075	0.051040765	PLIN5/ALOX5/NCF4/NQO2/CD177	5
BP	GO:1903039	positive regulation of leukocyte cell-cell adhesion	5/96	239/18723	0.007754835	0.06247075	0.051040765	FLOT2/ALOX5/VNN1/RUNX3/CD3E	5
BP	GO:0002710	negative regulation of T cell mediated immunity	2/96	26/18723	0.007804692	0.06247075	0.051040765	ARG1/TBX21	2
BP	GO:0045745	positive regulation of G protein-coupled receptor signaling pathway	2/96	26/18723	0.007804692	0.06247075	0.051040765	GPR27/PTGDR2	2
BP	GO:0046639	negative regulation of alpha-beta T cell differentiation	2/96	26/18723	0.007804692	0.06247075	0.051040765	TBX21/RUNX3	2
BP	GO:0002825	regulation of T-helper 1 type immune response	2/96	27/18723	0.008401112	0.066677182	0.054477566	IL18R1/SLC11A1	2
BP	GO:0036037	CD8-positive, alpha-beta T cell activation	2/96	27/18723	0.008401112	0.066677182	0.054477566	EOMES/RUNX3	2
BP	GO:0070663	regulation of leukocyte proliferation	5/96	245/18723	0.008580248	0.067682219	0.055298716	CEBPB/ARG1/CLC/SPN/CD3E	5
BP	GO:0001892	embryonic placenta development	3/96	82/18723	0.00863569	0.067682219	0.055298716	CEBPB/GATA2/EOMES	3
BP	GO:0001910	regulation of leukocyte mediated cytotoxicity	3/96	82/18723	0.00863569	0.067682219	0.055298716	IL18RAP/ARG1/CX3CR1	3
BP	GO:2000377	regulation of reactive oxygen species metabolic process	4/96	157/18723	0.008703717	0.067932333	0.055503067	PLIN5/ALOX5/NQO2/CD177	4
BP	GO:0050672	negative regulation of lymphocyte proliferation	3/96	83/18723	0.008926717	0.069384936	0.056689894	CEBPB/ARG1/SPN	3
BP	GO:0032945	negative regulation of mononuclear cell proliferation	3/96	84/18723	0.009223442	0.071396273	0.058333226	CEBPB/ARG1/SPN	3

BP	GO:0002360	T cell lineage commitment	2/96	29/18723	0.009653213	0.074090555	0.060534548	TBX21/SPN	2
BP	GO:0042759	long-chain fatty acid biosynthetic process	2/96	29/18723	0.009653213	0.074090555	0.060534548	ALOX5/ALOX15	2
BP	GO:0060759	regulation of response to cytokine stimulus	4/96	162/18723	0.009689674	0.074090555	0.060534548	IL1R2/ARG1/IRAK3/OAS3	4
BP	GO:2001233	regulation of apoptotic signaling pathway	6/96	356/18723	0.01002797	0.076366847	0.062394357	VNN1/MMP9/HMGB2/S100A9/S100A8/CX3CR1	6
BP	GO:2001242	regulation of intrinsic apoptotic signaling pathway	4/96	164/18723	0.010103562	0.076632258	0.062611207	VNN1/MMP9/S100A9/S100A8	4
BP	GO:0071559	response to transforming growth factor beta	5/96	256/18723	0.010248396	0.076992197	0.06290529	LRG1/ARG1/BCL9L/CX3CR1/RUNX3	5
BP	GO:0070498	interleukin-1-mediated signaling pathway	2/96	30/18723	0.010308467	0.076992197	0.06290529	IL1R2/IRAK3	2
BP	GO:0032635	interleukin-6 production	4/96	165/18723	0.010314744	0.076992197	0.06290529	ORM1/CEBPB/LILRA5/IRAK3	4
BP	GO:0032675	regulation of interleukin-6 production	4/96	165/18723	0.010314744	0.076992197	0.06290529	ORM1/CEBPB/LILRA5/IRAK3	4
BP	GO:0030101	natural killer cell activation	3/96	88/18723	0.010467762	0.077825532	0.063586154	IL18R1/PGLYRP1/SLAMF7	3
BP	GO:0031348	negative regulation of defense response	5/96	258/18723	0.010573977	0.078305713	0.063978478	PGLYRP1/ALOX5/ARG1/IRAK3/OAS3	5
BP	GO:0002833	positive regulation of response to biotic stimulus	4/96	168/18723	0.010965414	0.080384617	0.065677014	IL18RAP/HMGB2/ARG1/GBP5	4
BP	GO:0002828	regulation of type 2 immune response	2/96	31/18723	0.010982906	0.080384617	0.065677014	ARG1/TBX21	2
BP	GO:0060055	angiogenesis involved in wound healing	2/96	31/18723	0.010982906	0.080384617	0.065677014	ALOX5/GATA2	2
BP	GO:0070664	negative regulation of leukocyte proliferation	3/96	90/18723	0.011124659	0.081106523	0.066266836	CEBPB/ARG1/SPN	3
BP	GO:0002275	myeloid cell activation involved in immune response	3/96	91/18723	0.011461857	0.083242292	0.068011834	CD177/GATA2/CX3CR1	3
BP	GO:2000515	negative regulation of CD4-positive, alpha-beta T cell activation	2/96	32/18723	0.011676322	0.084473702	0.069017938	TBX21/RUNX3	2
BP	GO:0051607	defense response to virus	5/96	265/18723	0.011769343	0.084496694	0.069036723	IFITM2/IFIT1/OAS3/PRF1/OAS2	5
BP	GO:0140546	defense response to symbiont	5/96	265/18723	0.011769343	0.084496694	0.069036723	IFITM2/IFIT1/OAS3/PRF1/OAS2	5
BP	GO:0007584	response to nutrient	4/96	174/18723	0.012345039	0.086627442	0.070777619	ACSL1/ALPL/ARG1/CD3E	4
BP	GO:0045619	regulation of lymphocyte differentiation	4/96	174/18723	0.012345039	0.086627442	0.070777619	PGLYRP1/VNN1/TBX21/RUNX3	4
BP	GO:0032633	interleukin-4 production	2/96	33/18723	0.012388507	0.086627442	0.070777619	CEBPB/CD3E	2
BP	GO:0032673	regulation of interleukin-4 production	2/96	33/18723	0.012388507	0.086627442	0.070777619	CEBPB/CD3E	2
BP	GO:0045648	positive regulation of erythrocyte differentiation	2/96	33/18723	0.012388507	0.086627442	0.070777619	HMGB2/GATA2	2
BP	GO:0046320	regulation of fatty acid oxidation	2/96	33/18723	0.012388507	0.086627442	0.070777619	PLIN5/DGAT2	2
BP	GO:0050901	leukocyte tethering or rolling	2/96	33/18723	0.012388507	0.086627442	0.070777619	CX3CR1/SPN	2
BP	GO:0032479	regulation of type I interferon production	3/96	95/18723	0.012869378	0.089325831	0.072982296	HMGB2/OAS3/OAS2	3
BP	GO:0032606	type I interferon production	3/96	95/18723	0.012869378	0.089325831	0.072982296	HMGB2/OAS3/OAS2	3
BP	GO:0002446	neutrophil mediated immunity	2/96	34/18723	0.013119254	0.090725428	0.074125815	CD177/ARG1	2
BP	GO:0070167	regulation of biomineral tissue development	3/96	97/18723	0.013608586	0.093754026	0.076600284	ALOX5/CEBPB/S1PR1	3
BP	GO:0050678	regulation of epithelial cell proliferation	6/96	381/18723	0.013656886	0.093754026	0.076600284	ALOX5/LRG1/HMGB2/ARG1/GATA2/RUNX3	6
BP	GO:0006691	leukotriene metabolic process	2/96	35/18723	0.013868358	0.094515871	0.077222738	ALOX5AP/ALOX5	2
BP	GO:0045922	negative regulation of fatty acid metabolic process	2/96	35/18723	0.013868358	0.094515871	0.077222738	PLIN5/DGAT2	2
BP	GO:0110149	regulation of biomineralization	3/96	99/18723	0.01437157	0.097591779	0.079735862	ALOX5/CEBPB/S1PR1	3
BP	GO:0042092	type 2 immune response	2/96	36/18723	0.014635615	0.09831997	0.080330819	ARG1/TBX21	2
BP	GO:0042119	neutrophil activation	2/96	36/18723	0.014635615	0.09831997	0.080330819	IL18RAP/CD177	2
BP	GO:0045652	regulation of megakaryocyte differentiation	2/96	36/18723	0.014635615	0.09831997	0.080330819	HMGB2/GATA2	2
BP	GO:0006641	triglyceride metabolic process	3/96	100/18723	0.014762008	0.098816144	0.08073621	PLIN5/DGAT2/ACSL1	3
BP	GO:0050830	defense response to Gram-positive bacterium	3/96	101/18723	0.015158424	0.100785605	0.082345328	PGLYRP1/HMGB2/GBP4	3
BP	GO:0006631	fatty acid metabolic process	6/96	390/18723	0.015163384	0.100785605	0.082345328	PLIN5/ALOX5AP/ALOX5/DGAT2/ACSL1/ALOX15	6
BP	GO:0045730	respiratory burst	2/96	37/18723	0.015420824	0.102135811	0.083448493	NCF4/SLC11A1	2
BP	GO:0019395	fatty acid oxidation	3/96	103/18723	0.015969232	0.105028413	0.085811848	PLIN5/DGAT2/ALOX15	3
BP	GO:0030593	neutrophil chemotaxis	3/96	103/18723	0.015969232	0.105028413	0.085811848	S100A9/S100A8/S100A12	3
BP	GO:0006882	cellular zinc ion homeostasis	2/96	38/18723	0.016223785	0.105539834	0.086229697	S100A9/S100A8	2
BP	GO:0035337	fatty-acyl-CoA metabolic process	2/96	38/18723	0.016223785	0.105539834	0.086229697	DGAT2/ACSL1	2
BP	GO:0034599	cellular response to oxidative stress	5/96	288/18723	0.016338972	0.105539834	0.086229697	ALOX5/VNN1/MMP9/IL18RAP/ARG1	5
BP	GO:0097193	intrinsic apoptotic signaling pathway	5/96	288/18723	0.016338972	0.105539834	0.086229697	VNN1/MMP9/CEBPB/S100A9/S100A8	5
BP	GO:0046634	regulation of alpha-beta T cell activation	3/96	104/18723	0.016383642	0.105539834	0.086229697	TBX21/RUNX3/CD3E	3
BP	GO:0048661	positive regulation of smooth muscle cell proliferation	3/96	104/18723	0.016383642	0.105539834	0.086229697	NQO2/MMP9/S1PR1	3
BP	GO:0044403	biological process involved in symbiotic interaction	5/96	290/18723	0.016784949	0.107755934	0.088040328	IFITM2/PGLYRP1/ARG1/IFIT1/CX3CR1	5
BP	GO:0090207	regulation of triglyceride metabolic process	2/96	39/18723	0.017044299	0.109048729	0.089096586	PLIN5/DGAT2	2
BP	GO:0002793	positive regulation of peptide secretion	3/96	106/18723	0.017230516	0.109126601	0.08916021	PFKFB2/GPR27/S100A8	3
BP	GO:0006766	vitamin metabolic process	3/96	106/18723	0.017230516	0.109126601	0.08916021	VNN1/VNN2/SLC2A3	3
BP	GO:0007218	neuropeptide signaling pathway	3/96	106/18723	0.017230516	0.109126601	0.08916021	LTB4R/PROK2/PTGDR2	3
BP	GO:0002824	positive regulation of adaptive immune response based on somatic recombination of immune receptors built from immunoglobulin superfamily domains	3/96	107/18723	0.017662994	0.111490238	0.091091384	IL18R1/SLC11A1/TBX21	3
BP	GO:0044275	cellular carbohydrate catabolic process	2/96	40/18723	0.017882169	0.112121197	0.091606899	MGAM/PYGL	2
BP	GO:0055069	zinc ion homeostasis	2/96	40/18723	0.017882169	0.112121197	0.091606899	S100A9/S100A8	2
BP	GO:0002832	negative regulation of response to biotic stimulus	3/96	108/18723	0.018101507	0.113119387	0.092422454	ARG1/IRAK3/OAS3	3
BP	GO:0042063	gliogenesis	5/96	301/18723	0.019383127	0.120328915	0.098312888	DAAM2/CDK5R1/S100A9/S100A8/CX3CR1	5
BP	GO:0042886	amide transport	5/96	301/18723	0.019383127	0.120328915	0.098312888	PFKFB2/ALOX5/FOLR3/GPR27/S100A8	5
BP	GO:0002791	regulation of peptide secretion	4/96	200/18723	0.019588988	0.121206866	0.099030205	PFKFB2/ALOX5/GPR27/S100A8	4
BP	GO:0007162	negative regulation of cell adhesion	5/96	303/18723	0.01988238	0.122026214	0.099699641	CEBPB/ARG1/TBX21/SPN/RUNX3	5
BP	GO:0001676	long-chain fatty acid metabolic process	3/96	112/18723	0.019916028	0.122026214	0.099699641	ALOX5/ACSL1/ALOX15	3
BP	GO:0002821	positive regulation of adaptive immune response	3/96	112/18723	0.019916028	0.122026214	0.099699641	IL18R1/SLC11A1/TBX21	3
BP	GO:0090087	regulation of peptide transport	4/96	202/18723	0.020234799	0.123184248	0.100645794	PFKFB2/ALOX5/GPR27/S100A8	4
BP	GO:0002708	positive regulation of lymphocyte mediated immunity	3/96	113/18723	0.020384795	0.123184248	0.100645794	IL18R1/IL18RAP/TBX21	3
BP	GO:0071347	cellular response to interleukin-1	3/96	113/18723	0.020384795	0.123184248	0.100645794	IL1R2/CEBPB/IRAK3	3
BP	GO:0014002	astrocyte development	2/96	43/18723	0.020497964	0.123184248	0.100645794	S100A9/S100A8	2
BP	GO:0036230	granulocyte activation	2/96	43/18723	0.020497964	0.123184248	0.100645794	IL18RAP/CD177	2
BP	GO:0046636	negative regulation of alpha-beta T cell activation	2/96	43/18723	0.020497964	0.123184248	0.100645794	TBX21/RUNX3	2
BP	GO:0002440	production of molecular mediator of immune response	5/96	308/18723	0.021167347	0.126801849	0.103601499	IL18R1/ARG1/IRAK3/CLC/TBX21	5
BP	GO:0046916	cellular transition metal ion homeostasis	3/96	115/18723	0.021340517	0.127002006	0.103765035	SLC11A1/S100A9/S100A8	3
BP	GO:0042554	superoxide anion generation	2/96	44/18723	0.021403315	0.127002006	0.103765035	NCF4/CD177	2
BP	GO:0046006	regulation of activated T cell proliferation	2/96	44/18723	0.021403315	0.127002006	0.103765035	ARG1/CLC	2
BP	GO:0051047	positive regulation of secretion	5/96	310/18723	0.021696197	0.128335052	0.104854179	PFKFB2/CD177/GPR27/S100A8/GATA2	5
BP	GO:0050679	positive regulation of epithelial cell proliferation	4/96	207/18723	0.021906443	0.129172474	0.105538382	LRG1/HMGB2/ARG1/GATA2	4
BP	GO:0072676	lymphocyte migration	3/96	117/18723	0.0223205	0.1304144	0.106553079	S1PR1/TBX21/SPN	3
BP	GO:0035987	endodermal cell differentiation	2/96	45/18723	0.022325059	0.1304144	0.106553079	MMP9/EOMES	2
BP	GO:0051180	vitamin transport	2/96	45/18723	0.022325059	0.1304144	0.106553079	FOLR3/SLC2A3	2
BP	GO:0071222	cellular response to lipopolysaccharide	4/96	209/18723	0.022598109	0.131600751	0.107522369	HMGB2/CEBPB/ARG1/CX3CR1	4
BP	GO:0007613	memory	3/96	118/18723	0.022819592	0.131667645	0.107577023	NQO2/CEBPB/CX3CR1	3
BP	GO:0051101	regulation of DNA binding	3/96	118/18723	0.022819592	0.131667645	0.107577023	MMP9/HMGB2/GZMA	3
BP	GO:0071346	cellular response to interferon-gamma	3/96	118/18723	0.022819592	0.131667645	0.107577023	ARG1/GBP5/GBP4	3
BP	GO:0043300	regulation of leukocyte degranulation	2/96	46/18723	0.023263008	0.133761758	0.109287986	CD177/GATA2	2
BP	GO:0030282	bone mineralization	3/96	119/18723	0.023324751	0.133761758	0.109287986	ALOX5/ALOX15/S1PR1	3
BP	GO:0019058	viral life cycle	5/96	317/18723	0.023614922	0.134457054	0.109856067	IFITM2/SLPI/IFIT1/OAS3/OAS2	5
BP	GO:0030258	lipid modification	4/96	212/18723	0.023660439	0.134457054	0.109856067	PLIN5/ALOX5/DGAT2/ALOX15	4
BP	GO:0043523	regulation of neuron apoptotic process	4/96	212/18723	0.023660439	0.134457054	0.109856067	CDK5R1/NQO2/CEBPB/CX3CR1	4
BP	GO:0007204	positive regulation of cytosolic calcium ion concentration	5/96	319/18723	0.024182674	0.135571813	0.110766864	FPR1/PROK2/GATA2/S1PR1/CX3CR1	5
BP	GO:0035850	epithelial cell differentiation involved in kidney development	2/96	47/18723	0.024216975	0.135571813	0.110766864	MMP9/BASP1	2
BP	GO:0045581	negative regulation of T cell differentiation	2/96	47/18723	0.024216975	0.135571813	0.110766864	TBX21/RUNX3	2
BP	GO:0045646	regulation of erythrocyte differentiation	2/96	47/18723	0.024216975	0.135571813	0.110766864	HMGB2/GATA2	2
BP	GO:0050798	activated T cell proliferation	2/96	47/18723	0.024216975	0.135571813	0.110766864	ARG1/CLC	2
BP	GO:0045785	positive regulation of cell adhesion	6/96	437/18723	0.024936161	0.139183736	0.11371793	FLOT2/ALOX5/VNN1/ALOX15/RUNX3/CD3E	6
BP	GO:0050870	positive regulation of T cell activation	4/96	216/18723	0.025123507	0.139814546	0.114233323	FLOT2/VNN1/RUNX3/CD3E	4
BP	GO:0006690	icosanoid metabolic process	3/96	123/18723	0.025406043	0.140969814	0.115177217	ALOX5AP/ALOX5/ALOX15	3
BP	GO:0018198	peptidyl-cysteine modification	2/96	49/18723	0.026172221	0.142695502	0.116587164	S100A9/S100A8	2
BP	GO:0030225	macrophage differentiation	2/96	49/18723	0.026172221	0.142695502	0.116587164	MMP9/GATA2	2
BP	GO:0032692	negative regulation of interleukin-1 production	2/96	49/18723	0.026172221	0.142695502	0.116587164	IL1R2/CX3CR1	2
BP	GO:0043330	response to exogenous dsRNA	2/96	49/18723	0.026172221	0.142695502	0.116587164	IRAK3/IFIT1	2
BP	GO:0070169	positive regulation of biomineral tissue development	2/96	49/18723	0.026172221	0.142695502	0.116587164	ALOX5/CEBPB	2
BP	GO:1904707	positive regulation of vascular associated smooth muscle cell proliferation	2/96	49/18723	0.026172221	0.142695502	0.116587164	NQO2/MMP9	2
BP	GO:0071621	granulocyte chemotaxis	3/96	125/18723	0.026483056	0.143972917	0.117630856	S100A9/S100A8/S100A12	3
BP	GO:0071219	cellular response to molecule of bacterial origin	4/96	221/18723	0.027027825	0.146454635	0.119658506	HMGB2/CEBPB/ARG1/CX3CR1	4
BP	GO:0050873	brown fat cell differentiation	2/96	50/18723	0.027173135	0.146454635	0.119658506	LRG1/CEBPB	2
BP	GO:0110151	positive regulation of biomineralization	2/96	50/18723	0.027173135	0.146454635	0.119658506	ALOX5/CEBPB	2
BP	GO:0006639	acylglycerol metabolic process	3/96	128/18723	0.028143966	0.149900477	0.122473878	PLIN5/DGAT2/ACSL1	3
BP	GO:0043370	regulation of CD4-positive, alpha-beta T cell differentiation	2/96	51/18723	0.028189335	0.149900477	0.122473878	TBX21/RUNX3	2
BP	GO:0048013	ephrin receptor signaling pathway	2/96	51/18723	0.028189335	0.149900477	0.122473878	CDK5R1/MMP9	2
BP	GO:1903307	positive regulation of regulated secretory pathway	2/96	51/18723	0.028189335	0.149900477	0.122473878	CD177/GATA2	2
BP	GO:2001234	negative regulation of apoptotic signaling pathway	4/96	224/18723	0.028210935	0.149900477	0.122473878	VNN1/MMP9/HMGB2/CX3CR1	4
BP	GO:0006638	neutral lipid metabolic process	3/96	129/18723	0.02870969	0.152120921	0.124288057	PLIN5/DGAT2/ACSL1	3
BP	GO:0062197	cellular response to chemical stress	5/96	337/18723	0.029691786	0.156882724	0.128178616	ALOX5/VNN1/MMP9/IL18RAP/ARG1	5
BP	GO:0045089	positive regulation of innate immune response	3/96	131/18723	0.029859236	0.157325555	0.128540424	IL18RAP/HMGB2/GBP5	3
BP	GO:0045444	fat cell differentiation	4/96	229/18723	0.030250729	0.157706339	0.128851537	ALOX5/LRG1/CEBPB/GATA2	4
BP	GO:0071695	anatomical structure maturation	4/96	229/18723	0.030250729	0.157706339	0.128851537	CDK5R1/GATA2/S1PR1/CX3CR1	4
BP	GO:0002707	negative regulation of lymphocyte mediated immunity	2/96	53/18723	0.030266873	0.157706339	0.128851537	ARG1/TBX21	2
BP	GO:0008347	glial cell migration	2/96	53/18723	0.030266873	0.157706339	0.128851537	CDK5R1/CX3CR1	2
BP	GO:0001706	endoderm formation	2/96	54/18723	0.031327857	0.162335259	0.132633525	MMP9/EOMES	2
BP	GO:0002823	negative regulation of adaptive immune response based on somatic recombination of immune receptors built from immunoglobulin superfamily domains	2/96	54/18723	0.031327857	0.162335259	0.132633525	ARG1/TBX21	2
BP	GO:0043331	response to dsRNA	2/96	55/18723	0.032403417	0.167447326	0.136810261	IRAK3/IFIT1	2
BP	GO:0033674	positive regulation of kinase activity	6/96	467/18723	0.032995923	0.170042001	0.1389302	CDK5R1/PFKFB2/SLC11A1/ACSL1/LILRA5/S100A12	6
BP	GO:0030879	mammary gland development	3/96	137/18723	0.033452196	0.170734536	0.139496025	CEBPB/ARG1/OAS2	3
BP	GO:0001912	positive regulation of leukocyte mediated cytotoxicity	2/96	56/18723	0.033493378	0.170734536	0.139496025	IL18RAP/ARG1	2
BP	GO:0043388	positive regulation of DNA binding	2/96	56/18723	0.033493378	0.170734536	0.139496025	MMP9/HMGB2	2
BP	GO:0046456	icosanoid biosynthetic process	2/96	56/18723	0.033493378	0.170734536	0.139496025	ALOX5AP/ALOX5	2
BP	GO:0055076	transition metal ion homeostasis	3/96	138/18723	0.03407199	0.17321463	0.141522348	SLC11A1/S100A9/S100A8	3

BP	GO:0030219	megakaryocyte differentiation	2/96	57/18723	0.034597568	0.174940929	0.142932794	HMGB2/GATA2	2
BP	GO:0061005	cell differentiation involved in kidney development	2/96	57/18723	0.034597568	0.174940929	0.142932794	MMP9/BASP1	2
BP	GO:0045598	regulation of fat cell differentiation	3/96	139/18723	0.034697751	0.174977132	0.142962373	ALOX5/CEBPB/GATA2	3
BP	GO:0051480	regulation of cytosolic calcium ion concentration	5/96	353/18723	0.035207007	0.177070537	0.144672758	FPR1/PROK2/GATA2/S1PR1/CX3CR1	5
BP	GO:0015711	organic anion transport	5/96	354/18723	0.035571418	0.177728705	0.145210503	FOLR3/SLC11A1/CA4/SLC2A3/ARG1	5
BP	GO:0010043	response to zinc ion	2/96	58/18723	0.035715816	0.177728705	0.145210503	ARG1/S100A8	2
BP	GO:0043525	positive regulation of neuron apoptotic process	2/96	58/18723	0.035715816	0.177728705	0.145210503	CDK5R1/NQO2	2
BP	GO:2001244	positive regulation of intrinsic apoptotic signaling pathway	2/96	58/18723	0.035715816	0.177728705	0.145210503	S100A9/S100A8	2
BP	GO:0002790	peptide secretion	4/96	242/18723	0.035954741	0.178445563	0.145796201	PFKFB2/ALOX5/GPR27/S100A8	4
BP	GO:0019369	arachidonic acid metabolic process	2/96	59/18723	0.036847952	0.182397363	0.149024959	ALOX5/ALOX15	2
BP	GO:0070555	response to interleukin-1	3/96	143/18723	0.037260275	0.183954271	0.150297006	IL1R2/CEBPB/IRAK3	3
BP	GO:0051402	neuron apoptotic process	4/96	246/18723	0.037826918	0.184765479	0.150959791	CDK5R1/NQO2/CEBPB/CX3CR1	4
BP	GO:0071216	cellular response to biotic stimulus	4/96	246/18723	0.037826918	0.184765479	0.150959791	HMGB2/CEBPB/ARG1/CX3CR1	4
BP	GO:0001890	placenta development	3/96	144/18723	0.037915723	0.184765479	0.150959791	CEBPB/GATA2/EOMES	3
BP	GO:0106106	cold-induced thermogenesis	3/96	144/18723	0.037915723	0.184765479	0.150959791	IL18R1/ACSL1/CEBPB	3
BP	GO:0120161	regulation of cold-induced thermogenesis	3/96	144/18723	0.037915723	0.184765479	0.150959791	IL18R1/ACSL1/CEBPB	3
BP	GO:0051251	positive regulation of lymphocyte activation	5/96	362/18723	0.038571144	0.187473701	0.153172503	FLOT2/VNN1/TBX21/RUNX3/CD3E	5
BP	GO:0051098	regulation of binding	5/96	363/18723	0.038956707	0.188859707	0.154304918	MMP9/HMGB2/SLPI/IFIT1/GZMA	5
BP	GO:0007189	adenylate cyclase-activating G protein-coupled receptor signaling pathway	3/96	146/18723	0.039244321	0.189278377	0.154646986	ADGRG3/S1PR5/S1PR1	3
BP	GO:0045580	regulation of T cell differentiation	3/96	146/18723	0.039244321	0.189278377	0.154646986	VNN1/TBX21/RUNX3	3
BP	GO:0071560	cellular response to transforming growth factor beta stimulus	4/96	250/18723	0.039754402	0.191248161	0.156256367	LRG1/ARG1/BCL9L/CX3CR1	4
BP	GO:0032615	interleukin-12 production	2/96	62/18723	0.040326009	0.191548541	0.156501788	LILRA5/IRAK3	2
BP	GO:0032623	interleukin-2 production	2/96	62/18723	0.040326009	0.191548541	0.156501788	TBX21/CD3E	2
BP	GO:0032655	regulation of interleukin-12 production	2/96	62/18723	0.040326009	0.191548541	0.156501788	LILRA5/IRAK3	2
BP	GO:0032663	regulation of interleukin-2 production	2/96	62/18723	0.040326009	0.191548541	0.156501788	TBX21/CD3E	2
BP	GO:0045576	mast cell activation	2/96	62/18723	0.040326009	0.191548541	0.156501788	S100A12/GATA2	2
BP	GO:0045017	glycerolipid biosynthetic process	4/96	252/18723	0.040738905	0.193022368	0.157705956	PLIN5/DGAT2/ACSL1/ALOX15	4
BP	GO:0030902	hindbrain development	3/96	151/18723	0.0426685	0.200848937	0.164100533	CDK5R1/GATA2/CD3E	3
BP	GO:0048857	neural nucleus development	2/96	64/18723	0.042711098	0.200848937	0.164100533	CDK5R1/BASP1	2
BP	GO:0070542	response to fatty acid	2/96	64/18723	0.042711098	0.200848937	0.164100533	DGAT2/ACSL1	2
BP	GO:0010038	response to metal ion	5/96	373/18723	0.042942719	0.20143455	0.164578999	ALOX5AP/MMP9/ARG1/S100A8/ALOX15	5
BP	GO:0045165	cell fate commitment	4/96	258/18723	0.043775492	0.204830102	0.167353283	GATA2/EOMES/TBX21/SPN	4
BP	GO:0071677	positive regulation of mononuclear cell migration	2/96	65/18723	0.043923068	0.205010646	0.167500794	CX3CR1/SPN	2
BP	GO:0120254	olefinic compound metabolic process	3/96	153/18723	0.044078968	0.205229056	0.167679242	ALOX5/DGAT2/ALOX15	3
BP	GO:0016052	carbohydrate catabolic process	3/96	154/18723	0.044792888	0.207129175	0.169231705	PFKFB2/MGAM/PYGL	3
BP	GO:0002562	somatic diversification of immune receptors via germline recombination within a single locus	2/96	66/18723	0.045147773	0.207129175	0.169231705	HMGB2/TBX21	2
BP	GO:0016444	somatic cell DNA recombination	2/96	66/18723	0.045147773	0.207129175	0.169231705	HMGB2/TBX21	2
BP	GO:0032715	negative regulation of interleukin-6 production	2/96	66/18723	0.045147773	0.207129175	0.169231705	ORM1/IRAK3	2
BP	GO:0050918	positive chemotaxis	2/96	66/18723	0.045147773	0.207129175	0.169231705	HMGB2/S1PR1	2
BP	GO:0072678	T cell migration	2/96	66/18723	0.045147773	0.207129175	0.169231705	S1PR1/SPN	2
BP	GO:0034614	cellular response to reactive oxygen species	3/96	155/18723	0.04551258	0.208294799	0.17018406	MMP9/IL18RAP/ARG1	3
BP	GO:0032890	regulation of organic acid transport	2/96	67/18723	0.046385055	0.21074949	0.172189628	ACSL1/ARG1	2
BP	GO:0046635	positive regulation of alpha-beta T cell activation	2/96	67/18723	0.046385055	0.21074949	0.172189628	RUNX3/CD3E	2
BP	GO:2000514	regulation of CD4-positive, alpha-beta T cell activation	2/96	67/18723	0.046385055	0.21074949	0.172189628	TBX21/RUNX3	2
BP	GO:0015833	peptide transport	4/96	264/18723	0.046936662	0.212377628	0.173519873	PFKFB2/ALOX5/GPR27/S100A8	4
BP	GO:1990845	adaptive thermogenesis	3/96	157/18723	0.046969215	0.212377628	0.173519873	IL18R1/ACSL1/CEBPB	3
BP	GO:0009988	cell-cell recognition	2/96	68/18723	0.047634755	0.213844808	0.17471861	FOLR3/PRF1	2
BP	GO:0046637	regulation of alpha-beta T cell differentiation	2/96	68/18723	0.047634755	0.213844808	0.17471861	TBX21/RUNX3	2
BP	GO:0046686	response to cadmium ion	2/96	68/18723	0.047634755	0.213844808	0.17471861	MMP9/ARG1	2
BP	GO:0045860	positive regulation of protein kinase activity	5/96	386/18723	0.048481265	0.217126809	0.177400118	CDK5R1/SLC11A1/ACSL1/LILRA5/S100A12	5
BP	GO:0015807	L-amino acid transport	2/96	69/18723	0.048896716	0.217445261	0.177660304	SLC11A1/ARG1	2
CC	GO:0070820	tertiary granule	12/101	164/19550	5.06E-11	5.53E-09	4.53E-09	DOK3/ORM1/PGLYRP1/MMP9/FOLR3/MGAM/CD177/SLC11A1/FPR1/LRG1/SLC2A3/MCEMP1	12
CC	GO:0034774	secretory granule lumen	15/101	322/19550	1.07E-10	5.53E-09	4.53E-09	S100A11/S100P/ORM1/PGLYRP1/ALOX5/FOLR3/LRG1/GCA/PYGL/S100A9/SLPI/ARG1/S100A8/S100A12/CTSW	15
CC	GO:0060205	cytoplasmic vesicle lumen	15/101	325/19550	1.22E-10	5.53E-09	4.53E-09	S100A11/S100P/ORM1/PGLYRP1/ALOX5/FOLR3/LRG1/GCA/PYGL/S100A9/SLPI/ARG1/S100A8/S100A12/CTSW	15
CC	GO:0031983	vesicle lumen	15/101	327/19550	1.32E-10	5.53E-09	4.53E-09	S100A11/S100P/ORM1/PGLYRP1/ALOX5/FOLR3/LRG1/GCA/PYGL/S100A9/SLPI/ARG1/S100A8/S100A12/CTSW	15
CC	GO:0042581	specific granule	10/101	160/19550	1.03E-08	3.44E-07	2.82E-07	ADGRG3/ORM1/PGLYRP1/FOLR3/CD177/LRG1/SLC2A3/SLPI/MCEMP1/ARG1	10
CC	GO:0101002	ficolin-1-rich granule	9/101	185/19550	4.95E-07	1.38E-05	1.13E-05	DOK3/ALOX5/MMP9/MGAM/SLC11A1/FPR1/LRG1/PYGL/SLC2A3	9
CC	GO:0030667	secretory granule membrane	11/101	311/19550	6.17E-07	1.47E-05	1.21E-05	FLOT2/DOK3/ADGRG3/VNN1/MGAM/CD177/SLC11A1/FPR1/CA4/SLC2A3/MCEMP1	11
CC	GO:0035580	specific granule lumen	6/101	62/19550	7.96E-07	1.66E-05	1.36E-05	ORM1/PGLYRP1/FOLR3/LRG1/SLPI/ARG1	6
CC	GO:0009897	external side of plasma membrane	12/101	421/19550	1.80E-06	3.34E-05	2.74E-05	CD163/FOLR3/CA4/CD3D/CD8A/FCRL6/SLAMF7/S1PR1/CX3CR1/SPN/CD3E/IL2RB	12
CC	GO:1904724	tertiary granule lumen	5/101	55/19550	9.44E-06	0.000157647	0.000129178	ORM1/PGLYRP1/MMP9/FOLR3/LRG1	5
CC	GO:0101003	ficolin-1-rich granule membrane	5/101	61/19550	1.58E-05	0.000239155	0.000195967	DOK3/MGAM/SLC11A1/FPR1/SLC2A3	5
CC	GO:0031225	anchored component of membrane	7/101	170/19550	2.88E-05	0.000400255	0.000327974	FLOT2/VNN1/FOLR3/CD177/VNN2/ALPL/CA4	7
CC	GO:0070821	tertiary granule membrane	5/101	73/19550	3.79E-05	0.000486496	0.000398642	MGAM/CD177/SLC11A1/SLC2A3/MCEMP1	5
CC	GO:0030139	endocytic vesicle	9/101	336/19550	6.13E-05	0.00073115	0.000599115	FLOT2/CD163/CLEC4E/PGLYRP1/NCF4/SLC11A1/PLD4/GNLY/CD3D	9
CC	GO:0045335	phagocytic vesicle	6/101	137/19550	7.79E-05	0.000867511	0.000710851	CLEC4E/PGLYRP1/NCF4/SLC11A1/PLD4/GNLY	6
CC	GO:0046658	anchored component of plasma membrane	4/101	63/19550	0.000316356	0.003301967	0.002705677	FLOT2/FOLR3/CD177/CA4	4
CC	GO:0035579	specific granule membrane	4/101	91/19550	0.001271166	0.012487337	0.010232297	ADGRG3/CD177/SLC2A3/MCEMP1	4
CC	GO:0001772	immunological synapse	3/101	44/19550	0.001519882	0.014101128	0.011554659	GZMA/GZMB/CD3E	3
CC	GO:0001931	uropod	2/101	13/19550	0.001986183	0.016584626	0.013589672	FLOT2/SPN	2
CC	GO:0031254	cell trailing edge	2/101	13/19550	0.001986183	0.016584626	0.013589672	FLOT2/SPN	2
CC	GO:1904813	ficolin-1-rich granule lumen	4/101	124/19550	0.003920004	0.031173365	0.025543886	ALOX5/MMP9/LRG1/PYGL	4
CC	GO:0031362	anchored component of external side of plasma membrane	2/101	20/19550	0.004725552	0.035871236	0.029393386	FOLR3/CA4	2
CC	GO:0098802	plasma membrane signaling receptor complex	6/101	306/19550	0.00512245	0.037193445	0.030476822	IL18R1/IL18RAP/CD3D/CD8A/CD3E/IL2RB	6
CC	GO:0071682	endocytic vesicle lumen	2/101	23/19550	0.006229392	0.043346187	0.035518464	PGLYRP1/GNLY	2
CC	GO:0031233	intrinsic component of external side of plasma membrane	2/101	24/19550	0.006772944	0.045243263	0.037072954	FOLR3/CA4	2
CC	GO:0005766	primary lysosome	4/101	155/19550	0.008563036	0.052963961	0.0433994	VNN1/FPR1/GCA/ARG1	4
CC	GO:0042582	azurophil granule	4/101	155/19550	0.008563036	0.052963961	0.0433994	VNN1/FPR1/GCA/ARG1	4
CC	GO:0005811	lipid droplet	3/101	96/19550	0.013518562	0.080628566	0.06606816	PLIN5/DGAT2/ALOX15	3
CC	GO:0030666	endocytic vesicle membrane	4/101	193/19550	0.01789715	0.103062899	0.084451162	CD163/CLEC4E/SLC11A1/CD3D	4
CC	GO:0016363	nuclear matrix	3/101	111/19550	0.019859052	0.110548725	0.090585151	ALOX5/BASP1/CEBPB	3
CC	GO:0044853	plasma membrane raft	3/101	116/19550	0.02227919	0.11965241	0.098044836	FLOT2/CD177/CD8A	3
CC	GO:0062023	collagen-containing extracellular matrix	6/101	425/19550	0.022927408	0.11965241	0.098044836	ORM1/MMP9/S100A9/SLPI/S100A8/CLC	6
CC	GO:0034399	nuclear periphery	3/101	130/19550	0.02987424	0.142819946	0.117028635	ALOX5/BASP1/CEBPB	3
CC	GO:0045121	membrane raft	5/101	335/19550	0.029932324	0.142819946	0.117028635	FLOT2/CD177/LTB4R/CD8A/S1PR1	5
CC	GO:0098857	membrane microdomain	5/101	335/19550	0.029932324	0.142819946	0.117028635	FLOT2/CD177/LTB4R/CD8A/S1PR1	5
CC	GO:0035577	azurophil granule membrane	2/101	58/19550	0.036220781	0.16802418	0.137681333	VNN1/FPR1	2
CC	GO:0042101	T cell receptor complex	3/101	148/19550	0.041395111	0.186837391	0.153097137	CD3D/CD8A/CD3E	3
MF	GO:0050786	RAGE receptor binding	5/98	10/18368	9.62E-10	2.54E-07	2.27E-07	FPR1/HMGB2/S100A9/S100A8/S100A12	5
MF	GO:0048306	calcium-dependent protein binding	6/98	88/18368	7.53E-06	0.000993377	0.000887227	S100A11/S100P/CD177/S100A9/S100A8/S100A12	6
MF	GO:0140375	immune receptor activity	7/98	144/18368	1.20E-05	0.001055772	0.000942954	IL18R1/IL1R2/IL18RAP/FPR1/LILRA5/CX3CR1/IL2RB	7
MF	GO:0036041	long-chain fatty acid binding	3/98	14/18368	5.14E-05	0.003390391	0.0030281	ALOX5AP/S100A9/S100A8	3
MF	GO:0004896	cytokine receptor activity	5/98	97/18368	0.000170554	0.009005265	0.008042979	IL18R1/IL1R2/IL18RAP/CX3CR1/IL2RB	5
MF	GO:0016702	oxidoreductase activity, acting on single donors with incorporation of molecular oxygen, incorporation of two atoms of oxygen	3/98	23/18368	0.000241368	0.010363253	0.009255856	ALOX5AP/ALOX5/ALOX15	3
MF	GO:0016701	oxidoreductase activity, acting on single donors with incorporation of molecular oxygen	3/98	24/18368	0.000274783	0.010363253	0.009255856	ALOX5AP/ALOX5/ALOX15	3
MF	GO:0031406	carboxylic acid binding	6/98	176/18368	0.000360953	0.011911457	0.010638622	ALOX5AP/PADI4/FOLR3/PYGL/S100A9/S100A8	6
MF	GO:0016810	hydrolase activity, acting on carbon-nitrogen (but not peptide) bonds	5/98	125/18368	0.000551924	0.016189781	0.014459773	PGLYRP1/PADI4/VNN1/VNN2/ARG1	5
MF	GO:0033293	monocarboxylic acid binding	4/98	73/18368	0.000625561	0.016514822	0.01475008	ALOX5AP/PYGL/S100A9/S100A8	4
MF	GO:0043177	organic acid binding	5/98	135/18368	0.000782446	0.018778709	0.016772053	ALOX5AP/PADI4/FOLR3/S100A9/S100A8	5
MF	GO:0005504	fatty acid binding	3/98	39/18368	0.001170878	0.025759324	0.023006733	ALOX5AP/S100A9/S100A8	3
MF	GO:0005536	glucose binding	2/98	11/18368	0.001501889	0.028321337	0.025294974	PYGL/SLC2A3	2
MF	GO:0016813	hydrolase activity, acting on carbon-nitrogen (but not peptide) bonds, in linear amidines	2/98	11/18368	0.001501889	0.028321337	0.025294974	PADI4/ARG1	2
MF	GO:0035325	Toll-like receptor binding	2/98	12/18368	0.001796009	0.031609759	0.028232002	S100A9/S100A8	2
MF	GO:0004953	icosanoid receptor activity	2/98	15/18368	0.002827651	0.043911751	0.039219427	LTB4R/PTGDR2	2
MF	GO:0045125	bioactive lipid receptor activity	2/98	15/18368	0.002827651	0.043911751	0.039219427	S1PR5/S1PR1	2
MF	GO:0050135	NAD(P)+ nucleosidase activity	2/98	16/18368	0.003220398	0.044746582	0.039965049	IL18R1/IL18RAP	2
MF	GO:0061809	NAD+ nucleotidase, cyclic ADP-ribose generating	2/98	16/18368	0.003220398	0.044746582	0.039965049	IL18R1/IL18RAP	2
MF	GO:0016811	hydrolase activity, acting on carbon-nitrogen (but not peptide) bonds, in linear amides	3/98	71/18368	0.00647542	0.082376981	0.073574337	PGLYRP1/VNN1/VNN2	3
MF	GO:0019955	cytokine binding	4/98	139/18368	0.006552714	0.082376981	0.073574337	IL18R1/IL1R2/CX3CR1/IL2RB	4
MF	GO:0038187	pattern recognition receptor activity	2/98	26/18368	0.008424879	0.101098552	0.090295358	CLEC4E/PGLYRP1	2
MF	GO:0035255	ionotropic glutamate receptor binding	2/98	27/18368	0.009067468	0.104078761	0.092957107	FLOT2/CDK5R1	2
MF	GO:0003953	NAD+ nucleosidase activity	2/98	28/18368	0.009731278	0.107044063	0.095605543	IL18R1/IL18RAP	2
MF	GO:0070566	adenylyltransferase activity	2/98	30/18368	0.011121608	0.117444183	0.104894327	OAS3/OAS2	2
MF	GO:0051213	dioxygenase activity	3/98	92/18368	0.013131397	0.133334189	0.119086357	ALOX5AP/ALOX5/ALOX15	3
MF	GO:0004252	serine-type endopeptidase activity	4/98	174/18368	0.014104307	0.137908776	0.123172112	MMP9/GZMH/GZMA/GZMB	4
MF	GO:0030246	carbohydrate binding	5/98	271/18368	0.015052795	0.141926357	0.126760382	CLEC4E/MGAM/PYGL/SLC2A3/CLC	5
MF	GO:0008236	serine-type peptidase activity	4/98	191/18368	0.01919187	0.164079191	0.146546008	MMP9/GZMH/GZMA/GZMB	4
MF	GO:0042287	MHC protein binding	2/98	40/18368	0.019266875	0.164079191	0.146546008	CD8A/FCRL6	2
MF	GO:0043014	alpha-tubulin binding	2/98	40/18368	0.019266875	0.164079191	0.146546008	CDK5R1/ARL4C	2
MF	GO:0017171	serine hydrolase activity	4/98	195/18368	0.020533905	0.169404716	0.151302457	MMP9/GZMH/GZMA/GZMB	4
MF	GO:0035254	glutamate receptor binding	2/98	47/18368	0.026067912	0.208543296	0.186258765	FLOT2/CDK5R1	2
MF	GO:0004175	endopeptidase activity	6/98	430/18368	0.027593239	0.214253382	0.191358682	MMP9/CTSW/GZMH/CLC/GZMA/GZMB	6
MF	GO:0016799	hydrolase activity, hydrolyzing N-glycosyl compounds	2/98	50/18368	0.029238387	0.220540979	0.196974399	IL18R1/IL18RAP	2
MF	GO:0002020	protease binding	3/98	135/18368	0.035627891	0.2612712	0.233352268	FLOT2/CDK5R1/CD177	3
MF	GO:0019838	growth factor binding	3/98	141/18368	0.03972403	0.271728031	0.242691702	IL1R2/FGFBP2/IL2RB	3

MF	GO:0016798	hydrolase activity, acting on glycosyl bonds	3/98	144/18368	0.041858124	0.271728031	0.242691702	IL18R1/MGAM/IL18RAP	3
MF	GO:0008528	G protein-coupled peptide receptor activity	3/98	147/18368	0.044049046	0.271728031	0.242691702	FPR1/LTB4R/CX3CR1	3
MF	GO:0001653	peptide receptor activity	3/98	153/18368	0.048599618	0.271728031	0.242691702	FPR1/LTB4R/CX3CR1	3
