# Supplementary material for: Integrated bioinformatics analysis of the effects of chronic pain on patients with spinal cord injury
Source: Front Cell Neurosci. 2025 Feb 5;19:1457740. doi: 10.3389/fncel.2025.1457740 (PMC11835904; doi:10.3389/fncel.2025.1457740)
Supplement: Supplementary Table S2-2 — Candidate items of GO analysis in GSE177034. [file Data_Sheet_4.pdf]

ONTOLOGY	ID	Description	GeneRatio	BgRatio	pvalue	p.adjust	qvalue	geneID	Count
BP	GO:0019835	cytolysis	2023/4/14	32/18723	6.95E-09	4.28E-06	2.33E-06	GZMH/GZMB/PRF1/GZMA	4
BP	GO:0050832	defense response to fungus	2023/4/14	49/18723	4.06E-08	1.25E-05	6.82E-06	GNLY/S100A12/CX3CR1/S100A8	4
BP	GO:0009620	response to fungus	2023/4/14	60/18723	9.31E-08	1.91E-05	1.04E-05	GNLY/S100A12/CX3CR1/S100A8	4
BP	GO:0001906	cell killing	2023/5/14	188/18723	1.80E-07	2.77E-05	1.51E-05	GZMB/PRF1/GNLY/S100A12/CX3CR1	5
BP	GO:0050900	leukocyte migration	2023/5/14	369/18723	5.00E-06	0.000616586	0.000336109	TBX21/MMP9/S100A12/CX3CR1/S100A8	5
BP	GO:0006968	cellular defense response	2023/3/14	54/18723	8.07E-06	0.000828668	0.000451717	PRF1/GNLY/CX3CR1	3
BP	GO:0002443	leukocyte mediated immunity	2023/5/14	440/18723	1.18E-05	0.001036057	0.000564768	TBX21/GZMB/PRF1/CX3CR1/CD8A	5
BP	GO:0031640	killing of cells of other organism	2023/3/14	68/18723	1.62E-05	0.001247999	0.0006803	PRF1/GNLY/S100A12	3
BP	GO:0030217	T cell differentiation	2023/4/14	257/18723	3.12E-05	0.002132216	0.001162298	TBX21/EOMES/CD8A/CD3E	4
BP	GO:0002456	T cell mediated immunity	2023/3/14	109/18723	6.67E-05	0.004107267	0.002238924	TBX21/PRF1/CD8A	3
BP	GO:0001909	leukocyte mediated cytotoxicity	2023/3/14	124/18723	9.78E-05	0.005124764	0.002793575	GZMB/PRF1/CX3CR1	3
BP	GO:0002449	lymphocyte mediated immunity	2023/4/14	350/18723	0.000103597	0.005124764	0.002793575	TBX21/GZMB/PRF1/CD8A	4
BP	GO:0070269	pyroptosis	2023/2/14	21/18723	0.000108152	0.005124764	0.002793575	GZMB/GZMA	2
BP	GO:0007159	leukocyte cell-cell adhesion	2023/4/14	371/18723	0.000129736	0.005495835	0.00299585	TBX21/CX3CR1/S100A8/CD3E	4
BP	GO:0030098	lymphocyte differentiation	2023/4/14	374/18723	0.000133827	0.005495835	0.00299585	TBX21/EOMES/CD8A/CD3E	4
BP	GO:0051092	positive regulation of NF-kappaB transcription factor activity	2023/3/14	152/18723	0.000178815	0.006884388	0.003752768	S100A12/CX3CR1/S100A8	3
BP	GO:0046631	alpha-beta T cell activation	2023/3/14	156/18723	0.000193065	0.006995751	0.003813474	TBX21/EOMES/CD3E	3
BP	GO:1903131	mononuclear cell differentiation	2023/4/14	426/18723	0.000220698	0.00755276	0.004117106	TBX21/EOMES/CD8A/CD3E	4
BP	GO:0051090	regulation of DNA-binding transcription factor activity	2023/4/14	440/18723	0.000249768	0.008097748	0.004414186	EOMES/S100A12/CX3CR1/S100A8	4
BP	GO:0043368	positive T cell selection	2023/2/14	37/18723	0.000340661	0.010492362	0.00571952	TBX21/CD3E	2
BP	GO:0042110	T cell activation	2023/4/14	487/18723	0.000367778	0.010575514	0.005764848	TBX21/EOMES/CD8A/CD3E	4
BP	GO:0071674	mononuclear cell migration	2023/3/14	196/18723	0.000377697	0.010575514	0.005764848	TBX21/S100A12/CX3CR1	3
BP	GO:0035987	endodermal cell differentiation	2023/2/14	45/18723	0.00050466	0.013516123	0.007367812	MMP9/EOMES	2
BP	GO:0097529	myeloid leukocyte migration	2023/3/14	220/18723	0.00052938	0.013587429	0.007406681	S100A12/CX3CR1/S100A8	3
BP	GO:0030595	leukocyte chemotaxis	2023/3/14	230/18723	0.000602591	0.014763182	0.008047599	S100A12/CX3CR1/S100A8	3
BP	GO:0045058	T cell selection	2023/2/14	50/18723	0.000623121	0.014763182	0.008047599	TBX21/CD3E	2
BP	GO:0001706	endoderm formation	2023/2/14	54/18723	0.000726665	0.016578724	0.009037274	MMP9/EOMES	2
BP	GO:0051091	positive regulation of DNA-binding transcription factor activity	2023/3/14	260/18723	0.000860314	0.018926901	0.010317296	S100A12/CX3CR1/S100A8	3
BP	GO:0032623	interleukin-2 production	2023/2/14	62/18723	0.000956978	0.01947868	0.010618077	TBX21/CD3E	2
BP	GO:0032663	regulation of interleukin-2 production	2023/2/14	62/18723	0.000956978	0.01947868	0.010618077	TBX21/CD3E	2
BP	GO:0002704	negative regulation of leukocyte mediated immunity	2023/2/14	63/18723	0.000987933	0.01947868	0.010618077	TBX21/CX3CR1	2
BP	GO:0002366	leukocyte activation involved in immune response	2023/3/14	275/18723	0.001011879	0.01947868	0.010618077	TBX21/EOMES/CX3CR1	3
BP	GO:0002263	cell activation involved in immune response	2023/3/14	279/18723	0.001054978	0.019692916	0.01073486	TBX21/EOMES/CX3CR1	3
BP	GO:0002287	alpha-beta T cell activation involved in immune response	2023/2/14	69/18723	0.001183697	0.02083306	0.011356367	TBX21/EOMES	2
BP	GO:0002293	alpha-beta T cell differentiation involved in immune response	2023/2/14	69/18723	0.001183697	0.02083306	0.011356367	TBX21/EOMES	2
BP	GO:0002548	monocyte chemotaxis	2023/2/14	70/18723	0.001217991	0.020841179	0.011360793	S100A12/CX3CR1	2
BP	GO:0010822	positive regulation of mitochondrion organization	2023/2/14	74/18723	0.001359909	0.022570712	0.012303584	MMP9/GZMB	2
BP	GO:0002292	T cell differentiation involved in immune response	2023/2/14	75/18723	0.00139657	0.022570712	0.012303584	TBX21/EOMES	2
BP	GO:0060326	cell chemotaxis	2023/3/14	310/18723	0.00142899	0.022570712	0.012303584	S100A12/CX3CR1/S100A8	3
BP	GO:0007492	endoderm development	2023/2/14	77/18723	0.001471306	0.02265812	0.012351231	MMP9/EOMES	2
BP	GO:0061844	antimicrobial humoral immune response mediated by antimicrobial peptide	2023/2/14	79/18723	0.001547924	0.023256615	0.012677478	GNLY/S100A12	2
BP	GO:0042742	defense response to bacterium	2023/3/14	350/18723	0.002022751	0.029064703	0.015843541	GNLY/S100A12/S100A8	3
BP	GO:0001708	cell fate specification	2023/2/14	92/18723	0.002091447	0.029064703	0.015843541	TBX21/EOMES	2
BP	GO:0002460	adaptive immune response based on somatic recombination of immune receptors built from immunoglobulin superfamily domains	2023/3/14	356/18723	0.002123233	0.029064703	0.015843541	TBX21/PRF1/CD8A	3
BP	GO:2001233	regulation of apoptotic signaling pathway	2023/3/14	356/18723	0.002123233	0.029064703	0.015843541	MMP9/CX3CR1/S100A8	3
BP	GO:0031341	regulation of cell killing	2023/2/14	99/18723	0.002416469	0.032359668	0.017639669	PRF1/CX3CR1	2
BP	GO:0030593	neutrophil chemotaxis	2023/2/14	103/18723	0.002612257	0.033597799	0.01831459	S100A12/S100A8	2
BP	GO:0046634	regulation of alpha-beta T cell activation	2023/2/14	104/18723	0.002662341	0.033597799	0.01831459	TBX21/CD3E	2
BP	GO:0050727	regulation of inflammatory response	2023/3/14	386/18723	0.002672552	0.033597799	0.01831459	MMP9/S100A12/S100A8	3
BP	GO:0035821	modulation of process of other organism	2023/2/14	106/18723	0.002763869	0.03400456	0.01853632	PRF1/CX3CR1	2
BP	GO:0008637	apoptotic mitochondrial changes	2023/2/14	107/18723	0.002815313	0.03400456	0.01853632	MMP9/GZMB	2
BP	GO:0002698	negative regulation of immune effector process	2023/2/14	110/18723	0.002972351	0.035210929	0.019193927	TBX21/CX3CR1	2
BP	GO:0046632	alpha-beta T cell differentiation	2023/2/14	112/18723	0.003079295	0.035789538	0.019509334	TBX21/EOMES	2
BP	GO:0002286	T cell activation involved in immune response	2023/2/14	114/18723	0.003188033	0.036367196	0.019824223	TBX21/EOMES	2
BP	GO:0051101	regulation of DNA binding	2023/2/14	118/18723	0.003410877	0.038012889	0.020721312	MMP9/GZMA	2
BP	GO:0032103	positive regulation of response to external stimulus	2023/3/14	427/18723	0.003555378	0.038012889	0.020721312	S100A12/CX3CR1/S100A8	3
BP	GO:0001704	formation of primary germ layer	2023/2/14	121/18723	0.003582687	0.038012889	0.020721312	MMP9/EOMES	2
BP	GO:0019730	antimicrobial humoral response	2023/2/14	122/18723	0.003640845	0.038012889	0.020721312	GNLY/S100A12	2
BP	GO:1990266	neutrophil migration	2023/2/14	122/18723	0.003640845	0.038012889	0.020721312	S100A12/S100A8	2
BP	GO:0071621	granulocyte chemotaxis	2023/2/14	125/18723	0.003817972	0.039197846	0.021367247	S100A12/S100A8	2
BP	GO:0050729	positive regulation of inflammatory response	2023/2/14	142/18723	0.004896195	0.049443543	0.026952307	S100A12/S100A8	2
BP	GO:0010821	regulation of mitochondrion organization	2023/2/14	144/18723	0.005031289	0.049988291	0.027249257	MMP9/GZMB	2
BP	GO:0097530	granulocyte migration	2023/2/14	148/18723	0.00530663	0.051887048	0.028284293	S100A12/S100A8	2
BP	GO:2001242	regulation of intrinsic apoptotic signaling pathway	2023/2/14	164/18723	0.006476013	0.062331623	0.033977764	MMP9/S100A8	2
BP	GO:0002887	negative regulation of myeloid leukocyte mediated immunity	2023/1/14	10/18723	0.00745411	0.063559494	0.034647092	CX3CR1	1
BP	GO:0032070	regulation of deoxyribonuclease activity	2023/1/14	10/18723	0.00745411	0.063559494	0.034647092	GZMA	1
BP	GO:0007369	gastrulation	2023/2/14	185/18723	0.008172921	0.063559494	0.034647092	MMP9/EOMES	2
BP	GO:0002357	defense response to tumor cell	2023/1/14	11/18723	0.008196676	0.063559494	0.034647092	PRF1	1
BP	GO:0002645	positive regulation of tolerance induction	2023/1/14	11/18723	0.008196676	0.063559494	0.034647092	CD3E	1
BP	GO:0002725	negative regulation of T cell cytokine production	2023/1/14	11/18723	0.008196676	0.063559494	0.034647092	TBX21	1
BP	GO:0033632	regulation of cell-cell adhesion mediated by integrin	2023/1/14	11/18723	0.008196676	0.063559494	0.034647092	CD3E	1
BP	GO:0045060	negative thymic T cell selection	2023/1/14	11/18723	0.008196676	0.063559494	0.034647092	CD3E	1
BP	GO:0071492	cellular response to UV-A	2023/1/14	11/18723	0.008196676	0.063559494	0.034647092	MMP9	1
BP	GO:0098883	synapse pruning	2023/1/14	11/18723	0.008196676	0.063559494	0.034647092	CX3CR1	1
BP	GO:1901030	positive regulation of mitochondrial outer membrane permeabilization involved in apoptotic signaling pathway	2023/1/14	11/18723	0.008196676	0.063559494	0.034647092	GZMB	1
BP	GO:2000320	negative regulation of T-helper 17 cell differentiation	2023/1/14	11/18723	0.008196676	0.063559494	0.034647092	TBX21	1
BP	GO:0002664	regulation of T cell tolerance induction	2023/1/14	12/18723	0.008938726	0.063559494	0.034647092	CD3E	1
BP	GO:0010454	negative regulation of cell fate commitment	2023/1/14	12/18723	0.008938726	0.063559494	0.034647092	TBX21	1
BP	GO:0043383	negative T cell selection	2023/1/14	12/18723	0.008938726	0.063559494	0.034647092	CD3E	1
BP	GO:0051549	positive regulation of keratinocyte migration	2023/1/14	12/18723	0.008938726	0.063559494	0.034647092	MMP9	1
BP	GO:2000317	negative regulation of T-helper 17 type immune response	2023/1/14	12/18723	0.008938726	0.063559494	0.034647092	TBX21	1
BP	GO:0002285	lymphocyte activation involved in immune response	2023/2/14	194/18723	0.008955311	0.063559494	0.034647092	TBX21/EOMES	2
BP	GO:0019722	calcium-mediated signaling	2023/2/14	202/18723	0.009678059	0.063559494	0.034647092	CX3CR1/CD3E	2
BP	GO:0035745	T-helper 2 cell cytokine production	2023/1/14	13/18723	0.009680261	0.063559494	0.034647092	TBX21	1
BP	GO:0048302	regulation of isotype switching to IgG isotypes	2023/1/14	13/18723	0.009680261	0.063559494	0.034647092	TBX21	1
BP	GO:0051709	regulation of killing of cells of other organism	2023/1/14	13/18723	0.009680261	0.063559494	0.034647092	PRF1	1
BP	GO:0070486	leukocyte aggregation	2023/1/14	13/18723	0.009680261	0.063559494	0.034647092	S100A8	1
BP	GO:1900272	negative regulation of long-term synaptic potentiation	2023/1/14	13/18723	0.009680261	0.063559494	0.034647092	CX3CR1	1
BP	GO:1903977	positive regulation of glial cell migration	2023/1/14	13/18723	0.009680261	0.063559494	0.034647092	CX3CR1	1
BP	GO:2000551	regulation of T-helper 2 cell cytokine production	2023/1/14	13/18723	0.009680261	0.063559494	0.034647092	TBX21	1
BP	GO:0043281	regulation of cysteine-type endopeptidase activity involved in apoptotic process	2023/2/14	209/18723	0.010331299	0.063559494	0.034647092	MMP9/S100A8	2
BP	GO:0001711	endodermal cell fate commitment	2023/1/14	14/18723	0.010421281	0.063559494	0.034647092	EOMES	1
BP	GO:0001771	immunological synapse formation	2023/1/14	14/18723	0.010421281	0.063559494	0.034647092	PRF1	1
BP	GO:0002517	T cell tolerance induction	2023/1/14	14/18723	0.010421281	0.063559494	0.034647092	CD3E	1
BP	GO:0002829	negative regulation of type 2 immune response	2023/1/14	14/18723	0.010421281	0.063559494	0.034647092	TBX21	1
BP	GO:0043374	CD8-positive, alpha-beta T cell differentiation	2023/1/14	14/18723	0.010421281	0.063559494	0.034647092	EOMES	1
BP	GO:0045059	positive thymic T cell selection	2023/1/14	14/18723	0.010421281	0.063559494	0.034647092	CD3E	1
BP	GO:0048291	isotype switching to IgG isotypes	2023/1/14	14/18723	0.010421281	0.063559494	0.034647092	TBX21	1
BP	GO:0051547	regulation of keratinocyte migration	2023/1/14	14/18723	0.010421281	0.063559494	0.034647092	MMP9	1
BP	GO:0070141	response to UV-A	2023/1/14	14/18723	0.010421281	0.063559494	0.034647092	MMP9	1
BP	GO:0072540	T-helper 17 cell lineage commitment	2023/1/14	14/18723	0.010421281	0.063559494	0.034647092	TBX21	1
BP	GO:0002274	myeloid leukocyte activation	2023/2/14	223/18723	0.01169533	0.067802351	0.036959928	S100A12/CX3CR1	2

BP	GO:2001234	negative regulation of apoptotic signaling pathway	2023/2/14	224/18723	0.011795667	0.067802351	0.036959928	MMP9/CX3CR1	2
BP	GO:0017014	protein nitrosylation	2023/1/14	16/18723	0.011901775	0.067802351	0.036959928	S100A8	1
BP	GO:0018119	peptidyl-cysteine S-nitrosylation	2023/1/14	16/18723	0.011901775	0.067802351	0.036959928	S100A8	1
BP	GO:0033631	cell-cell adhesion mediated by integrin	2023/1/14	16/18723	0.011901775	0.067802351	0.036959928	CD3E	1
BP	GO:0051238	sequestering of metal ion	2023/1/14	16/18723	0.011901775	0.067802351	0.036959928	S100A8	1
BP	GO:2001267	regulation of cysteine-type endopeptidase activity involved in apoptotic signaling pathway	2023/1/14	16/18723	0.011901775	0.067802351	0.036959928	MMP9	1
BP	GO:0002703	regulation of leukocyte mediated immunity	2023/2/14	226/18723	0.011997494	0.067802351	0.036959928	TBX21/CX3CR1	2
BP	GO:2000696	regulation of epithelial cell differentiation involved in kidney development	2023/1/14	17/18723	0.012641251	0.070791007	0.038589083	MMP9	1
BP	GO:2000116	regulation of cysteine-type endopeptidase activity	2023/2/14	235/18723	0.012924634	0.071053546	0.038732196	MMP9/S100A8	2
BP	GO:0002295	T-helper cell lineage commitment	2023/1/14	18/18723	0.013380213	0.071053546	0.038732196	TBX21	1
BP	GO:0002544	chronic inflammatory response	2023/1/14	18/18723	0.013380213	0.071053546	0.038732196	S100A8	1
BP	GO:0002643	regulation of tolerance induction	2023/1/14	18/18723	0.013380213	0.071053546	0.038732196	CD3E	1
BP	GO:0035743	CD4-positive, alpha-beta T cell cytokine production	2023/1/14	18/18723	0.013380213	0.071053546	0.038732196	TBX21	1
BP	GO:0045623	negative regulation of T-helper cell differentiation	2023/1/14	18/18723	0.013380213	0.071053546	0.038732196	TBX21	1
BP	GO:0002281	macrophage activation involved in immune response	2023/1/14	19/18723	0.014118662	0.071287668	0.038859819	CX3CR1	1
BP	GO:0002523	leukocyte migration involved in inflammatory response	2023/1/14	19/18723	0.014118662	0.071287668	0.038859819	S100A8	1
BP	GO:0051546	keratinocyte migration	2023/1/14	19/18723	0.014118662	0.071287668	0.038859819	MMP9	1
BP	GO:0061760	antifungal innate immune response	2023/1/14	19/18723	0.014118662	0.071287668	0.038859819	CX3CR1	1
BP	GO:1903975	regulation of glial cell migration	2023/1/14	19/18723	0.014118662	0.071287668	0.038859819	CX3CR1	1
BP	GO:2000319	regulation of T-helper 17 cell differentiation	2023/1/14	19/18723	0.014118662	0.071287668	0.038859819	TBX21	1
BP	GO:0045165	cell fate commitment	2023/2/14	258/18723	0.015432397	0.073891659	0.040279288	TBX21/EOMES	2
BP	GO:0007252	I-kappaB phosphorylation	2023/1/14	21/18723	0.015594019	0.073891659	0.040279288	CX3CR1	1
BP	GO:0033630	positive regulation of cell adhesion mediated by integrin	2023/1/14	21/18723	0.015594019	0.073891659	0.040279288	CD3E	1
BP	GO:0043373	CD4-positive, alpha-beta T cell lineage commitment	2023/1/14	21/18723	0.015594019	0.073891659	0.040279288	TBX21	1
BP	GO:0046641	positive regulation of alpha-beta T cell proliferation	2023/1/14	21/18723	0.015594019	0.073891659	0.040279288	CD3E	1
BP	GO:0090026	positive regulation of monocyte chemotaxis	2023/1/14	21/18723	0.015594019	0.073891659	0.040279288	CX3CR1	1
BP	GO:0150146	cell junction disassembly	2023/1/14	21/18723	0.015594019	0.073891659	0.040279288	CX3CR1	1
BP	GO:1901028	regulation of mitochondrial outer membrane permeabilization involved in apoptotic signaling pathway	2023/1/14	21/18723	0.015594019	0.073891659	0.040279288	GZMB	1
BP	GO:0032069	regulation of nuclease activity	2023/1/14	22/18723	0.016330929	0.075073522	0.040923537	GZMA	1
BP	GO:0043371	negative regulation of CD4-positive, alpha-beta T cell differentiation	2023/1/14	22/18723	0.016330929	0.075073522	0.040923537	TBX21	1
BP	GO:0045061	thymic T cell selection	2023/1/14	22/18723	0.016330929	0.075073522	0.040923537	CD3E	1
BP	GO:1900120	regulation of receptor binding	2023/1/14	22/18723	0.016330929	0.075073522	0.040923537	MMP9	1
BP	GO:0002052	positive regulation of neuroblast proliferation	2023/1/14	23/18723	0.017067326	0.076740677	0.041832324	CX3CR1	1
BP	GO:0002363	alpha-beta T cell lineage commitment	2023/1/14	23/18723	0.017067326	0.076740677	0.041832324	TBX21	1
BP	GO:0043369	CD4-positive or CD8-positive, alpha-beta T cell lineage commitment	2023/1/14	23/18723	0.017067326	0.076740677	0.041832324	TBX21	1
BP	GO:0031349	positive regulation of defense response	2023/2/14	278/18723	0.017770814	0.078757024	0.04293146	S100A12/S100A8	2
BP	GO:0001911	negative regulation of leukocyte mediated cytotoxicity	2023/1/14	24/18723	0.017803211	0.078757024	0.04293146	CX3CR1	1
BP	GO:0007249	I-kappaB kinase/NF-kappaB signaling	2023/2/14	281/18723	0.018133956	0.078757024	0.04293146	S100A12/CX3CR1	2
BP	GO:0032753	positive regulation of interleukin-4 production	2023/1/14	25/18723	0.018538585	0.078757024	0.04293146	CD3E	1
BP	GO:0051204	protein insertion into mitochondrial membrane	2023/1/14	25/18723	0.018538585	0.078757024	0.04293146	GZMB	1
BP	GO:0060074	synapse maturation	2023/1/14	25/18723	0.018538585	0.078757024	0.04293146	CX3CR1	1
BP	GO:0060706	cell differentiation involved in embryonic placenta development	2023/1/14	25/18723	0.018538585	0.078757024	0.04293146	EOMES	1
BP	GO:2000316	regulation of T-helper 17 type immune response	2023/1/14	25/18723	0.018538585	0.078757024	0.04293146	TBX21	1
BP	GO:0097193	intrinsic apoptotic signaling pathway	2023/2/14	288/18723	0.018993662	0.079149625	0.043145472	MMP9/S100A8	2
BP	GO:0002418	immune response to tumor cell	2023/1/14	26/18723	0.019273448	0.079149625	0.043145472	PRF1	1
BP	GO:0002710	negative regulation of T cell mediated immunity	2023/1/14	26/18723	0.019273448	0.079149625	0.043145472	TBX21	1
BP	GO:0046639	negative regulation of alpha-beta T cell differentiation	2023/1/14	26/18723	0.019273448	0.079149625	0.043145472	TBX21	1
BP	GO:1905523	positive regulation of macrophage migration	2023/1/14	26/18723	0.019273448	0.079149625	0.043145472	CX3CR1	1
BP	GO:0002719	negative regulation of cytokine production involved in immune response	2023/1/14	27/18723	0.020007799	0.080031197	0.043626029	TBX21	1
BP	GO:0032703	negative regulation of interleukin-2 production	2023/1/14	27/18723	0.020007799	0.080031197	0.043626029	TBX21	1
BP	GO:0036037	CD8-positive, alpha-beta T cell activation	2023/1/14	27/18723	0.020007799	0.080031197	0.043626029	EOMES	1
BP	GO:0045830	positive regulation of isotype switching	2023/1/14	27/18723	0.020007799	0.080031197	0.043626029	TBX21	1
BP	GO:0042063	gliogenesis	2023/2/14	301/18723	0.020635717	0.080866142	0.044081168	CX3CR1/S100A8	2
BP	GO:0002507	tolerance induction	2023/1/14	28/18723	0.02074164	0.080866142	0.044081168	CD3E	1
BP	GO:0031342	negative regulation of cell killing	2023/1/14	28/18723	0.02074164	0.080866142	0.044081168	CX3CR1	1
BP	GO:0090200	positive regulation of release of cytochrome c from mitochondria	2023/1/14	28/18723	0.02074164	0.080866142	0.044081168	MMP9	1
BP	GO:0002360	T cell lineage commitment	2023/1/14	29/18723	0.021474971	0.082165106	0.04478925	TBX21	1
BP	GO:0072539	T-helper 17 cell differentiation	2023/1/14	29/18723	0.021474971	0.082165106	0.04478925	TBX21	1
BP	GO:0090025	regulation of monocyte chemotaxis	2023/1/14	29/18723	0.021474971	0.082165106	0.04478925	CX3CR1	1
BP	GO:0019932	second-messenger-mediated signaling	2023/2/14	312/18723	0.022070609	0.083414632	0.045470382	CX3CR1/CD3E	2
BP	GO:0010453	regulation of cell fate commitment	2023/1/14	30/18723	0.022207792	0.083414632	0.045470382	TBX21	1
BP	GO:0090151	establishment of protein localization to mitochondrial membrane	2023/1/14	30/18723	0.022207792	0.083414632	0.045470382	GZMB	1
BP	GO:0006959	humoral immune response	2023/2/14	317/18723	0.022736413	0.084617385	0.046126018	GNLY/S100A12	2
BP	GO:0002828	regulation of type 2 immune response	2023/1/14	31/18723	0.022940103	0.084617385	0.046126018	TBX21	1
BP	GO:0060795	cell fate commitment involved in formation of primary germ layer	2023/1/14	31/18723	0.022940103	0.084617385	0.046126018	EOMES	1
BP	GO:0045879	negative regulation of smoothened signaling pathway	2023/1/14	32/18723	0.023671905	0.085274229	0.046484072	CD3E	1
BP	GO:0050850	positive regulation of calcium-mediated signaling	2023/1/14	32/18723	0.023671905	0.085274229	0.046484072	CD3E	1
BP	GO:0097345	mitochondrial outer membrane permeabilization	2023/1/14	32/18723	0.023671905	0.085274229	0.046484072	GZMB	1
BP	GO:2000515	negative regulation of CD4-positive, alpha-beta T cell activation	2023/1/14	32/18723	0.023671905	0.085274229	0.046484072	TBX21	1
BP	GO:0050863	regulation of T cell activation	2023/2/14	329/18723	0.024368496	0.085411191	0.046558732	TBX21/CD3E	2
BP	GO:0032633	interleukin-4 production	2023/1/14	33/18723	0.024403197	0.085411191	0.046558732	CD3E	1
BP	GO:0032673	regulation of interleukin-4 production	2023/1/14	33/18723	0.024403197	0.085411191	0.046558732	CD3E	1
BP	GO:0050901	leukocyte tethering or rolling	2023/1/14	33/18723	0.024403197	0.085411191	0.046558732	CX3CR1	1
BP	GO:1902692	regulation of neuroblast proliferation	2023/1/14	33/18723	0.024403197	0.085411191	0.046558732	CX3CR1	1
BP	GO:0032743	positive regulation of interleukin-2 production	2023/1/14	34/18723	0.025133981	0.087471935	0.04768207	CD3E	1
BP	GO:1903037	regulation of leukocyte cell-cell adhesion	2023/2/14	336/18723	0.025342546	0.087702295	0.047807642	TBX21/CD3E	2
BP	GO:0002697	regulation of immune effector process	2023/2/14	339/18723	0.025764905	0.087811314	0.04786707	TBX21/CX3CR1	2
BP	GO:0046640	regulation of alpha-beta T cell proliferation	2023/1/14	35/18723	0.025864257	0.087811314	0.04786707	CD3E	1
BP	GO:0032496	response to lipopolysaccharide	2023/2/14	343/18723	0.026332599	0.087811314	0.04786707	CX3CR1/S100A8	2
BP	GO:0042092	type 2 immune response	2023/1/14	36/18723	0.026594025	0.087811314	0.04786707	TBX21	1
BP	GO:0045191	regulation of isotype switching	2023/1/14	36/18723	0.026594025	0.087811314	0.04786707	TBX21	1
BP	GO:0045742	positive regulation of epidermal growth factor receptor signaling pathway	2023/1/14	36/18723	0.026594025	0.087811314	0.04786707	MMP9	1
BP	GO:0051354	negative regulation of oxidoreductase activity	2023/1/14	36/18723	0.026594025	0.087811314	0.04786707	GZMA	1
BP	GO:1903749	positive regulation of establishment of protein localization to mitochondrion	2023/1/14	36/18723	0.026594025	0.087811314	0.04786707	GZMB	1
BP	GO:0002369	T cell cytokine production	2023/1/14	37/18723	0.027323285	0.087811314	0.04786707	TBX21	1
BP	GO:0002724	regulation of T cell cytokine production	2023/1/14	37/18723	0.027323285	0.087811314	0.04786707	TBX21	1
BP	GO:0006882	cellular zinc ion homeostasis	2023/1/14	38/18723	0.028052038	0.087811314	0.04786707	S100A8	1
BP	GO:0046633	alpha-beta T cell proliferation	2023/1/14	38/18723	0.028052038	0.087811314	0.04786707	CD3E	1
BP	GO:0072538	T-helper 17 type immune response	2023/1/14	38/18723	0.028052038	0.087811314	0.04786707	TBX21	1
BP	GO:1901186	positive regulation of ERBB signaling pathway	2023/1/14	38/18723	0.028052038	0.087811314	0.04786707	MMP9	1
BP	GO:1902110	positive regulation of mitochondrial membrane permeability involved in apoptotic process	2023/1/14	38/18723	0.028052038	0.087811314	0.04786707	GZMB	1
BP	GO:0001818	negative regulation of cytokine production	2023/2/14	357/18723	0.028360042	0.087811314	0.04786707	TBX21/CX3CR1	2
BP	GO:0051052	regulation of DNA metabolic process	2023/2/14	359/18723	0.028654769	0.087811314	0.04786707	TBX21/GZMA	2
BP	GO:0002347	response to tumor cell	2023/1/14	39/18723	0.028780284	0.087811314	0.04786707	PRF1	1
BP	GO:0002701	negative regulation of production of molecular mediator of immune response	2023/1/14	39/18723	0.028780284	0.087811314	0.04786707	TBX21	1
BP	GO:0045622	regulation of T-helper cell differentiation	2023/1/14	39/18723	0.028780284	0.087811314	0.04786707	TBX21	1
BP	GO:0051251	positive regulation of lymphocyte activation	2023/2/14	362/18723	0.029099225	0.087811314	0.04786707	TBX21/CD3E	2
BP	GO:0002237	response to molecule of bacterial origin	2023/2/14	363/18723	0.029248005	0.087811314	0.04786707	CX3CR1/S100A8	2
BP	GO:0051098	regulation of binding	2023/2/14	363/18723	0.029248005	0.087811314	0.04786707	MMP9/GZMA	2
BP	GO:0002714	positive regulation of B cell mediated immunity	2023/1/14	40/18723	0.029508023	0.087811314	0.04786707	TBX21	1
BP	GO:0002891	positive regulation of immunoglobulin mediated immune response	2023/1/14	40/18723	0.029508023	0.087811314	0.04786707	TBX21	1
BP	GO:0055069	zinc ion homeostasis	2023/1/14	40/18723	0.029508023	0.087811314	0.04786707	S100A8	1

BP	GO:0071276	cellular response to cadmium ion	2023/1/14	40/18723	0.029508023	0.087811314	0.04786707	MMP9	1
BP	GO:0150077	regulation of neuroinflammatory response	2023/1/14	40/18723	0.029508023	0.087811314	0.04786707	MMP9	1
BP	GO:1902686	mitochondrial outer membrane permeabilization involved in programmed cell death	2023/1/14	40/18723	0.029508023	0.087811314	0.04786707	GZMB	1
BP	GO:0009615	response to virus	2023/2/14	367/18723	0.029846257	0.088390838	0.048182976	TBX21/PRF1	2
BP	GO:1905521	regulation of macrophage migration	2023/1/14	41/18723	0.030235255	0.089114437	0.048577418	CX3CR1	1
BP	GO:0010038	response to metal ion	2023/2/14	373/18723	0.030752972	0.089965004	0.049041074	MMP9/S100A8	2
BP	GO:0030574	collagen catabolic process	2023/1/14	42/18723	0.030961982	0.089965004	0.049041074	MMP9	1
BP	GO:0032691	negative regulation of interleukin-1 beta production	2023/1/14	42/18723	0.030961982	0.089965004	0.049041074	CX3CR1	1
BP	GO:0051346	negative regulation of hydrolase activity	2023/2/14	379/18723	0.031670801	0.09037006	0.049261875	MMP9/GZMA	2
BP	GO:0014002	astrocyte development	2023/1/14	43/18723	0.031688203	0.09037006	0.049261875	S100A8	1
BP	GO:0035794	positive regulation of mitochondrial membrane permeability	2023/1/14	43/18723	0.031688203	0.09037006	0.049261875	GZMB	1
BP	GO:0046636	negative regulation of alpha-beta T cell activation	2023/1/14	43/18723	0.031688203	0.09037006	0.049261875	TBX21	1
BP	GO:0031295	T cell costimulation	2023/1/14	44/18723	0.032413918	0.091591622	0.049927764	CD3E	1
BP	GO:0150076	neuroinflammatory response	2023/1/14	44/18723	0.032413918	0.091591622	0.049927764	MMP9	1
BP	GO:1902108	regulation of mitochondrial membrane permeability involved in apoptotic process	2023/1/14	45/18723	0.033139129	0.093213257	0.050811738	GZMB	1
BP	GO:0031294	lymphocyte costimulation	2023/1/14	46/18723	0.033863834	0.093660398	0.05105548	CD3E	1
BP	GO:1900271	regulation of long-term synaptic potentiation	2023/1/14	46/18723	0.033863834	0.093660398	0.05105548	CX3CR1	1
BP	GO:0001774	microglial cell activation	2023/1/14	47/18723	0.034588036	0.093660398	0.05105548	CX3CR1	1
BP	GO:0035850	epithelial cell differentiation involved in kidney development	2023/1/14	47/18723	0.034588036	0.093660398	0.05105548	MMP9	1
BP	GO:0045581	negative regulation of T cell differentiation	2023/1/14	47/18723	0.034588036	0.093660398	0.05105548	TBX21	1
BP	GO:0045911	positive regulation of DNA recombination	2023/1/14	47/18723	0.034588036	0.093660398	0.05105548	TBX21	1
BP	GO:0033628	regulation of cell adhesion mediated by integrin	2023/1/14	48/18723	0.035311733	0.093660398	0.05105548	CD3E	1
BP	GO:0090199	regulation of release of cytochrome c from mitochondria	2023/1/14	48/18723	0.035311733	0.093660398	0.05105548	MMP9	1
BP	GO:1905710	positive regulation of membrane permeability	2023/1/14	48/18723	0.035311733	0.093660398	0.05105548	GZMB	1
BP	GO:0001913	T cell mediated cytotoxicity	2023/1/14	49/18723	0.036034926	0.093660398	0.05105548	PRF1	1
BP	GO:0002204	somatic recombination of immunoglobulin genes involved in immune response	2023/1/14	49/18723	0.036034926	0.093660398	0.05105548	TBX21	1
BP	GO:0002208	somatic diversification of immunoglobulins involved in immune response	2023/1/14	49/18723	0.036034926	0.093660398	0.05105548	TBX21	1
BP	GO:0018198	peptidyl-cysteine modification	2023/1/14	49/18723	0.036034926	0.093660398	0.05105548	S100A8	1
BP	GO:0030225	macrophage differentiation	2023/1/14	49/18723	0.036034926	0.093660398	0.05105548	MMP9	1
BP	GO:0030857	negative regulation of epithelial cell differentiation	2023/1/14	49/18723	0.036034926	0.093660398	0.05105548	MMP9	1
BP	GO:0032692	negative regulation of interleukin-1 production	2023/1/14	49/18723	0.036034926	0.093660398	0.05105548	CX3CR1	1
BP	GO:0045190	isotype switching	2023/1/14	49/18723	0.036034926	0.093660398	0.05105548	TBX21	1
BP	GO:1904707	positive regulation of vascular associated smooth muscle cell proliferation	2023/1/14	49/18723	0.036034926	0.093660398	0.05105548	MMP9	1
BP	GO:0002696	positive regulation of leukocyte activation	2023/2/14	409/18723	0.0364231	0.094271553	0.051388628	TBX21/CD3E	2
BP	GO:0002639	positive regulation of immunoglobulin production	2023/1/14	50/18723	0.036757616	0.094344547	0.051428419	TBX21	1
BP	GO:1903747	regulation of establishment of protein localization to mitochondrion	2023/1/14	50/18723	0.036757616	0.094344547	0.051428419	GZMB	1
BP	GO:0043370	regulation of CD4-positive, alpha-beta T cell differentiation	2023/1/14	51/18723	0.037479803	0.095403134	0.052005468	TBX21	1
BP	GO:0048013	ephrin receptor signaling pathway	2023/1/14	51/18723	0.037479803	0.095403134	0.052005468	MMP9	1
BP	GO:0035176	social behavior	2023/1/14	52/18723	0.038201487	0.096126422	0.052399741	CX3CR1	1
BP	GO:0043392	negative regulation of DNA binding	2023/1/14	52/18723	0.038201487	0.096126422	0.052399741	GZMA	1
BP	GO:0050867	positive regulation of cell activation	2023/2/14	420/18723	0.0382321	0.096126422	0.052399741	TBX21/CD3E	2
BP	GO:0002707	negative regulation of lymphocyte mediated immunity	2023/1/14	53/18723	0.038922668	0.096678885	0.052700896	TBX21	1
BP	GO:0007566	embryo implantation	2023/1/14	53/18723	0.038922668	0.096678885	0.052700896	MMP9	1
BP	GO:0008347	glial cell migration	2023/1/14	53/18723	0.038922668	0.096678885	0.052700896	CX3CR1	1
BP	GO:0002823	negative regulation of adaptive immune response based on somatic recombination of immune receptors built from immunoglobulin superfamily domains	2023/1/14	54/18723	0.039643347	0.09729204	0.053035134	TBX21	1
BP	GO:0051703	biological process involved in intraspecies interaction between organisms	2023/1/14	54/18723	0.039643347	0.09729204	0.053035134	CX3CR1	1
BP	GO:2000179	positive regulation of neural precursor cell proliferation	2023/1/14	54/18723	0.039643347	0.09729204	0.053035134	CX3CR1	1
BP	GO:0052548	regulation of endopeptidase activity	2023/2/14	432/18723	0.040245127	0.097734949	0.05327657	MMP9/S100A8	2
BP	GO:0045620	negative regulation of lymphocyte differentiation	2023/1/14	55/18723	0.040363525	0.097734949	0.05327657	TBX21	1
BP	GO:1905517	macrophage migration	2023/1/14	55/18723	0.040363525	0.097734949	0.05327657	CX3CR1	1
BP	GO:0002683	negative regulation of immune system process	2023/2/14	434/18723	0.040584594	0.097734949	0.05327657	TBX21/CX3CR1	2
BP	GO:0002886	regulation of myeloid leukocyte mediated immunity	2023/1/14	56/18723	0.041083201	0.097734949	0.05327657	CX3CR1	1
BP	GO:0043388	positive regulation of DNA binding	2023/1/14	56/18723	0.041083201	0.097734949	0.05327657	MMP9	1
BP	GO:1904645	response to amyloid-beta	2023/1/14	56/18723	0.041083201	0.097734949	0.05327657	MMP9	1
BP	GO:0016447	somatic recombination of immunoglobulin gene segments	2023/1/14	57/18723	0.041802376	0.097734949	0.05327657	TBX21	1
BP	GO:0051205	protein insertion into membrane	2023/1/14	57/18723	0.041802376	0.097734949	0.05327657	GZMB	1
BP	GO:0061005	cell differentiation involved in kidney development	2023/1/14	57/18723	0.041802376	0.097734949	0.05327657	MMP9	1
BP	GO:0061756	leukocyte adhesion to vascular endothelial cell	2023/1/14	57/18723	0.041802376	0.097734949	0.05327657	CX3CR1	1
BP	GO:0022411	cellular component disassembly	2023/2/14	443/18723	0.042126032	0.097734949	0.05327657	MMP9/CX3CR1	2
BP	GO:0001954	positive regulation of cell-matrix adhesion	2023/1/14	58/18723	0.042521049	0.097734949	0.05327657	CD3E	1
BP	GO:0002931	response to ischemia	2023/1/14	58/18723	0.042521049	0.097734949	0.05327657	CX3CR1	1
BP	GO:0007405	neuroblast proliferation	2023/1/14	58/18723	0.042521049	0.097734949	0.05327657	CX3CR1	1
BP	GO:0010043	response to zinc ion	2023/1/14	58/18723	0.042521049	0.097734949	0.05327657	S100A8	1
BP	GO:2001244	positive regulation of intrinsic apoptotic signaling pathway	2023/1/14	58/18723	0.042521049	0.097734949	0.05327657	S100A8	1
BP	GO:0022407	regulation of cell-cell adhesion	2023/2/14	448/18723	0.042992087	0.097924122	0.053379691	TBX21/CD3E	2
BP	GO:0001836	release of cytochrome c from mitochondria	2023/1/14	59/18723	0.043239223	0.097924122	0.053379691	MMP9	1
BP	GO:0002820	negative regulation of adaptive immune response	2023/1/14	59/18723	0.043239223	0.097924122	0.053379691	TBX21	1
BP	GO:2001258	negative regulation of cation channel activity	2023/1/14	59/18723	0.043239223	0.097924122	0.053379691	MMP9	1
BP	GO:0002712	regulation of B cell mediated immunity	2023/1/14	60/18723	0.043956896	0.098463447	0.053673683	TBX21	1
BP	GO:0002889	regulation of immunoglobulin mediated immune response	2023/1/14	60/18723	0.043956896	0.098463447	0.053673683	TBX21	1
BP	GO:0051851	modulation by host of symbiont process	2023/1/14	60/18723	0.043956896	0.098463447	0.053673683	CX3CR1	1
BP	GO:0052547	regulation of peptidase activity	2023/2/14	461/18723	0.045275797	0.100578049	0.05482638	MMP9/S100A8	2
BP	GO:0045428	regulation of nitric oxide biosynthetic process	2023/1/14	62/18723	0.045390743	0.100578049	0.05482638	CX3CR1	1
BP	GO:0045576	mast cell activation	2023/1/14	62/18723	0.045390743	0.100578049	0.05482638	S100A12	1
BP	GO:0022617	extracellular matrix disassembly	2023/1/14	63/18723	0.046106917	0.101074238	0.055096859	MMP9	1
BP	GO:0031343	positive regulation of cell killing	2023/1/14	63/18723	0.046106917	0.101074238	0.055096859	PRF1	1
BP	GO:0046902	regulation of mitochondrial membrane permeability	2023/1/14	63/18723	0.046106917	0.101074238	0.055096859	GZMB	1
BP	GO:0080164	regulation of nitric oxide metabolic process	2023/1/14	64/18723	0.046822592	0.102279138	0.055753666	CX3CR1	1
BP	GO:0019221	cytokine-mediated signaling pathway	2023/2/14	472/18723	0.04724367	0.102834277	0.056056279	IL2RB/CX3CR1	2
BP	GO:0071677	positive regulation of mononuclear cell migration	2023/1/14	65/18723	0.047537769	0.103110091	0.056206629	CX3CR1	1
BP	GO:0002562	somatic diversification of immune receptors via germline recombination within a single locus	2023/1/14	66/18723	0.048252448	0.103206624	0.05625925	TBX21	1
BP	GO:0016444	somatic cell DNA recombination	2023/1/14	66/18723	0.048252448	0.103206624	0.05625925	TBX21	1
BP	GO:0042093	T-helper cell differentiation	2023/1/14	66/18723	0.048252448	0.103206624	0.05625925	TBX21	1
BP	GO:0050766	positive regulation of phagocytosis	2023/1/14	66/18723	0.048252448	0.103206624	0.05625925	IL2RB	1
BP	GO:0016445	somatic diversification of immunoglobulins	2023/1/14	67/18723	0.048966628	0.103277154	0.056297697	TBX21	1
BP	GO:0046635	positive regulation of alpha-beta T cell activation	2023/1/14	67/18723	0.048966628	0.103277154	0.056297697	CD3E	1
BP	GO:2000514	regulation of CD4-positive, alpha-beta T cell activation	2023/1/14	67/18723	0.048966628	0.103277154	0.056297697	TBX21	1
BP	GO:0002294	CD4-positive, alpha-beta T cell differentiation involved in immune response	2023/1/14	68/18723	0.049680311	0.103277154	0.056297697	TBX21	1
BP	GO:0009988	cell-cell recognition	2023/1/14	68/18723	0.049680311	0.103277154	0.056297697	PRF1	1
BP	GO:0042267	natural killer cell mediated cytotoxicity	2023/1/14	68/18723	0.049680311	0.103277154	0.056297697	GZMB	1
BP	GO:0046637	regulation of alpha-beta T cell differentiation	2023/1/14	68/18723	0.049680311	0.103277154	0.056297697	TBX21	1
BP	GO:0046686	response to cadmium ion	2023/1/14	68/18723	0.049680311	0.103277154	0.056297697	MMP9	1
BP	GO:0072503	cellular divalent inorganic cation homeostasis	2023/2/14	486/18723	0.049794342	0.103277154	0.056297697	CX3CR1/S100A8	2
CC	GO:0001772	immunological synapse	2023/3/15	44/19550	4.75E-06	0.000118726	6.00E-05	GZMB/GZMA/CD3E	3
CC	GO:0009897	external side of plasma membrane	2023/4/15	421/19550	0.000239689	0.002996109	0.001513823	IL2RB/CX3CR1/CD8A/CD3E	4
CC	GO:0098802	plasma membrane signaling receptor complex	2023/3/15	306/19550	0.001502597	0.012521638	0.006326722	IL2RB/CD8A/CD3E	3
CC	GO:0042101	T cell receptor complex	2023/2/15	148/19550	0.005602929	0.035018307	0.017693461	CD8A/CD3E	2
CC	GO:0071682	endocytic vesicle lumen	2023/1/15	23/19550	0.017508687	0.060294687	0.030464684	GNLY	1
CC	GO:0032809	neuronal cell body membrane	2023/1/15	28/19550	0.021276867	0.060294687	0.030464684	CX3CR1	1
CC	GO:0044298	cell body membrane	2023/1/15	31/19550	0.023531296	0.060294687	0.030464684	CX3CR1	1

CC	GO:0034774	secretory granule lumen	2023/2/15	322/19550	0.02465081	0.060294687	0.030464684	S100A12/S100A8	2
CC	GO:0060205	cytoplasmic vesicle lumen	2023/2/15	325/19550	0.02507982	0.060294687	0.030464684	S100A12/S100A8	2
CC	GO:0031983	vesicle lumen	2023/2/15	327/19550	0.025367554	0.060294687	0.030464684	S100A12/S100A8	2
CC	GO:0031904	endosome lumen	2023/1/15	35/19550	0.026529662	0.060294687	0.030464684	PRF1	1
CC	GO:0062023	collagen-containing extracellular matrix	2023/2/15	425/19550	0.041070591	0.079601978	0.040219947	MMP9/S100A8	2
CC	GO:1904724	tertiary granule lumen	2023/1/15	55/19550	0.041393029	0.079601978	0.040219947	MMP9	1
MF	GO:0004252	serine-type endopeptidase activity	2023/4/15	174/18368	9.79E-06	0.000255691	0.000134574	MMP9/GZMH/GZMB/GZMA	4
MF	GO:0008236	serine-type peptidase activity	2023/4/15	191/18368	1.41E-05	0.000255691	0.000134574	MMP9/GZMH/GZMB/GZMA	4
MF	GO:0017171	serine hydrolase activity	2023/4/15	195/18368	1.53E-05	0.000255691	0.000134574	MMP9/GZMH/GZMB/GZMA	4
MF	GO:0050786	RAGE receptor binding	2023/2/15	10/18368	2.79E-05	0.00034882	0.00018359	S100A12/S100A8	2
MF	GO:0004175	endopeptidase activity	2023/4/15	430/18368	0.000329363	0.003293633	0.001733491	MMP9/GZMH/GZMB/GZMA	4
MF	GO:0048306	calcium-dependent protein binding	2023/2/15	88/18368	0.002288107	0.019067554	0.010035555	S100A12/S100A8	2
MF	GO:0004896	cytokine receptor activity	2023/2/15	97/18368	0.002771249	0.019794635	0.010418229	IL2RB/CX3CR1	2
MF	GO:0019955	cytokine binding	2023/2/15	139/18368	0.00559675	0.033300123	0.017526381	IL2RB/CX3CR1	2
MF	GO:0140375	immune receptor activity	2023/2/15	144/18368	0.005994022	0.033300123	0.017526381	IL2RB/CX3CR1	2
MF	GO:0042608	T cell receptor binding	2023/1/15	11/18368	0.008948851	0.044357569	0.023346089	CD3E	1
MF	GO:0035325	Toll-like receptor binding	2023/1/15	12/18368	0.009758665	0.044357569	0.023346089	S100A8	1
MF	GO:0036041	long-chain fatty acid binding	2023/1/15	14/18368	0.011376442	0.047401842	0.024948338	S100A8	1
MF	GO:0042288	MHC class I protein binding	2023/1/15	20/18368	0.016214984	0.061857108	0.032556372	CD8A	1
MF	GO:0016493	C-C chemokine receptor activity	2023/1/15	23/18368	0.018625958	0.061857108	0.032556372	CX3CR1	1
MF	GO:0019957	C-C chemokine binding	2023/1/15	24/18368	0.01942839	0.061857108	0.032556372	CX3CR1	1
MF	GO:0001637	G protein-coupled chemoattractant receptor activity	2023/1/15	26/18368	0.021031417	0.061857108	0.032556372	CX3CR1	1
MF	GO:0004950	chemokine receptor activity	2023/1/15	26/18368	0.021031417	0.061857108	0.032556372	CX3CR1	1
MF	GO:0022829	wide pore channel activity	2023/1/15	30/18368	0.024230135	0.06730593	0.035424174	PRF1	1
MF	GO:0019956	chemokine binding	2023/1/15	33/18368	0.026622769	0.070059919	0.036873642	CX3CR1	1
MF	GO:0023023	MHC protein complex binding	2023/1/15	36/18368	0.029009927	0.072524817	0.038170956	CD8A	1
MF	GO:0005504	fatty acid binding	2023/1/15	39/18368	0.03139162	0.073146147	0.038497972	S100A8	1
MF	GO:0042287	MHC protein binding	2023/1/15	40/18368	0.032184305	0.073146147	0.038497972	CD8A	1
MF	GO:0015026	coreceptor activity	2023/1/15	48/18368	0.038504024	0.083495676	0.043945092	CD8A	1
MF	GO:0030159	signaling receptor complex adaptor activity	2023/1/15	50/18368	0.040077924	0.083495676	0.043945092	CD3E	1
MF	GO:0005507	copper ion binding	2023/1/15	61/18368	0.048691481	0.097382963	0.051254191	S100A12	1
