# Supplementary material for: Integrated bioinformatics analysis of the effects of chronic pain on patients with spinal cord injury
Source: Front Cell Neurosci. 2025 Feb 5;19:1457740. doi: 10.3389/fncel.2025.1457740 (PMC11835904; doi:10.3389/fncel.2025.1457740)
Supplement: Supplementary Table S3 — Clinical information of patients enlisted in current study. [file Data_Sheet_5.pdf]

GSE151371		
GSM4576308	cell type: white blood cells	disease state: healthy control
GSM4576309	cell type: white blood cells	disease state: healthy control
GSM4576310	cell type: white blood cells	disease state: healthy control
GSM4576311	cell type: white blood cells	disease state: healthy control
GSM4576312	cell type: white blood cells	disease state: healthy control
GSM4576313	cell type: white blood cells	disease state: healthy control
GSM4576314	cell type: white blood cells	disease state: healthy control
GSM4576315	cell type: white blood cells	disease state: healthy control
GSM4576316	cell type: white blood cells	disease state: healthy control
GSM4576317	cell type: white blood cells	disease state: healthy control
GSM4576328	cell type: white blood cells	disease state: spinal cord injury
GSM4576329	cell type: white blood cells	disease state: spinal cord injury
GSM4576330	cell type: white blood cells	disease state: spinal cord injury
GSM4576331	cell type: white blood cells	disease state: spinal cord injury
GSM4576332	cell type: white blood cells	disease state: spinal cord injury
GSM4576333	cell type: white blood cells	disease state: spinal cord injury
GSM4576334	cell type: white blood cells	disease state: spinal cord injury
GSM4576335	cell type: white blood cells	disease state: spinal cord injury
GSM4576336	cell type: white blood cells	disease state: spinal cord injury
GSM4576337	cell type: white blood cells	disease state: spinal cord injury
GSM4576338	cell type: white blood cells	disease state: spinal cord injury
GSM4576339	cell type: white blood cells	disease state: spinal cord injury
GSM4576340	cell type: white blood cells	disease state: spinal cord injury
GSM4576341	cell type: white blood cells	disease state: spinal cord injury
GSM4576342	cell type: white blood cells	disease state: spinal cord injury
GSM4576343	cell type: white blood cells	disease state: spinal cord injury
GSM4576344	cell type: white blood cells	disease state: spinal cord injury
GSM4576345	cell type: white blood cells	disease state: spinal cord injury
GSM4576346	cell type: white blood cells	disease state: spinal cord injury
GSM4576347	cell type: white blood cells	disease state: spinal cord injury
GSM4576348	cell type: white blood cells	disease state: spinal cord injury
GSM4576349	cell type: white blood cells	disease state: spinal cord injury
GSM4576350	cell type: white blood cells	disease state: spinal cord injury
GSM4576351	cell type: white blood cells	disease state: spinal cord injury
GSM4576352	cell type: white blood cells	disease state: spinal cord injury
GSM4576353	cell type: white blood cells	disease state: spinal cord injury
GSM4576354	cell type: white blood cells	disease state: spinal cord injury
GSM4576355	cell type: white blood cells	disease state: spinal cord injury
GSM4576356	cell type: white blood cells	disease state: spinal cord injury
GSM4576357	cell type: white blood cells	disease state: spinal cord injury
GSM4576358	cell type: white blood cells	disease state: spinal cord injury
GSM4576359	cell type: white blood cells	disease state: spinal cord injury
GSM4576360	cell type: white blood cells	disease state: spinal cord injury
GSM4576361	cell type: white blood cells	disease state: spinal cord injury
GSM4576362	cell type: white blood cells	disease state: spinal cord injury
GSM4576363	cell type: white blood cells	disease state: spinal cord injury
GSM4576364	cell type: white blood cells	disease state: spinal cord injury
GSM4576365	cell type: white blood cells	disease state: spinal cord injury

GSE177034			
GSM5370696	Whole peripheral blood	paingroup: Resolved	type_chronic pain
GSM5370697	Whole peripheral blood	paingroup: Resolved	type_control
GSM5370698	Whole peripheral blood	paingroup: Resolved	type_chronic pain
GSM5370699	Whole peripheral blood	paingroup: Resolved	type_control
GSM5370700	Whole peripheral blood	paingroup: Resolved	type_chronic pain
GSM5370701	Whole peripheral blood	paingroup: Resolved	type_control
GSM5370702	Whole peripheral blood	paingroup: Resolved	type_chronic pain
GSM5370703	Whole peripheral blood	paingroup: Resolved	type_control
GSM5370706	Whole peripheral blood	paingroup: Resolved	type_chronic pain
GSM5370707	Whole peripheral blood	paingroup: Resolved	type_control
GSM5370714	Whole peripheral blood	paingroup: Resolved	type_chronic pain
GSM5370715	Whole peripheral blood	paingroup: Resolved	type_control
GSM5370722	Whole peripheral blood	paingroup: Resolved	type_chronic pain
GSM5370723	Whole peripheral blood	paingroup: Resolved	type_control
GSM5370726	Whole peripheral blood	paingroup: Resolved	type_chronic pain
GSM5370727	Whole peripheral blood	paingroup: Resolved	type_control
GSM5370732	Whole peripheral blood	paingroup: Resolved	type_chronic pain
GSM5370733	Whole peripheral blood	paingroup: Resolved	type_control
GSM5370738	Whole peripheral blood	paingroup: Resolved	type_chronic pain
GSM5370739	Whole peripheral blood	paingroup: Resolved	type_control
GSM5370740	Whole peripheral blood	paingroup: Resolved	type_chronic pain
GSM5370741	Whole peripheral blood	paingroup: Resolved	type_control
GSM5370742	Whole peripheral blood	paingroup: Resolved	type_chronic pain
GSM5370743	Whole peripheral blood	paingroup: Resolved	type_control
GSM5370748	Whole peripheral blood	paingroup: Resolved	type_chronic pain
GSM5370749	Whole peripheral blood	paingroup: Resolved	type_control
GSM5370752	Whole peripheral blood	paingroup: Resolved	type_chronic pain
GSM5370753	Whole peripheral blood	paingroup: Resolved	type_control
GSM5370760	Whole peripheral blood	paingroup: Resolved	type_chronic pain
GSM5370761	Whole peripheral blood	paingroup: Resolved	type_control
GSM5370762	Whole peripheral blood	paingroup: Resolved	type_chronic pain
GSM5370763	Whole peripheral blood	paingroup: Resolved	type_control
GSM5370770	Whole peripheral blood	paingroup: Resolved	type_chronic pain
GSM5370771	Whole peripheral blood	paingroup: Resolved	type_control
GSM5370772	Whole peripheral blood	paingroup: Resolved	type_chronic pain
GSM5370773	Whole peripheral blood	paingroup: Resolved	type_control
GSM5370776	Whole peripheral blood	paingroup: Resolved	type_chronic pain
GSM5370777	Whole peripheral blood	paingroup: Resolved	type_control
GSM5370780	Whole peripheral blood	paingroup: Resolved	type_chronic pain
GSM5370781	Whole peripheral blood	paingroup: Resolved	type_control
GSM5370782	Whole peripheral blood	paingroup: Resolved	type_chronic pain
GSM5370783	Whole peripheral blood	paingroup: Resolved	type_control
GSM5370784	Whole peripheral blood	paingroup: Resolved	type_chronic pain
GSM5370785	Whole peripheral blood	paingroup: Resolved	type_control
GSM5370792	Whole peripheral blood	paingroup: Resolved	type_chronic pain
GSM5370793	Whole peripheral blood	paingroup: Resolved	type_control
GSM5370794	Whole peripheral blood	paingroup: Resolved	type_chronic pain
GSM5370795	Whole peripheral blood	paingroup: Resolved	type_control
GSM5370800	Whole peripheral blood	paingroup: Resolved	type_chronic pain

GSM5370801	Whole peripheral blood	paingroup: Resolved	type_control
GSM5370802	Whole peripheral blood	paingroup: Resolved	type_chronic pain
GSM5370803	Whole peripheral blood	paingroup: Resolved	type_control
GSM5370804	Whole peripheral blood	paingroup: Resolved	type_chronic pain
GSM5370805	Whole peripheral blood	paingroup: Resolved	type_control
GSM5370806	Whole peripheral blood	paingroup: Resolved	type_chronic pain
GSM5370807	Whole peripheral blood	paingroup: Resolved	type_control
GSM5370810	Whole peripheral blood	paingroup: Resolved	type_chronic pain
GSM5370811	Whole peripheral blood	paingroup: Resolved	type_control
GSM5370812	Whole peripheral blood	paingroup: Resolved	type_chronic pain
GSM5370813	Whole peripheral blood	paingroup: Resolved	type_control
GSM5370814	Whole peripheral blood	paingroup: Resolved	type_chronic pain
GSM5370815	Whole peripheral blood	paingroup: Resolved	type_control
GSM5370816	Whole peripheral blood	paingroup: Resolved	type_chronic pain
GSM5370817	Whole peripheral blood	paingroup: Resolved	type_control
GSM5370818	Whole peripheral blood	paingroup: Resolved	type_chronic pain
GSM5370819	Whole peripheral blood	paingroup: Resolved	type_control
GSM5370820	Whole peripheral blood	paingroup: Resolved	type_chronic pain
GSM5370821	Whole peripheral blood	paingroup: Resolved	type_control
GSM5370822	Whole peripheral blood	paingroup: Resolved	type_chronic pain
GSM5370823	Whole peripheral blood	paingroup: Resolved	type_control
GSM5370824	Whole peripheral blood	paingroup: Resolved	type_chronic pain
GSM5370825	Whole peripheral blood	paingroup: Resolved	type_control
GSM5370826	Whole peripheral blood	paingroup: Resolved	type_chronic pain
GSM5370827	Whole peripheral blood	paingroup: Resolved	type_control
GSM5370828	Whole peripheral blood	paingroup: Resolved	type_chronic pain
GSM5370829	Whole peripheral blood	paingroup: Resolved	type_control
GSM5370830	Whole peripheral blood	paingroup: Resolved	type_chronic pain
GSM5370831	Whole peripheral blood	paingroup: Resolved	type_control
GSM5370832	Whole peripheral blood	paingroup: Resolved	type_chronic pain
GSM5370833	Whole peripheral blood	paingroup: Resolved	type_control
GSM5370840	Whole peripheral blood	paingroup: Resolved	type_chronic pain
GSM5370841	Whole peripheral blood	paingroup: Resolved	type_control
GSM5370842	Whole peripheral blood	paingroup: Resolved	type_chronic pain
GSM5370843	Whole peripheral blood	paingroup: Resolved	type_control
GSM5370844	Whole peripheral blood	paingroup: Resolved	type_chronic pain
GSM5370845	Whole peripheral blood	paingroup: Resolved	type_control
GSM5370854	Whole peripheral blood	paingroup: Resolved	type_chronic pain
GSM5370855	Whole peripheral blood	paingroup: Resolved	type_control
GSM5370858	Whole peripheral blood	paingroup: Resolved	type_chronic pain
GSM5370859	Whole peripheral blood	paingroup: Resolved	type_control
GSM5370866	Whole peripheral blood	paingroup: Resolved	type_chronic pain
GSM5370867	Whole peripheral blood	paingroup: Resolved	type_control
GSM5370868	Whole peripheral blood	paingroup: Resolved	type_chronic pain
GSM5370869	Whole peripheral blood	paingroup: Resolved	type_control
GSM5370874	Whole peripheral blood	paingroup: Resolved	type_chronic pain
GSM5370875	Whole peripheral blood	paingroup: Resolved	type_control
GSM5370876	Whole peripheral blood	paingroup: Resolved	type_chronic pain
GSM5370877	Whole peripheral blood	paingroup: Resolved	type_control
